# Measuring patient engagement with HIV care in sub‐Saharan Africa: a scoping study

**DOI:** 10.1002/jia2.26025

**Published:** 2022-10-26

**Authors:** Claire M. Keene, Ayesha Ragunathan, Jonathan Euvrard, Mike English, Jacob McKnight, Catherine Orrell, the InCARE Stakeholder Group

**Affiliations:** ^1^ Health Systems Collaborative Oxford Centre for Global Health Research Nuffield Department of Medicine University of Oxford Oxford United Kingdom; ^2^ Centre for Infectious Disease Epidemiology and Research School of Public Health and Family Medicine Faculty of Health Sciences University of Cape Town Cape Town South Africa; ^3^ Department of Medicine Faculty of Health Sciences University of Cape Town Cape Town South Africa

**Keywords:** adherence, engagement, evaluation, measure, retention, self‐management

## Abstract

**Introduction:**

Engagement with HIV care is a multi‐dimensional, dynamic process, critical to maintaining successful treatment outcomes. However, measures of engagement are not standardized nor comprehensive. This undermines our understanding of the scope of challenges with engagement and whether interventions have an impact, complicating patient and programme‐level decision‐making. This study identified and characterized measures of engagement to support more consistent and comprehensive evaluation.

**Methods:**

We conducted a scoping study to systematically categorize measures the health system could use to evaluate engagement with HIV care for those on antiretroviral treatment. Key terms were used to search literature databases (Embase, PsychINFO, Ovid Global‐Health, PubMed, Scopus, CINAHL, Cochrane and the World Health Organization Index Medicus), Google Scholar and stakeholder‐identified manuscripts, ultimately including English evidence published from sub‐Saharan Africa from 2014 to 2021. Measures were extracted, organized, then reviewed with key stakeholders.

**Results and discussion:**

We screened 14,885 titles/abstracts, included 118 full‐texts and identified 110 measures of engagement, categorized into three engagement dimensions (“retention,” “adherence” and “active self‐management”), a combination category (“multi‐dimensional engagement”) and “treatment outcomes” category (e.g. viral load as an end‐result reflecting that engagement occurred). Retention reflected status in care, continuity of attendance and visit timing. Adherence was assessed by a variety of measures categorized into primary (prescription not filled) and secondary measures (medication not taken as directed). Active self‐management reflected involvement in care and self‐management. Three overarching use cases were identified: research to make recommendations, routine monitoring for quality improvement and strategic decision‐making and assessment of individual patients.

**Conclusions:**

Heterogeneity in conceptualizing engagement with HIV care is reflected by the broad range of measures identified and the lack of consensus on “gold‐standard” indicators. This review organized metrics into five categories based on the dimensions of engagement; further work could identify a standardized, minimum set of measures useful for comprehensive evaluation of engagement for different use cases. In the interim, measurement of engagement could be advanced through the assessment of multiple categories for a more thorough evaluation, conducting sensitivity analyses with commonly used measures for more comparable outputs and using longitudinal measures to evaluate engagement patterns. This could improve research, programme evaluation and nuanced assessment of individual patient engagement in HIV care.

## 1 INTRODUCTION

With the increasing number of people initiated on antiretroviral therapy (ART) through the “universal test‐and‐treat” strategy, gaps in service provision are likely to shift from access to treatment to long‐term engagement with HIV care as the key modifiable mediator of treatment success [1], making the evaluation of engagement crucial to ensuring that the approach to the HIV epidemic is relevant and responds to the realities of people's experiences.

Evaluation drives policy decisions on the effective deployment of resources, making quality measurement vital in the health system's approach to control the HIV epidemic [2, 3]. Imprecise or inaccurate measures could undermine our understanding of engagement and how it mediates the impact of the personal, health system and contextual factors on treatment outcomes, or result in misclassification and incorrect targeting of interventions [4]. This makes it challenging to develop interventions to improve engagement or evaluate whether they are having the intended effect [5]. Strategic decisions can also be made more challenging by inaccurate interpretation of programme successes [6], misunderstanding the magnitude of the challenge [3] or inaccurate justification of costs [7, 8].

Accurate and valid measurement of engagement is challenging [1, 9, 10]. Despite its recognized importance and the numerous measures used in research and practice, there is no consensus or “gold standard” [11, 12]. As a result, estimates of engagement vary widely [13, 14] making conclusions difficult to interpret [15] and greater consistency a priority [16]. Additionally, engagement in HIV care is a complex, multi‐dimensional, dynamic process [17], with people moving in and out of care over time [18, 19] and requiring nuanced interventions to optimize it [20, 21]. New technologies and service structures, such as injectable antiretrovirals and multi‐month dispensing, will also change the dynamics of long‐term engagement. Therefore, the evaluation of engagement needs to reflect the changing dynamics of treatment [22], the shifting definitions of success [1] and the evolving needs of people on treatment [16] to drive service provision that facilitates long‐term, sustained engagement and treatment success [1].

Sub‐Saharan Africa has the highest prevalence of HIV in the world [23], reflecting a double burden of infectious and non‐communicable disease that disadvantages patients, places pressure on health systems and worsens dependency on donor funding [24]. Despite better rates of retention and adherence compared to high‐income settings [25, 26], challenges that influence the dynamics of HIV care engagement are particularly pervasive in the region [27]: widespread poverty and socio‐economic inequality, economic migration, gender power imbalances, low education rates, cultural beliefs that impact the interpretation of HIV and paternalistic patient–provider relationships [28, 29]. Additionally, the HIV epidemic also affects the general population rather than being concentrated in marginalized groups [30], although they are particularly vulnerable to HIV and its consequences [31].

The poor engagement has a significant impact on the health and wellbeing of all people living with HIV, as well as on the health system, with restricted second‐ and third‐line treatment options increasing the cost of managing poor outcomes [4]. Health systems also face many competing priorities that stretch the limited available resources, so are restricted in their ability to monitor engagement [32]. There is thus an urgency to support sustained treatment success and a need for feasible and reliable measures of engagement with care as a public health priority [33]. A scoping study was conducted to produce an organized, comprehensive set of indicators to measure engagement with HIV care in sub‐Saharan Africa, as a step towards making specific recommendations.

## 2 METHODS

### 2.1 Study design

A scoping study was conducted to map, summarize and categorize measures of engagement with HIV care (defined as all aspects of care for people who have initiated ART) in sub‐Saharan Africa, from a health service delivery perspective (i.e. dimensions that can realistically be measured by the health system). A scoping study was selected to synthesize knowledge as there is a large volume of heterogeneous literature on this topic [34]. It followed the “Arksey and O'Malley” framework [35] with the adaptations proposed by Levac et al. [36], and used the Joanna Briggs Institute guidance on conducting and reporting scoping reviews [37]. The protocol was registered on the Open Science Framework registry [38].

### 2.2 Search strategy and selection of the evidence

The concept of “engaging with care” was framed as a dynamic behaviour, reflected by observable, and thus measurable, activities: retention (interaction with health services), adherence (pill dosing behaviour) and active self‐management (ownership and self‐management of care), based on the “Indicators of HIV Care for Antiretroviral Engagement (InCARE)” framework [39]. These dimensions informed the search strategy and categorization of identified measures of engagement with HIV care. A wide search strategy was developed using key phrases from relevant articles [35], along the “population, context, concept” framework [40] (Table S1), to identify literature that explicitly or implicitly (through the description of the measure or its association with outcomes) engaged with the concept of measuring engagement with outpatient HIV services. The study used the search parameters and limits set out in Table 1 (see Table S2 for the justifications) and drew evidence from Ovid (Embase, PsychINFO and Global Health), PubMed, Scopus, CINAHL, Cochrane and the World Health Organization (WHO) Index Medicus, as well as free text searches on Google Scholar and identification of key literature through stakeholder input [41].

**Table 1:** Summary of the search parameters and limits as well as the final inclusion and exclusion criteria [35], categorized according to the “population, context, concept” search framework [40]

	Inclusion	Exclusion
**Search parameters and limits**		
	Published in English	Published in languages other than English
	Published between the start of 2014 and when the search was conducted in February 2021	Published before 2014
	Evidence from sub‐Saharan African settings	High‐resource settings and countries outside of sub‐Saharan Africa
**Eligibility**		
Literature	Peer‐reviewed publications, conference abstracts, guidance documents and reports, and systematic reviews with a pooled estimate	Letters, commentaries, editorials, opinion pieces and case reports
Population	Patients on lifelong ART or who have initiated ART previously (includes PMTCT option B+)	Pre‐ART initiation or people on pre or post exposure prophylaxis
	Adults ≥18 years old, including young adults and the elderly	Children and adolescent populations (included if adults 18 years and above are covered as well)
Context	Routine primary care or outpatient HIV clinic setting (including within a hospital setting). Includes measurement of engagement with HIV care within a trial setting	Hospital inpatient services or engagement with trials and research specifically
Concept	Measurement of engagement Engagement ‐Retention in services, adherence to treatment and active self‐management of care, as outlined by the InCARE framework Measurement ‐Primary purpose of the research was to evaluate, validate or compare measures of engagement, report on the performance of metrics or evaluate their association with ART‐related outcomes (such as virologic suppression, quality of life or drug resistance) ‐Used more than one metric for engagement and explicitly discussed this, compared them or combined them in a novel measure of engagement ‐Explicitly described, discussed or defined the measure of engagement, discussed proposed adjustments or explained how engagement was measured were included A lower threshold for inclusion was used for those that did not evaluate the measure but increased the scope of measures of engagement identified	Primary focus on associations of factors with an element of engagement or evaluation of an intervention, without defining the engagement element or explicitly stating how it is measured Focus on outcomes not related to ART success

Following the main search, all identified citations were collated and duplications removed using Mendeley Reference Manager and Rayyan [51], which was then used to screen all titles and abstracts. The 144 peer‐reviewed full texts (of the 182 full texts identified in the main search) were then screened using the eligibility criteria outlined in Table 1. A second reader evaluated a random sample of >15% of the titles/abstracts and >15% of the published full texts to clarify eligibility criteria and ensure consistency of inclusion [36]. Once the final criteria were established, each reader applied the clarified criteria to all literature screened and the inter‐rater agreement using Gwet's first‐order agreement coefficient (AC1) was reported for the final list [52, 53]. Disagreements were solved through discussion and consensus.

After preliminary analysis, grey literature identified in the main search (38 of the 182 full texts identified in the main search) and all literature from the secondary searches (peer‐reviewed and grey, *n* = 39) was reviewed for inclusion by the primary author, with a specific focus on identifying sources that increased the scope of engagement measures identified.

### 2.3 Data extraction, charting and synthesis

**Figure 1:**
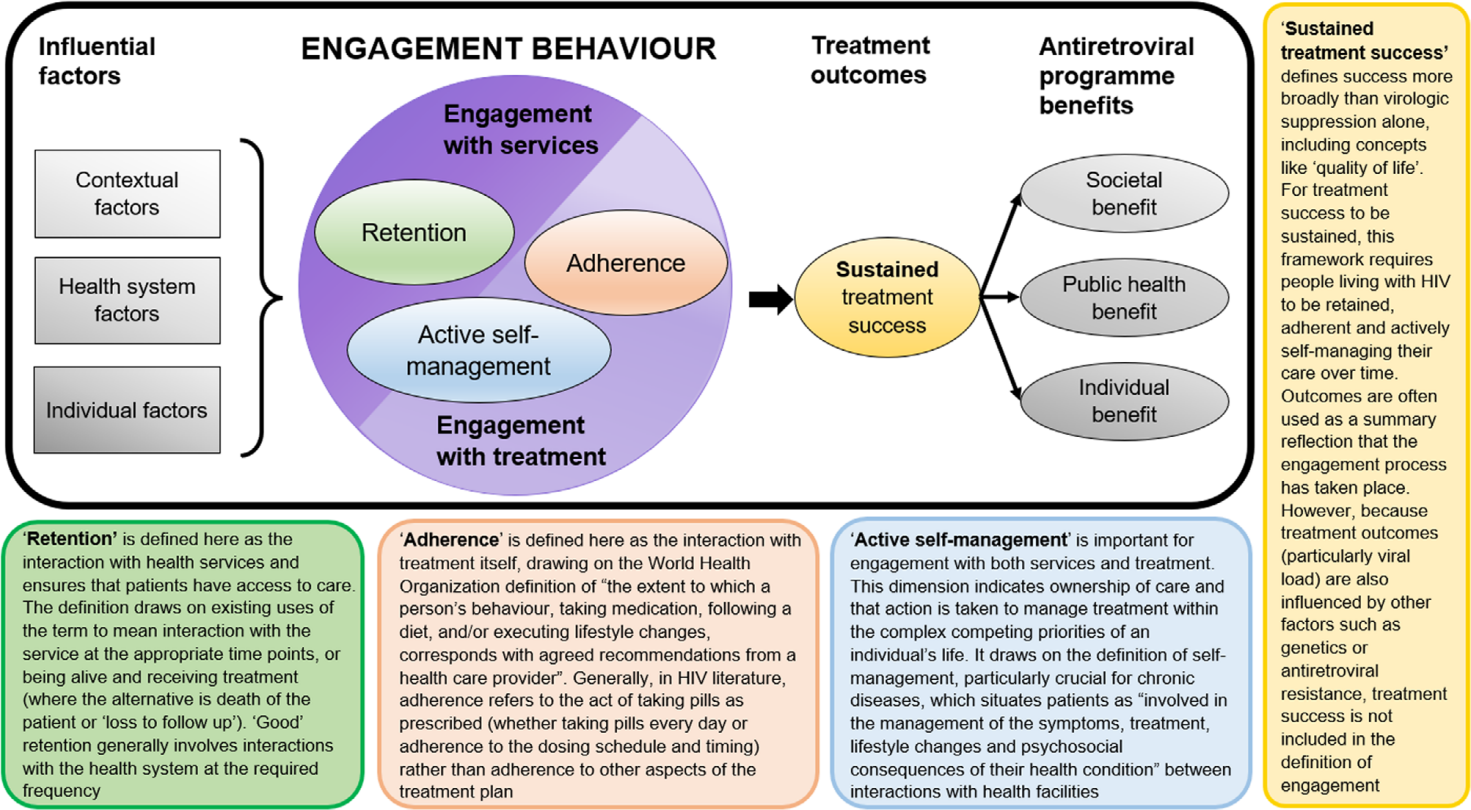
Indicators of HIV Care for Antiretroviral Engagement (InCARE) framework, used to direct the search and categorization stages of the scoping study for measures of engagement with HIV care in sub‐Saharan Africa [39], drawing from multiple definitions of retention [5, 42, 43], adherence [44, 45], active self‐management [24, 46, 47] and treatment outcomes [48, 49, 50].

The data extracted from each literature source included study information (including the year of publication, country, setting, participant characteristics and methods) and information on the measures of engagement (including information on the measure, such as definition, data collection, calculation and interpretation; evaluation of the measure's performance in predicting treatment outcomes; and how the measure was used or recommended to be used—Table S3). The extracted data were combined and organized by individual measure and categorized according to the retention, adherence and active self‐management dimensions of the InCARE framework (Figure 1) [39].

### 2.4 Stakeholder input

Stakeholder engagement is suggested as useful to add methodological rigour to scoping studies [36], thus the search terms, analysis and interpretation of the results were informed by feedback from the InCARE Stakeholder Group: 13 stakeholders, including HIV clinicians, academic researchers, programme implementers and Department of Health managers, with experience in sub‐Saharan Africa. The results presented include the stakeholder input.

## 3 RESULTS AND DISCUSSION

**Figure 2:**
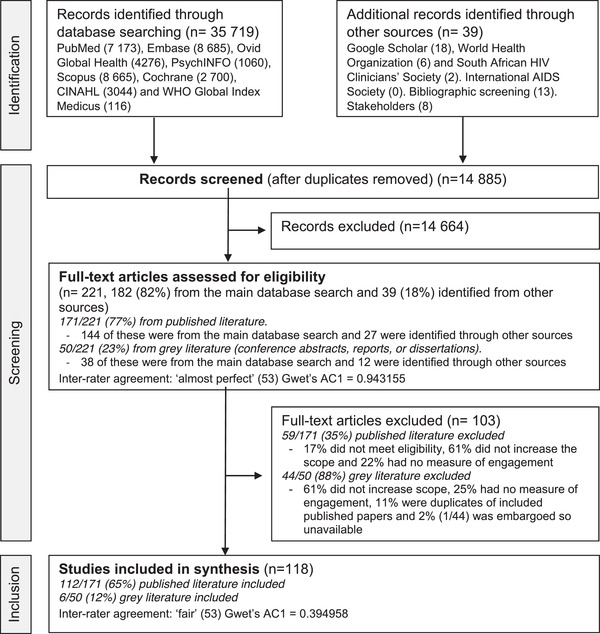
Preferred Reporting Items for Systematic Reviews and Meta‐analyses extension for scoping review (PRISMA‐ScR) flow diagram outlining the search and inclusion of the literature.

The main search was conducted on 17 February 2021. The results of the search and the study inclusion process are reported in Figure 2 according to the Preferred Reporting Items for Systematic Reviews and Meta‐analyses extension for scoping review (PRISMA‐ScR) flow diagram [54].

### 3.1 Characteristics of the evidence sources

After screening, 118 sources were included for analysis (Table S4). Cross‐sectional studies (35%) and cohort studies (36% prospective and retrospective combined) were the most commonly represented study types. Case controls, mixed methods, randomized and non‐randomized controlled trials, secondary analyses, modelling studies and systematic reviews made up the remaining 29% of sources. Most (69%) of the included studies evaluated the measure in some way, with 50% of the evidence directly evaluating the measure of engagement as the primary focus of the study.

Included evidence reflected research conducted in 19 individual countries in sub‐Saharan Africa. Nearly a third of the evidence involved participants from South Africa, with higher proportions from English‐speaking countries in southern and east Africa. Four sources had “global” multi‐country cohorts, which included sub‐Saharan African countries.

### 3.2 Identified measures of engagement

In total, 145 measures of engagement were extracted. After categorizing the measures and refining the framing of engagement with stakeholder input, 35 measures were removed as they were not felt to reflect engagement itself but rather factors affecting it (Table S5).

The remaining 110 measures of engagement were categorized by the element of engagement that they most strongly portrayed or were developed to reflect, and subsequently mapped onto the InCARE framework (Table 2). Some measures evaluated multiple dimensions of engagement or treatment outcomes as a summary measure that the engagement process had taken place. A full list of measures with information about their definition, duration and pattern of measurement, data collection, processing and evaluation (strengths, limitations and evidence for association with outcomes or other elements of engagement) can be found in the Tables S6–S10.

**Table 2:** Identified measures of patient engagement with HIV care, categorised according to the InCARE framework's dimensions of engagement^a^

Engagement with services	Engagement with treatment			Engagement with both services and treatment	
Retention (n=18)	Adherence (n=59)			Active self‐management (n=18)	Treatment outcomes (n=8)
**In care (n=5)** Visit attendance Average retention Engagement in care index Fixed point retention Re‐engagement retention **Continuity (n=7)** Visit gap/ treatment interruption Visit constancy The Health Resources and Services Administration HIV/AIDS Bureau (HRSA‐HAB) Continually retained Inter‐agency Task Team (IATT) definition Visit pattern Retention trajectory **Timing of retention (n=3)** Late for visit Time to loss to follow‐up Appointment intervals **Composites of retention measures (n=3)** Point retention and missed visits Point retention and average retention On time adherence retention	**Primary adherence (n=5)** *(prescription filled)* **Pharmacy refill (n=5)** Pharmacy visit compliance/ crude pharmacy interval Medication possession ratio (MPR) Poor adherence proportion Sustained poor adherence Pharmacy refill gaps **Secondary adherence (n=54)** *(medication taken as directed)* **Antiretroviral concentrations (n=6)** Blood levels Intracellular levels Hair levels Urine levels Saliva levels Antiretroviral detection of intermittent engagement **Laboratory tests (n=5)** Haemoglobin Mean cellular volume (MCV) difference Bilirubin Cathepsin Resistance detection of intermittent engagement **Healthcare‐worker‐assessed measures (n=2)** Clinician‐recorded adherence Directly observed therapy (DOTs)	**Pill counts (n=5)** Electronic medication event monitoring system (MEMS) Adherence trajectory by MEMS Healthcare worker pill counts Pill count variance Over‐adherence by pill count **Self‐reported quantification of pill‐taking through recall (n=13)** Visual analogue scale (VAS) Self‐reported ART interruption Weekend recall Two‐day recall Three‐day recall Four‐day recall One week recall Two week recall Combined one to two week self‐report Recall since previous visit Pill recall and interruption Swiss HIV Cohort Study Adherence Questionnaire (SHCS‐AQ) Adherence trajectory by self‐report **Self‐reported timing (n=2)** Schedule adherence ART adherence while drinking pattern questionnaire	**Composite self‐reports (n=13)** Center for Adherence Support Evaluation (CASE) Index Score Composite of recall and ability 3 item self‐report composite of ability and recall South African National Department of Health Adherence Questionnaire Pattern of adherence by self‐report VAS‐SMAQ‐FDR VAS‐SHCS‐AQ VAS‐SMAQ‐ID Godin's self‐report Adherence to Refills and Medication Scale (ARMS‐7) Simplified Medication Questionnaire (SMAQ) Adult AIDS Clinical Trials Group (AACTG) adherence questionnaire AACTG derivative **Composites of adherence measures (n=8)** Global adherence Composite adherence score (CAS) MPR‐pill recall Composite of antiretroviral concentration and self‐report Behavioural adherence measure Composite of pharmacy refill measures CD4 stratified by MCV On time – pill count	**Active involvement (n=4)** Action planning Health‐seeking behaviour Patient activation (PAM‐13 scale) Patient‐provider interaction quality **Self‐care and self‐management (n=14)** Measures of Drug Self‐Management Scale (MeDS) Self‐reported ART self‐management Practice of self‐management Composite self‐management outcome Adolescent HIV Self‐management scale (AdHIVSM scale) HIV Self‐Management Inventory (SMI) Revised self‐care symptom management strategies (SSC‐ HIVrev) Self‐care practices Self‐care management Three‐item self‐regulation Four‐item self‐regulation Social facilitation Coping	**Laboratory results (n=5)** Viral suppression Sustained low level viraemia CD4 change CD4 trajectory Antiretroviral resistance **Quality of life and health status (n=3)** HIV mortality HIV specific quality of life Self‐reported health status

**Multi‐dimensional engagement (n=7)** (composites of different elements of engagement)		
Composite measure of engagement (adherence and retention) Composite outcome of retention and viral suppression	Engagement score (retention, adherence and clinical status) Engagement trajectory (adherence and retention)	Summary score to predict viral failure Viral Load Testing Criteria (VLTC) Characterisation of patient stability

^a^ References for each measure are found in Supplementary material Tables 6‐10 with the full information on the measures and the evidence associated with them

### 3.3 Identified use cases

The situations in which measures of engagement were used or recommended to be useful were noted for each study (Table S4). The categories of use cases were reviewed with stakeholders and the final categorization is presented in Table 3. It was noted that measures may be useful in an ideal world without current resource constraints or would have used for the practical assessment.

**Table 3:** Three overarching use cases and the nine specific applications for each found in the literature and reviewed with stakeholders

Overarching use case:	Routine monitoring and evaluation of programmes^a^ [2, 5, 13, 15, 22, 49, 55, 56, 57, 58, 59, 60, 61, 62, 63, 64, 65, 66, 67, 68, 69, 70, 71]	Individual patient evaluation [71, 72, 73, 74, 75, 76]	Research to draw conclusions for recommendations
Specific use case applications:	Routine evaluation of engagement in patients in facility care [6, 61, 62, 77, 78]	Rationalize resources, such as expensive genotype [33, 79, 80, 81, 82] or viral load [50, 82, 83, 84, 85, 86, 87] testing and resources to trace those lost to follow‐up [88]	Understanding the behaviour of people on ART [15, 21, 25, 63, 65, 89, 90, 91, 92, 93, 94, 95, 96, 97, 98, 99, 100, 101, 102, 103, 104, 105, 106, 107, 108, 109, 110, 111, 112, 113, 114, 115, 116, 117, 118]
	Routine evaluation of engagement in patients in differentiated service delivery models [49, 119]	Flag patients early for intervention [57, 120, 121, 122, 123, 124, 125, 126, 127, 128, 129]	Identification of factors on which to intervene [4, 63, 65, 89, 90, 92, 93, 97, 101, 104, 106, 110, 111, 116, 130, 131, 132, 133]
	Routine evaluation of engagement in patients who struggle with care [134]	Support and direct ART management decisions (through initiating conversations with patients or providing healthcare workers with information) [4, 9, 10, 45, 75, 86, 87, 135, 136, 137, 138, 139]	Development and evaluation of interventions to support optimal engagement [24, 32, 133, 140, 141, 142, 143, 144, 145, 146, 147, 148, 149, 150, 151, 152, 153]

^a^ At facility and population levels for quality improvement and strategic decision‐making.

### 3.4 Overview of results

While it is widely accepted that “engagement in care” is critical to achieve treatment success in ART programmes [154, 155, 156], there is little clarity on how best to measure it. This study scoped how health services and researchers measure HIV care engagement in sub‐Saharan Africa. It attempts to improve standardization of the measurement of engagement by identifying and collating information on 110 measures and categorizing them into five groups: measures reflecting retention in care, medication adherence, active self‐management, multi‐dimensional engagement and treatment outcomes. In addition, this study categorized the purpose of measurement into three over‐arching use cases. Some of the challenges and considerations in measuring engagement encountered in the course of this scoping study are outlined and recommendations on evaluating engagement with this evidence are made.

### 3.5 Use cases

Ultimately, the most appropriate measure depends on the intended purpose of the evaluation [22]. The use cases overlap with the dimensions of engagement with HIV care: different measures are suited to provide the depth of knowledge required to answer different questions, justifying varying burdens of data collection and analysis. If the purpose is to evaluate engagement at a population or programme level for quality improvement or to make strategic policy decisions [157], inexact estimates of retention and viral suppression (treatment outcome) may be sufficient to infer credible conclusions, allowing directional decisions to be made [59].

As the gaps in service provision shift from simple treatment availability issues to the complex maintenance of engagement over time [1], more subtle interventions tailoring support to people's specific needs are required [16]. If the purpose of measurement is research to make causal inferences, more in‐depth evaluation is needed: to develop nuanced interventions, understand the mechanisms of their impact and make decisions on implementation [20, 21]. Similarly, directing individual management needs a much more refined understanding of who has, or is likely to have, trouble engaging, in order to make good decisions about which intervention to implement for a particular patient [50].

### 3.6 Measures of retention

Retention measures reflected interactions with health services, evaluating whether a person was in care, the continuity of the retention or the timing of the visit (lateness relative to a scheduled/expected visit). Retention measures can be derived from routine data [88] and simple measures like average retention (proportion of kept/expected visits attended) make the evaluation of retention a pragmatic option to monitor HIV programmes [5]. Most retention measures were discussed in multiple sources, but multiple thresholds within each measure reduced standardization as different papers used different definitions of “retained” versus “not retained”/“lost to follow‐up” (from 9 weeks [88] to 180 days [49] without a visit). Different definitions can result in very different conclusions and the recall period can change the measure of success, worsening the outcomes of more recent time periods [5]; highlighting the importance of being intentional about how we measure retention for specific purposes [22].

Fixed point retention was one of the most ubiquitously used measures to consider people as “retained” or “lost to follow‐up” (also termed “attrition”). However, it does not capture the pattern of attendance, reflect the continuity of care or capture milestones before the point of analysis [6, 158], misclassifying intermittently engaged people as “retained”/“lost to follow‐up,” depending on when the evaluation was conducted in their trajectory [66]. The dynamic nature of engagement and the reality of people churning in and out of care are important considerations for both routine evaluation and better understanding of interventions and individual management [66]. In one example, 54–98% were misclassified compared to a composite continuity of retention measure, depending on the definition of loss to follow‐up [6].

Retention measures are also limited as they are generally calculated retrospectively: data sources poorly distinguish between the loss to follow‐up and death [158] and often miss “silent transfers” between different facilities [158]. This can be addressed by following patients to evaluate alternate outcomes and adjust estimates: either through tracing a sample [57], using weights from the literature [27, 134] or using national/combined databases to follow patients who move between facilities [3, 158]. However, all these approaches increase the burden of data collection, linkage and analysis, reducing their feasibility in practice.

Different sources of data demonstrated different strengths in estimating retention even within the same population: in one study, laboratory data underestimated the proportion retained compared with single clinic visit data, but the centralized system could evaluate retention across facilities to detect “silent transfers” [5]. Data can be triangulated to compensate for the weaknesses of individual sources [78]: retention in care using “any evidence” of engagement (pharmacy, clinic visit or laboratory evidence) from all available sources was shown to produce a higher estimate of retention than the individual sources alone [5, 146]. Advancement and merging of electronic databases may facilitate the use of combined data sources and improve the quality of estimates of retention (and other measures of engagement) [6, 157], but require investment and thought to address issues around the protection of personal information.

### 3.7 Measures of adherence

Measures of adherence made up more than half the identified metrics. These measures reflected primary (prescription filled) and secondary adherence (medication taken as directed) [55]. Assessing adherence is useful in research to understand engagement behaviour [160] and for individual management: particularly identifying the need for and directing interventions before virological failure is established [161], and for rationalizing expensive genotypic resistance testing for people failing second‐line ART [79, 150]. These measures need to be conducted specifically to evaluate adherence, making them less suited to routine programme monitoring.

Many studies attempted to find or evaluate “more objective” measures of adherence to overcome the social desirability and recall biases associated with self‐reported measures [4, 82, 93, 136], which may result in overestimation of adherence [10, 72, 84]. Some measures were considered more objective than self‐reports [55, 62, 75, 79, 159], but even these remain indirect measures of behaviour taking place outside the facility [55]: laboratory tests use changes associated with ART as proxies [81, 87], pill counts measure whether pills were removed from the bottle rather than if they were taken [55] and pharmacy refills reflect a maximum possible level of adherence through ART on hand, leading to possible overestimation of “true” adherence [2, 93]. Antiretroviral concentrations were considered to quantify adherence independent of other influences such as resistance [10] and were often used as an “objective” gold standard to compare other measures of adherence against [160]. However, they were not consistently associated with viral outcomes [67, 72, 135].

The generally poor ability of adherence measures to detect viral non‐suppression was demonstrated in a recent Cochrane review, which found a wide variation in sensitivity and specificity across measures [161]. Adherence measures can also be influenced by the particular drug [137], body weight, genetics, metabolism [75, 76] and “white coat adherence” (temporary improvement in adherence prior to a clinical visit) [15, 137]. White coat adherence particularly applies to blood and urine ART concentrations [113], which reflect a shorter duration of adherence (3–5 days) [100, 137, 160] than hair (1 month/1 cm hair [33, 117, 138]). Tenofovir, emtricitabine and tenofovir diphosphate in dried blood spots are growing in popularity to measure adherence, but the costs and turnaround time for results still prohibit scale‐up [162]. Point‐of‐care assays for urine tenofovir are affordable and could be used to support adherence discussions in real‐time, but patient acceptability is mixed [163].

While bias limits their ability to detect the “true” state of adherence, self‐reports do offer a person's perception of their adherence [164] and identify adherence concerns even in the presence of current virologic suppression [121]. They may have lower sensitivity and be insufficient to detect most cases of poor adherence, but like refill measures, they have high specificity: those self‐identified as non‐adherent warrant investigation [139]. Monitoring change in self‐reported adherence improved sensitivity [86] and self‐reports can trigger a discussion within a consultation with a shared understanding of engagement between the patient and healthcare worker [121]. Self‐reports are also low cost, easily implemented [15] and can discriminate between intentional and unintentional non‐adherence [61], especially useful for individual patient evaluation to direct the management plan.

### 3.8 Measures of active self‐management

Measures of active self‐management fell into two main categories: (1) items that measure action‐oriented health‐related behaviours and active involvement in the treatment plan [165], and (2) items that reflect whether people are managing the treatment plan themselves. This encompassed self‐care, self‐monitoring, symptom management, management of other activities that maintain their health [166] and self‐management: medical management, the management of their new role as a patient and maintaining emotional health [46]. The active self‐management measures are not currently part of routine care, making them less suitable for programmatic monitoring, but potentially valuable for evaluating individual engagement issues to direct care, and researching more refined interventions to support engagement.

The goals of success are slowly shifting from simply providing access to ART, to the complex task of keeping people on treatment lifelong [1] regardless of changes in the health system, people's personal lives and the interplay between the two [3, 167]. We hypothesize that this aspect of engagement will be increasingly important in ensuring retention and adherence over time to maintain long‐term successful treatment outcomes. However, because the measurement of this dimension has not been a priority in sub‐Saharan Africa [121], there is currently little evidence to support this. Despite making up the same proportion of the identified measures as retention, there was little overlap in active self‐management with only one measure (Adolescent HIV Self‐management scale) discussed in more than one source (though both by the same author [96, 168]. If concepts are not measured, there is little evidence to justify that they matter and to promote their subsequent routine measurement: creating a difficult loop to break. Further evaluation of active self‐management measures is needed to support their wider adoption into practice. This study provides a starting point: an organized list of options that have been implemented in sub‐Saharan Africa and could be used and evaluated more widely.

### 3.9 Multi‐dimensional engagement

Some measures combined dimensions of engagement, such as adherence and retention [57, 142] or retention and treatment outcomes [77, 122, 144, 146, 149], where people categorized as “engaged” met the criteria for all components [122]. Adherence and retention trajectories were combined to identify an additional group of people who had consistent retention but early non‐adherence (measured by medication possession ratio) that was not identified when each dimension was evaluated separately [57]. Measuring multiple dimensions of engagement can help to evaluate engagement more comprehensively, and may offer a simple approach to flag issues with engagement: if engagement is not optimal, individual dimensions can then be evaluated.

These measures, however, require the collection of multiple pieces of information, are more complex to calculate, may obscure issues with individual engagement dimensions when combining them and may not always improve the accuracy of the measurement [161]. Thus, the actual additional benefit needs to be balanced with complexity and feasibility when evaluating multiple dimensions in a single measure.

### 3.10 Treatment outcomes as a measure of engagement

The retention, adherence and active self‐management dimensions categorize engagement behaviour, which in turn drives the success or failure of antiretroviral treatment. Treatment outcomes are thus a consequence of engagement and represent a summary of whether the engagement process has taken place. For example, an individual must have consistently managed their appointments and taken pills over time to have a viral load (VL) test result and be virologically suppressed [49]. In this study, the identified measures of engagement were often evaluated against treatment outcomes, with those showing stronger associations deemed more accurate reflections of retention [5], adherence [75, 120, 139] and active self‐management [175]. Virologic suppression, in particular, was often cited as the “gold standard” of treatment success [63, 169, 183].

Identified measures of treatment outcomes included VL, immunological outcomes, antiretroviral resistance, mortality, health status and HIV‐specific quality of life, which could be used to broaden the definition of treatment success beyond the narrow focus on virologic suppression [48]. Disability‐adjusted life years (DALYs) and quality‐adjusted life years (QALYs) are used extensively in modelling studies and evaluations of other chronic diseases [170], but they have not found a routine place in HIV evaluation: no included sources mentioned DALYs or QALYs. These are metrics that the HIV community could adopt to increase the comparability of evaluations.

While engagement is necessary to achieve sustained treatment success, outcomes are also influenced by factors, such as ART resistance, drug–drug interactions or suboptimal pharmacokinetics [50]. Therefore, outcomes cannot discriminate between poor engagement or deterioration due to other reasons [137]. The delayed effect between suboptimal engagement and a change in outcomes (e.g. VL [120]) makes outcomes poor indicators of early engagement issues when intervention could avert the need to switch regimens to less tolerable second‐ or third‐line options. VLs are also relatively expensive and are not always available for routine monitoring [50, 82]: at best, they are conducted infrequently (e.g. yearly), intermittently or at worst not at all [62]. Additionally, most programmes only begin monitoring VL from 4 to 6 months after initiation [171, 172], missing a high‐risk period for disengagement [155].

Additionally, treatment outcomes do not differentiate between the dimensions of engagement. VL has been used specifically as a measure of adherence [4, 86, 173], but this too is subject to misclassification bias [70], and a missing VL may reflect healthcare worker error, resource constraints or poor retention rather than adherence. Relying on a primary outcome of virologic suppression for evaluation in research could misrepresent the efficacy of new interventions, which may improve a dimension of engagement but not be sufficient alone to improve treatment outcomes [174].

VL continues to be a valuable measure at multiple levels of the system and the WHO supports the expansion of regular VL monitoring [175]. However, this needs to be interpreted within its limitations and supplemented with measures of individual dimensions to comprehensively evaluate engagement. In the absence of a single best measure of engagement, we recommend the evaluation of more than one dimension from the InCARE framework to measure engagement more comprehensively. This could help better understand engagement behaviour, develop appropriate interventions and make better decisions about individual patient management.

### 3.11 Alternative approaches to current measures of engagement

Combining measures has been proposed as an alternative to a single best measure of engagement. For example, a comparison of short‐ and long‐term measures of adherence can identify intermittent or “white coat” adherence [137], and composite retention measures capture visit consistency, are more stringent and have lower misclassification than fixed point retention [6]. While combinations improved sensitivity in some cases, this did not substantially improve the association with outcomes, adding complexity without fully overcoming individual measures’ limitations [16, 122]. This was also demonstrated by a recent Cochrane review that found that the sensitivity of composite measures of adherence ranged from 10% to 100%, and specificity from 49% to 100% [161]. It may be most feasible to use simple measures with moderate association with outcomes and interpret them within their limitations [4].

Adherence measures were widely evaluated as an alternative to costly VL monitoring, but generally, the association was not strong enough to replace it with confidence. Associations varied with the threshold used for “good” adherence, changing the clinical interpretation when making decisions [50, 139]. In addition, newer adherence measures, such as electronic medication event monitoring systems and therapeutic drug monitoring, can be more expensive than VL, with complicated logistics (e.g. sample storage at –80°C [137, 160]) and requirements for complex equipment [15, 55, 135, 137, 160] that reduce their feasibility in practice.

Cross‐sectional measurements fail to identify the gaps in the treatment journey when the health service could intervene to improve engagement [42, 57]. Longer periods of evaluation capture more of the subtlety of engagement, are more strongly associated with outcomes [139, 160] and can facilitate the evaluation of patterns of engagement. Measuring patterns avoids obscuring the individual differences in engagement over time that occurs with single time‐point measures [57, 60], potentially useful in understanding engagement dynamics [49, 58]. Evaluation of individual‐level longitudinal trajectories of engagement can also uncover “behavioural phenotypes” that identify high‐risk individuals in high‐risk periods of their treatment journey [57, 123], offering a novel opportunity to direct interventions to behavioural patterns rather than the demographic categories we traditionally use to target differentiated services (often with poor success) [57]. These prospects make these measures worth the required longer follow‐up [6], adjustments to data collection to track patients and link data from different sources [157] and the added computational complexity [5]. Investments in routine databases could support better measurement both for clinical patient management and routine programme monitoring, and provide data for research analyses [176].

### 3.12 The “elusive gold standard”

Considering the number of sources this study screened, it is surprising that no standard definitions of an engagement or consistently used measures (even for retention [42] or adherence [50]) were identified. While VL is considered the gold standard for monitoring treatment response [50], the mixed evidence means no measure perfectly reflects successful engagement or its dimensions. Measures are chosen to prioritize sensitivity or specificity in predicting outcomes, resulting in a trade‐off between missing people in need of support or over‐intervening and wasting resources [125].

There is a demand for specific recommendations on standardized measures to measure engagement consistently and comparably [14, 177, 178]. In large ART programmes in sub‐Saharan Africa, measurement choice is restricted by practicality [50, 121]. Thus, consideration of feasibility (specifically cost, complexity and time burden) is paramount for practical integration into routine health monitoring systems [136]. Until more specific recommendations are produced, we recommend conducting sensitivity analyses using multiple common definitions of retention (such as visit gaps and fixed‐point retention), adherence (medication possession ratio, electronic pill count, self‐report and antiretroviral concentration) and active self‐management (patient activation and self‐management assessment) to increase the comparability of evaluations of engagement. The use of multiple measures could also explore the implications of different measures on the conclusions drawn, particularly for the dimension of active self‐management, for which there is less evidence [121].

While the search for a unifying measure reflective of engagement was unsuccessful, this indicates the complexity of the concept [17]. Engagement is multi‐dimensional, with retention, adherence and active self‐management all crucial to long‐term, sustained treatment success [39]. Particularly for the purposes of making decisions on individual patient care and research to develop and test new interventions, evaluating all dimensions may be more valuable than hiding the heterogeneity with summary measures.

### 3.13 Strengths and limitations

This study focused specifically on engagement with HIV care, but the findings could inform the evaluation of other chronic diseases requiring lifelong engagement with care. Strengths of this study also include a broad search of the literature across multiple databases and the review of a large number of sources. This study drew on the experience of the research team in clinical HIV management and differentiated service development in low‐resource, contextually challenging settings, grounding this work in the practical realities of patient care and programme management. Eligibility criteria, data extraction, analysis and results were continually reviewed throughout the scoping process to reduce bias, both with the second reader and stakeholder engagement as suggested by scoping study guidance [35, 36].

The research question was very broad, both in the concept of engagement and the number of papers identified: a recognized drawback of scoping studies [36]. This study did not attempt to evaluate the quality of sources and whether they reflected the underlying construct. Some useful measures may have been missed through the focus on sub‐Saharan Africa, and measures from high‐income or other low‐income settings could inform the measurement of engagement in sub‐Saharan Africa. As literature was reviewed, terms that described engagement behaviour were identified that had not been included in the original search, and the Gwet's AC1 for the studies included was “fair,” with 68% observed agreement, reflecting the lack of clarity in the definition of engagement. In addition, due to the English language limit in the search, most evidence was from English‐speaking countries in southern and east Africa. However, restricting the search to “English” only reduced the volume of evidence by a small amount (e.g. by 0.6% for PubMed), which may reflect that English is the main language for scientific publication [179], or that countries with a prevalence above 10% are all anglophone [180]. Surprisingly, the two sub‐Saharan African countries with the highest HIV prevalence in the world, Eswatini and Lesotho [180], did not produce literature that was identified in this search, despite being searched for by name.

Of the 145 measures of engagement identified in this study, 35 were removed from the final list as they were judged to reflect factors that affect engagement rather than engagement behaviour itself. These included contextual factors (e.g. measures of the reasons for non‐adherence), reflections of the health system (e.g. ART coverage) and personal factors (e.g. the ability to engage, including information, motivation and behavioural skills). The line between engagement behaviour and the factors affecting it was a persistent tension in discussions throughout screening and stakeholder engagement. The ambiguity in the definition of the measures of engagement dimensions meant that the process of refining the categorization of identified measures was iterative.

## 4 CONCLUSIONS

Heterogeneity in conceptualizing HIV care engagement is reflected by the broad range of measures identified and the lack of consensus on the best indicators of each dimension. The purpose of evaluation should direct the choice of measurement: research, programme evaluation and patient assessment could all be advanced through measurement of multiple dimensions for a more comprehensive evaluation, conducting sensitivity analyses with commonly used measures for more comparable outputs and using longitudinal measures to evaluate patterns of engagement. Improvements to data collection and management could also facilitate better routine measurement of these engagement dimensions to facilitate improved individual patient management, programme monitoring and research to explore the impact of interventions on engagement and treatment outcomes.

This review categorized the wide variety of measures used to evaluate engagement with HIV care in sub‐Saharan Africa into a usable reference list. It could help make choices on measuring engagement for different use cases, and provides options for the less commonly evaluated dimension of “active self‐management.” However, specific recommendations could not be made from the available evidence as no measures were obviously superior—for engagement overall, for individual dimensions or to replace virological outcome monitoring. Further work could make evidenced recommendations on a standardized, minimum set of measures to comprehensively evaluate engagement with care for different use cases. This study could also support further work to explore the importance of the active self‐management dimension, unpacking the underlying mechanisms of poor engagement and differentiating those who are unwilling to engage from those who are unable to remain engaged due to complex individual, social and health system factors. An improved understanding of the mechanisms driving disengagement and how to evaluate engagement comprehensively could support the implementation of more nuanced interventions to improve it. It could also inform the understanding and measurement of engagement for other chronic diseases.

## COMPETING INTERESTS

There are no relevant financial or non‐financial competing interests to report.

## AUTHORS’ CONTRIBUTIONS

Conceptualization: CMK, ME, JMK and CO.

Methodology: CMK, AR, JE, ME, JMK and CO.

Data curation: CMK.

Analysis: CMK, AR, JE, ME, JMK, CO, InCARE Stakeholder Group: AG, BH, EvdH, IEW, IK, KA, KRA, LB, MM, TC and TP.

Funding acquisition: N/A.

Writing—original draft: CMK.

Writing—review and editing: CMK, JE, AR, ME, JMK, CO, InCARE Stakeholder Group: AG, IEW, IK, KA, KRA, LB, MM and TP.

## FUNDING

This research was conducted as part of a PhD undertaken by CK at the University of Oxford. It was supported through a scholarship from the Clarendon Fund and St John's College Kendrew Clarendon Award, in partnership with the Nuffield Department of Clinical Medicine.

## Supporting information

Additional files
**Table S1**: Example search strategy for the PubMed database.
**Table S2**: Justification for the search parameters, search limits and eligibility criteria for inclusion of sources in the scoping study.
**Table S3**: Data extracted from the included sources identified in the search.
**Table S4**: List of included sources.
**Table S5**: List of measures removed during analysis.
**Table S6**: Measures of retention with detailed information on their use.
**Table S7**: Measures of adherence with detailed information on their use.
**Table S8**: Measures of active self‐management with detailed information on their use.
**Table S9**: Measures of multi‐dimensional engagement with detailed information on their use.
**Table S10**: Measures of treatment outcome with detailed information on their use.

**File S1**: Members of the InCARE Stakeholder Group.

## Data Availability

Data are available on request.
