# Supplementary material for: Measuring patient engagement with HIV care in sub‐Saharan Africa: a scoping study
Source: J Int AIDS Soc. 2022 Oct 26;25(10):e26025. doi: 10.1002/jia2.26025 (PMC9597383; doi:10.1002/jia2.26025)
Supplement: Supplementary file 1 — Additional files Table S1: Example search strategy for the PubMed database. Table S2: Justification for the search parameters, search limits and eligibility criteria for inclusion of sources in the scoping study. Table S3: Data extracted from the included sources identified in the search. Table S4: List of included sources. Table S5: List of measures removed during analysis. Table S6: Measures of retention with detailed information on their use. Table S7: Measures of adherence with detailed information on their use. Table S8: Measures of active self‐management with detailed information on their use. Table S9: Measures of multi‐dimensional engagement with detailed information on their use. Table S10: Measures of treatment outcome with detailed information on their use. [file JIA2-25-e26025-s002.docx]

Measuring patient engagement with HIV care in sub-Saharan Africa: a scoping study

September 2022

Supplementary material

Contents

[Table 1: Example search strategy for the PubMed database 2](#_Toc113541630)

[Table 2: Justification for the search parameters, search limits and eligibility criteria for inclusion of sources in the scoping study 4](#_Toc113541631)

[References for Table 2 5](#_Toc113541632)

[Table 3: Data extracted from the included sources identified in the search 6](#_Toc113541633)

[Table 4: List of included sources 7](#_Toc113541634)

[Table 5: List of measures removed during analysis 37](#_Toc113541635)

[Table 6: Measures of retention with detailed information on their use 38](#_Toc113541636)

[Table 7: Measures of adherence with detailed information on their use 48](#_Toc113541637)

[Table 8: Measures of active self-management with detailed information on their use 84](#_Toc113541638)

[Table 9: Measures of multi-dimensional engagement with detailed information on their use 95](#_Toc113541639)

[Table 10: Measures of treatment outcome with detailed information on their use 100](#_Toc113541640)

[References for Tables 6-10 104](#_Toc113541641)

## Table 1: Example search strategy for the PubMed database

| Category | Search terms |
| --- | --- |
| HIV terms | ("HIV"[Mesh] OR (HIV[Title/Abstract]) OR (Human Immunodeficiency Virus[Title/Abstract])  OR "Anti-Retroviral Agents"[Mesh] OR "Antiretroviral Therapy, Highly Active"[Mesh] OR (Antiretroviral[Title/Abstract]) OR (Anti-retroviral[Title/Abstract]) OR (HAART[Title/Abstract]) OR (ARV[Title/Abstract]) OR (ARVs[Title/Abstract])) |
| AND | |
| Outpatient terms | ("Ambulatory Care Facilities"[Mesh] OR (ambulatory care facilit*[Title/Abstract])  OR (Care[Title/Abstract]) OR (Clinic*[Title/Abstract]) OR (Service*[Title/Abstract]) OR (manage*[Title/Abstract]) OR (Polyclinic[Title/Abstract]) OR (policlinic[Title/Abstract]) OR (Outpatient*[Title/Abstract]) OR (Day Hospital*[Title/Abstract])  OR "Primary Health Care"[Mesh] OR (Primary healthcare[Title/Abstract]) OR (Primary Care[Title/Abstract]) OR "Delivery of Health Care"[Mesh] OR (delivery of healthcare[Title/Abstract]) OR (healthcare delivery[Title/Abstract]) OR (Healthcare System[Title/Abstract])  OR "Pharmacies"[Mesh] OR (pharmacy[Title/Abstract]) OR (pharmacies[Title/Abstract]) OR (Pharmaceutical Service[Title/Abstract]) OR (chemist[Title/Abstract]) OR (Chemists[Title/Abstract]) ) |
| AND | |
| Measurement terms | ("Statistics as Topic"[Mesh] OR (Statistic*[Title/Abstract]) OR (Approach*[Title/Abstract])  OR (metric*[Title/Abstract]) OR (measur*[Title/Abstract])  OR "Process Assessment, Health Care"[Mesh] OR (Assess*[Title/Abstract]) OR (Monitor*[Title/Abstract]) OR "Validation Studies as Topic"[MeshExa] OR (Validat*[Title/Abstract]) OR (Performance[Title/Abstract]) OR (Quality[Title/Abstract]) OR (Evaluat*[Title/Abstract])  OR (Comparison[Title/Abstract]) OR (Comparative[Title/Abstract]) OR (Compare*[Title/Abstract]) OR (Comparing[Title/Abstract]) OR (Standard*[Title/Abstract])  OR "Patient Outcome Assessment"[Mesh] OR (Outcome*[Title/Abstract])  OR (Feasib*[Title/Abstract]) OR (Continuum[Title/Abstract]) OR (Cascade[Title/Abstract])) |
| AND | |
| Engagement terms | ("Retention in Care"[Mesh] OR (Retention[Title/Abstract]) OR (nonretention[Title/Abstract]) OR (non-retention[Title/Abstract]) OR (non retention[Title/Abstract]) OR "No-Show Patients"[Mesh] OR (attendance[Title/Abstract]) OR (Loss to follow up[Title/Abstract]) OR (Loss to followup[Title/Abstract]) OR (Loss to follow-up [Title/Abstract]) OR (nonattendance[Title/Abstract]) OR (non-attendance[Title/Abstract]) OR (non attendance[Title/Abstract]) OR "Appointments and Schedules"[Mesh] OR "Office Visits"[Mesh] OR (visit*[Title/Abstract]) OR (Appointment*[Title/Abstract]) OR (Pharmacy refill[Title/Abstract]) OR (Gap in care[Title/Abstract]) OR (Treatment interruption[Title/Abstract]) OR "Medication Adherence"[Mesh] OR "Patient Compliance"[Mesh] OR "Treatment Adherence and Compliance"[Mesh] OR (adherence[Title/Abstract]) OR (compliance[Title/Abstract]) OR (non-adherence[Title/Abstract]) OR (nonadherence[Title/Abstract]) OR (non-compliance[Title/Abstract]) OR (noncompliance[Title/Abstract]) OR "Drug Monitoring"[Mesh] OR (drug monitoring[Title/Abstract]) OR (medication monitoring[Title/Abstract]) OR (Medication persistence[Title/Abstract]) OR (Drug concentration*[Title/Abstract]) OR (Antiretroviral concentration*[Title/Abstract]) OR "Patient Participation"[Mesh] OR (participat*[Title/Abstract]) OR (Patient involvement[Title/Abstract]) OR "Professional-Patient Relations"[Mesh] OR (Relationship*[Title/Abstract]) OR "Communication"[Mesh] OR (communication[Title/Abstract]) OR "Decision Making, Shared"[Mesh] OR (Patient decision making[Title/Abstract]) OR (Shared decision making[Title/Abstract]) OR (Empower*[Title/Abstract]) OR (Patient Activation[Title/Abstract]) OR "Patient Acceptance of Health Care"[Mesh] OR (Acceptance[Title/Abstract]) OR (self efficacy[Title/Abstract]) OR (self-efficacy[Title/Abstract]) OR "Self-Management"[Mesh] OR "Self Care"[Mesh] OR (Self manage*[Title/Abstract]) OR (Self-manage*[Title/Abstract]) OR (Self Care[Title/Abstract]) OR (Self-care[Title/Abstract]) OR (Commitment[Title/Abstract]) OR (Attitude[Title/Abstract]) OR (Engag*[Title/Abstract]) OR (Disengag*[Title/Abstract]) OR (Reengag*[Title/Abstract]) OR (Re-engag*[Title/Abstract]) OR (Return to Care[Title/Abstract]) OR (Restart[Title/Abstract]) OR (re-enter care[Title/Abstract]) OR (Trajectory[Title/Abstract]) OR (Journey[Title/Abstract]) OR (Disruption[Title/Abstract])) |
| AND | |
| Limit to sub-Saharan Africa | (Africa[tw] OR Africa[Mesh:noexp] OR (African[Title/Abstract])  OR Angola[tw] OR Benin[tw] OR Botswana[tw] OR Burkina Faso[tw] OR Burkina Fasso[tw] OR Upper Volta[tw] OR Burundi[tw] OR Urundi[tw] OR Cameroon[tw] OR Cameroons[tw] OR Cameron[tw] OR Camerons[tw] OR Cape Verde[tw] OR Central African Republic[tw] OR Chad[tw] OR Comoros[tw] OR Comoro Islands[tw] OR Comores[tw] OR Mayotte[tw] OR Congo[tw] OR Zaire[tw] OR Cote d'Ivoire[tw] OR Ivory Coast[tw] OR Djibouti[tw] OR French Somaliland[tw] OR Eritrea[tw] OR Ethiopia[tw] OR Gabon[tw] OR Gabonese Republic[tw] OR Gambia[tw] OR Ghana[tw] OR Gold Coast[tw] OR Guinea[tw] OR Lesotho[tw] OR Basutoland[tw] OR Liberia[tw] OR Madagascar[tw] OR Malagasy Republic[tw] OR Malawi[tw] OR Nyasaland[tw] OR Mali[tw] OR Mauritius[tw] OR Agalega Islands[tw] OR Mozambique[tw] OR Niger[tw] OR Nigeria[tw] OR Rwanda[tw] OR Ruanda[tw] OR Sao Tome[tw] OR Senegal[tw] OR Seychelles[tw] OR Sierra Leone[tw] OR Somalia[tw] OR (South Africa[Title/Abstract]) OR Sudan[tw] OR Swaziland[tw] OR Eswatini[tw] OR Togo[tw] OR Togolese Republic[tw] OR Uganda[tw] OR Zambia[tw] OR Zimbabwe[tw] OR Rhodesia[tw]  OR Angola[Mesh:noexp] OR Benin[Mesh:noexp] OR Botswana[Mesh:noexp] OR Cameroon[Mesh:noexp] OR Cape Verde[Mesh:noexp] OR Central African Republic[Mesh:noexp] OR Chad[Mesh:noexp] OR Comoros[Mesh:noexp] OR Congo[Mesh:noexp] OR Djibouti[Mesh:noexp] OR "Democratic Republic of the Congo"[Mesh:noexp] OR Eritrea[Mesh:noexp] OR Ethiopia[Mesh:noexp] OR Gabon[Mesh:noexp] OR Gambia[Mesh:noexp] OR Ghana[Mesh:noexp] OR Guinea[Mesh:noexp] OR Guinea‐Bissau[Mesh:noexp] OR Kenya[Mesh:noexp] OR Lesotho[Mesh:noexp] OR Liberia[Mesh:noexp] OR Madagascar[Mesh:noexp] OR Malawi[Mesh:noexp] OR Mali[Mesh:noexp] OR Mauritius[Mesh:noexp] OR Mozambique[Mesh:noexp] OR Namibia[Mesh:noexp] OR Niger[Mesh:noexp] OR Nigeria[Mesh:noexp] OR Rwanda[Mesh:noexp] OR Senegal[Mesh:noexp] OR Seychelles[Mesh:noexp] OR Sierra Leone[Mesh:noexp] OR Somalia[Mesh:noexp] OR South Africa[Mesh:noexp] OR Sudan[Mesh:noexp] OR Swaziland[Mesh:noexp] OR Tanzania[Mesh:noexp] OR Togo[Mesh:noexp] OR Uganda[Mesh:noexp] OR Zambia[Mesh:noexp] OR Zimbabwe[Mesh:noexp] |

#

## Table 2: Justification for the search parameters, search limits and eligibility criteria for inclusion of sources in the scoping study

| ITEM | Justification |
| --- | --- |
| SEARCH PARAMETERS AND LIMITS | |
| *Published in English* | The English limit brought feasibility to balance the breadth produced by the broad literature search (1). Restricting the search to ‘English’ reduced the volume of evidence by a small amount (e.g. by 0.6% for PubMed), which may reflect that English is main language for scientific publication (2). |
| *Published between the start of 2014 and when the search was conducted in February 2021* | Justification: The ART cohort and the HIV services both changed significantly in the early period after ART became accessible, and a 2014 threshold aligns with a period of more consistent guidance comparable to current recommendations.   - Firstly, the UNAIDS 90-90-90 cascade targets were introduced in 2014 (3). - Secondly, in the first decade of the 21^st^ century the focus for ART programmes in lower resource settings was on access and overcoming global reluctance to delivering ART in such settings, rather than on long-term engagement with care (4). Out of necessity, priority was initially given to prevention of mother to child transmission (PMTCT) and access for patients with advanced disease rather than considering comprehensive engagement (4), and scale up required adaptation of health systems to decentralise ART programmes and integrate them into their primary care services (5). - Thirdly, clinical guidelines also changed over this period, changing the dynamics of patient engagement with treatment. The World Health Organization (WHO) recommended the expansion of ART initiation to all people living with HIV (regardless of CD4 count) in November 2015 (6), significantly changing the patient population from those with more advanced disease who had specific motivations for engaging to a largely healthy population with different factors influencing their engagement (low CD4 marginally associated with decreased adherence (7)). This also changed measurement of engagement as there was no longer a need to differentiate the denominator of patients by eligibility (8). The introduction of newer medications such as tenofovir (introduced into the 2006 WHO guidelines and made first choice for first-line therapy in 2013 (9)) and dolutegravir (introduced in the 2015 WHO guidelines (6)), improved the tolerability of ART significantly (improving the pill burden and side effects). This reduced the impact of medication-factors on adherence and the stigma associated with ART (taking multiple tablets or stigmatising effects such as lipodystrophy) (10), and left health system and patient-factors with a greater proportion of the influence on engagement. |
| *Evidence from sub-Saharan African settings* | There are 30 countries are burdened with nearly 90% of the global HIV prevalence (3). The WHO African Region is most affected, accounting for two-thirds of new and current global HIV infections (68% of the global burden) (11). The largest ART programmes in lower resource settings are found in sub-Saharan African, which have particular challenges for patients engaging in care and considerations in measuring engagement (12-14).  While measures from other settings, both low and high income, could be valuable to inform measurement of engagement in sub-Saharan Africa, to manage the scope of the review this study prioritised measures used in sub-Saharan Africa (drawing from search terms developed by Visser et al (15) for lower income countries) in order to scope evidence on measuring engagement with HIV care that reflects context-specific dynamics of engagement and are feasible in these settings. Sources from multi-country cohorts that included countries from sub-Saharan Africa were also included. |
| ELIGIBILITY | |
| *Patients on lifelong art or who have initiated art previously (includes PMTCT option B+)* | Engagement with initiation and maintenance of treatment are conceptually different behaviours (16), with different denominators for evaluation. The majority of people living with HIV know their status and have initiated treatment at some point (17), making maintenance of engagement with ART a priority. |
| *Adults ≥18 years old, including young adults and the elderly* | The study included evidence on measuring engagement in adults (including both young adults and aging populations) as the dynamics of engagement are somewhat particular in paediatric and adolescent populations (18). |

###

### References for Table 2

1. Levac, D., Colquhoun, H., & O’Brien, K. K. (2010). Scoping studies: Advancing the methodology. *Implementation Science*, *5*(1), 1–9. <https://doi.org/10.1186/1748-5908-5-69>
2. Kamadjeu, R. (2019). English: the lingua franca of scientific research. *The Lancet Global Health*, *7*(9), e1174. https://doi.org/10.1016/S2214-109X(19)30258-X
3. UNAIDS. (2014). *Fast-track: ending the AIDS epidemic by 2030*.
4. Osler, M., Hilderbrand, K., Goemaere, E., Ford, N., Smith, M., Meintjes, G., Kruger, J., Govender, N. P., & Boulle, A. (2018). The Continuing Burden of Advanced HIV Disease over 10 Years of Increasing Antiretroviral Therapy Coverage in South Africa. *Clinical Infectious Diseases*, *66*(Figure 1), S118–S125. https://doi.org/10.1093/cid/cix1140
5. Ford, N., Calmy, A., & Mills, E. J. (2011). The first decade of antiretroviral therapy in Africa. *Globalization and Health*, *7*, 1–6. https://doi.org/10.1186/1744-8603-7-33
6. World Health Organization. (2015). Policy Brief: Consolidated guidelines on the use of antiretroviral therapy and preventing HIV infection. What’s new. In *WHO Guidelines* (Issue 2). www.who.int/about/licensing/copyright_form/en/index.html
7. Bock, P., James, A., Nikuze, A., Peton, N., Sabapathy, K., Mills, E., Fidler, S., & Ford, N. (2016). Baseline CD4 count and adherence to antiretroviral therapy: A systematic review and meta-analysis. *Journal of Acquired Immune Deficiency Syndromes*, *73*(5), 514–521. <https://doi.org/10.1097/QAI.0000000000001092>
8. Rollins, N. C., Becquet, R., Orne-Gliemann, J., Phiri, S., Hayashi, C., Baller, A., & Shaffer, N. (2014). Defining and analyzing retention-in-care among pregnant and breastfeeding HIV-infected women: Unpacking the data to interpret and improve PMTCT outcomes. *Journal of Acquired Immune Deficiency Syndromes*, *67*, S150–S156. https://doi.org/10.1097/QAI.0000000000000355
9. World Health Organization. (2013). *Consolidated guidelines on the use of antiretroviral drugs for treating and preventing HIV infection* (Issue June).
10. Hirasen, K., Evans, D., Maskew, M., Sanne, I. M., Shearer, K., Govathson, C., Malete, G., Kluberg, S. A., & Fox, M. P. (2018). The right combination – treatment outcomes among HIV-positive patients initiating first-line fixed-dose antiretroviral therapy in a public sector HIV clinic in johannesburg, South Africa. *Clinical Epidemiology*, *10*, 17–29. <https://doi.org/10.2147/CLEP.S145983>
11. World Health Organization. (2020). *WHO Africa: HIV/AIDS*. <https://www.afro.who.int/health-topics/hivaids>. Date accessed: 12-02-2021
12. Hajizadeh, M., Sia, D., Heymann, S. J., & Nandi, A. (2014). Socioeconomic inequalities in HIV/AIDS prevalence in sub-Saharan African countries: Evidence from the Demographic Health Surveys. *International Journal for Equity in Health*, *13*(1). https://doi.org/10.1186/1475-9276-13-18
13. Nyindo, M. (2005). Complementary factors contributing to the rapid spread of HIV-I in sub-Saharan Africa: a review. *East African Medical Journal*, *82*(1), 40–46. https://doi.org/10.4314/eamj.v82i1.9293
14. Tsafack Temah, C. (2009). What Drives HIV/AIDS Epidemic in Sub-Saharan Africa? *Revue d’économie du développement*, *17*(5), 41–70. https://doi.org/10.3917/edd.235.0041
15. Visser, M. E., Schoonees, A., Ezekiel, C. N., Randall, N. P., & Naude, C. E. (2020). Agricultural and nutritional education interventions for reducing aflatoxin exposure to improve infant and child growth in low- and middle-income countries. *Cochrane Database of Systematic Reviews*, *4*, Art. No.: CD013376. https://doi.org/10.1002/14651858.CD013376
16. Amico, R. K. (2011). A situated-information motivation behavioral skills model of care initiation and maintenance (sIMB-CIM): An IMB model based approach to understanding and intervening in engagement in care for chronic medical conditions. *Journal of Health Psychology*, *16*(7), 1071–1081. https://doi.org/10.1177/1359105311398727
17. Marsh, K., Eaton, J. W., Mahy, M., Sabin, K., Autenrieth, C. S., Wanyeki, I., Daher, J., & Ghys, P. D. (2019). Global, regional and country-level 90–90–90 estimates for 2018 assessing progress towards the 2020 target. *AIDS*, *33*, S213–S226. https://doi.org/10.1097/QAD.0000000000002355
18. Crowley, T., van der Merwe, A., Kidd, M., & Skinner, D. (2020). Measuring Adolescent HIV Self-management: An Instrument Development Study. *AIDS and Behavior*, *24*(2), 592–606. https://doi.org/10.1007/s10461-019-02490-z

## Table 3: Data extracted from the included sources identified in the search

| Information on the study | Information on the measures of engagement |
| --- | --- |
| - Year of publication - Countries participants were from - Setting (e.g. Hospital based vs primary care-based outpatient services) - Participants   - Age of participants   - Gender of participants   - First or second line regimens included - Methods   - Date range of study   - Study type   - Analysis methods   - Focus on the evaluation of the measure or not - Key findings | - Element of engagement measured - Measure of engagement - Information on the measure   - Definition of measure   - Data collected   - Feasibility of collection   - Data source   - Observation time   - Calculation   - Categorisation   - Interpretation   - Assumptions and definitions required to understand the measure - Evaluation of the measure   - Development or validation of the measure   - Association with outcomes   - Association with other measures of engagement   - Considerations, strengths and limitations - How the measures were used in the included source or how the literature recommended the measure could be used |

## Table 4: List of included sources

| Reference | Year | Country | Study type | Metric focus | Main element of engagement measured | Measures of engagement | Use cases |
| --- | --- | --- | --- | --- | --- | --- | --- |
| Main search | | | | | | |  |
| Published literature | | | | | | |  |
| Abah IO, Ojeh VB, Musa J, et al. Clinical Utility of Pharmacy-Based Adherence Measurement in Predicting Virologic Outcomes in an Adult HIV-Infected Cohort in Jos, North Central Nigeria. J Int Assoc Provid AIDS Care. 2016;15(1):77-83. doi:10.1177/2325957414539197 | 2016 | Nigeria | Retrospective cohort | Main focus | Adherence | Percentage cumulative adherence to drug refill visits | Individual patient evaluation |
| Abdulrahman SA, Ganasegeran K, Rampal L, Martins OF. HIV Treatment Adherence - A Shared Burden for Patients, Health-Care Providers, and Other Stakeholders. AIDS Rev. 2019;21(1):28-39. doi:10.24875/AIDSRev.19000037 | 2019 | Global: Europe, Africa, Asia | Systematic review | Not primary purpose but evaluates partially | Adherence | 1. Patient self reports 2. Pill counts (facility-based) 3. Serum assay of drug levels 4. Electronic pill caps | Routine monitoring and evaluation of programmes at facility and population levels for quality improvement and strategic decision-making  Understanding patient behaviour |
| Adefolalu A, Nkosi Z, Olorunju S, Masemola P. Self-efficacy, medication beliefs and adherence to antiretroviral therapy by patients attending a health facility in Pretoria. South African Fam Pract. 2014;56(5):281-285. doi:10.1080/20786190.2014.975476 | 2014 | South Africa (Pretoria) | Cross sectional | Main focus | Other | 1. HIV adherence self efficacy scale (HIV-ASES) 2. Beliefs about medicines questionnaire (BMQ) 2a. BMQS (specific) 2b. BMQG (general) 3. AIDS Clinical Trial Group Questionnaire (ACTG) | Understanding patient behaviour  Identification of factors to intervene on |
| Agaba PA, Genberg BL, Sagay AS, et al. Retention in Differentiated Care: Multiple Measures Analysis for a Decentralized HIV Care and Treatment Program in North Central Nigeria. J AIDS Clin Res. 2018;9(2). doi:10.4172/2155-6113.1000756 | 2018 | Nigeria | Retrospective cohort | Not primary purpose but evaluates partially | Retention | 1. Summary patient-level measures 1a. Retention at fixed time points (fixed RIC) 1b. Cumulative loss to follow up (LTFU)  2. Consistency with visit-level measures 2a. Gap in care 2b. Visit constancy | Routine monitoring and evaluation of programmes at facility and population levels for quality improvement and strategic decision-making  Routine evaluation of engagement in patients in differentiated service delivery models |
| Agala CB, Fried BJ, Thomas JC, et al. Reliability, validity and measurement invariance of the Simplified Medication Adherence Questionnaire (SMAQ) among HIV-positive women in Ethiopia: a quasi-experimental study. BMC Public Health. 2020;20(1):567. doi:10.1186/s12889-020-08585-w | 2020 | Ethiopia | Cross sectional | Main focus | Adherence | Simplified Medication Adherence Questionnaire (SMAQ) | Development and evaluation of interventions |
| Ahoua L, Arikawa S, Tiendrebeogo T, et al. Measuring retention in care for HIV-positive pregnant women in Prevention of Mother-to-Child Transmission of HIV (PMTCT) option B+ programs: the Mozambique experience. BMC Public Health. 2020;20(1):322. doi:10.1186/s12889-020-8406-5 | 2020 | Mozambique | Retrospective cohort | Main focus | Retention | Retention - single time point 1. Point retention 2. MoH definition 3. WHO definition - continuous engagement along the continuum of care 4. Inter-agency Task Team (IATT) definition 5. Appointment adherence retention  6. On time adherence retention (reference) | Routine evaluation of engagement in patients in facility care |
| Alcaide ML, Ramlagan S, Rodriguez VJ, et al. Self-Report and Dry Blood Spot Measurement of Antiretroviral Medications as Markers of Adherence in Pregnant Women in Rural South Africa. AIDS Behav. 2017;21(7):2135-2140. doi:10.1007/s10461-017-1760-3 | 2017 | South Africa | Prospective cohort | Main focus | Adherence | 1. Self report  1a. Visual Analog Scale (VAS) 1b. AIDS Clinical Trials Group Adherence (ACTG) 2. Dried blood spot (DBS) for ARV concentration (TDF, 3TC, EFV) used as the gold standard 3. Male involvement index | Understanding patient behaviour  Identification of factors to intervene on |
| Angwenyi V, Bunders-Aelen J, Criel B, Lazarus J V, Aantjes C. An evaluation of self-management outcomes among chronic care patients in community home-based care programmes in rural Malawi: A 12-month follow-up study. Health Soc Care Community. Published online July 2020. doi:10.1111/hsc.13094 | 2021 | Malawi | Non randomised interventional | Means to an end | Other | Self management OUTCOME 1. Self-efficacy 2. Health status 2a. Perceived illness intrusiveness 2b. Patient symptom rating 2c. Self-rated general health  2d. Quality of life | Development and evaluation of interventions |
| Anoje C, Agu KA, Oladele EA, et al. Adherence to On-Time ART Drug Pick-Up and Its Association with CD4 Changes and Clinical Outcomes Amongst HIV Infected Adults on First-Line Antiretroviral Therapy in Nigerian Hospitals. AIDS Behav. 2017;21(2):386-392. doi:10.1007/s10461-016-1473-z | 2017 | Nigeria | Retrospective cohort | Main focus | Retention | 1. On time ART refill  Comparisons 2. CD4 3. Ois | Routine evaluation of engagement in patients in facility care |
| Aregbesola OH, Adeoye IA. Self-efficacy and antiretroviral therapy adherence among HIV positive pregnant women in South-West Nigeria: a mixed methods study. Tanzan J Health Res. 2018;20(4):unpaginated. https://www.ajol.info/index.php/thrb/article/view/170709/166340 | 2018 | Nigeria | Mixed Methods | Means to an end | Other | 1. Treatment self efficacy  Comparison 2. Self-reported adherence | Routine monitoring and evaluation of programmes at facility and population levels for quality improvement and strategic decision-making  Understanding patient behaviour  Identification of factors to intervene on |
| Areri H, Marshall A, Harvey G. Factors influencing self-management of adults living with HIV on antiretroviral therapy in Northwest Ethiopia: a cross-sectional study. BMC Infect Dis. 2020;20(1):879. doi:10.1186/s12879-020-05618-y | 2020 | Ethiopia | Cross sectional | Means to an end | Other | Self management OUTCOME a. Daily physical health practice b. Activating social support c. Living with chronic HIV  Evaluation of factors that may affect self-managmeent 1. Self-management PROCESS 1a. ART knowledge 1b. Self-efficacy 1c. Self-regulation 1d. Social facilitation  2. Self-management INTERVENTIONS  3. Patient characteristics | Understanding patient behaviour  Identification of factors to intervene on |
| Atanga PN, Ndetan HT, Fon PN, et al. Using a composite adherence tool to assess ART response and risk factors of poor adherence in pregnant and breastfeeding HIV-positive Cameroonian women at 6 and 12 months after initiating option B. BMC Pregnancy Childbirth. 2018;18(1):418. doi:10.1186/s12884-018-2058-9 | 2018 | Cameroon | Prospective cohort | Main focus | Adherence | Composite Adherence Score (CAS) | Support ART management decisions (through initiating conversations with patients or providing healthcare workers with information)  Identification of factors to intervene on |
| Banas K, Lyimo RA, Hospers HJ, van der Ven A, de Bruin M. Predicting adherence to combination antiretroviral therapy for HIV in Tanzania: A test of an extended theory of planned behaviour model. Psychol Health. 2017;32(10):1249-1265. doi:10.1080/08870446.2017.1283037 | 2017 | Tanzania | Prospective cohort | Not primary purpose but evaluates partially | Other | 1. Motivation factors 1a. Attitude 1b. Subjective norm 1c. Perceived behavioural control (PBC) 1d. Anticipated regret 2. Adherence intention 3. Intention-behaviour mediators 3a. Self-regulation 3b. Action planning  Comparison 4. Adherence by MEMS | Identification of factors to intervene on |
| Beres LK, Schwartz S, Simbeza S, et al. Patterns and Predictors of Incident Return to HIV Care Among Traced, Disengaged Patients in Zambia: Analysis of a Prospective Cohort. J Acquir Immune Defic Syndr. 2021;86(3):313-322. doi:10.1097/QAI.0000000000002554 | 2021 | Zambia | Prospective cohort | Means to an end | Retention | 1. Re-engagement 1a. Proportion re-engaged 1b. Time to re-engagement | Understanding patient behaviour |
| Boussari O, Subtil F, Genolini C, et al. Impact of variability in adherence to HIV antiretroviral therapy on the immunovirological response and mortality Data analysis, statistics and modelling. BMC Med Res Methodol. 2015;15(1). doi:10.1186/1471-2288-15-10 | 2015 | Senegal | Secondary analysis | Main focus | Adherence | 1. Pattern of adherence 1a. Pattern of average adherence 1b. Pattern of adherence variance  Comparisons 2. VL 3. CD4 4. Mortality | Understanding patient behaviour |
| Buzibye A, Musaazi J, von Braun A, et al. Antiretroviral concentration measurements as an additional tool to manage virologic failure in resource limited settings: a case control study. AIDS Res Ther. 2019;16(1):39. doi:10.1186/s12981-019-0255-x | 2019 | Uganda | Case control | Main focus | Adherence | 1. Plasma ARV drug concentration 1a. Efavirenz 1b. Nevirapine 1c. Lopinavir 1d. Atazanavir  Comparisons 2. VL | Support ART management decisions (through initiating conversations with patients or providing healthcare workers with information) |
| Byabene AK, Fortes-Déguénonvo L, Niang K, et al. Optimal antiretroviral therapy adherence as evaluated by CASE index score tool is associated with virological suppression in HIV-infected adults in Dakar, Senegal. Trop Med Int Health. 2017;22(6):776-782. doi:10.1111/tmi.12882 | 2017 | Senegal | Cross sectional | Main focus | Adherence | 1. Center for Adherence Support Evaluation (CASE) Index Score  Comparison 2. VL | Understanding patient behaviour |
| Chawana TD, Gandhi M, Nathoo K, et al. Defining a Cutoff for Atazanavir in Hair Samples Associated With Virological Failure Among Adolescents Failing Second-Line Antiretroviral Treatment. J Acquir Immune Defic Syndr. 2017;76(1):55-59. doi:10.1097/QAI.0000000000001452 | 2017 | Zimbabwe | Secondary analysis | Main focus | Adherence | 1. Hair ARV concentration: Atazanavir  Comparisons 2. Self report 3. VL | Development and evaluation of interventions |
| Chawana TD, Nhachi CFB, Nathoo K, et al. Higher tenofovir concentrations in hair are associated with decreases in viral load and not self-reported adherence in HIV-infected adolescents with second line virological treatment failure. AIDS Res Hum Retroviruses. Published online January 2021. doi:10.1089/AID.2020.0258 | 2021 | Zimbabwe | Secondary analysis | Main focus | Adherence | 1. Hair ARV concentration: Tenofovir  Comparisons 2. Self report 3. VL | Support ART management decisions (through initiating conversations with patients or providing healthcare workers with information) |
| Chidrawi HC, Greeff M, Temane QM, Ellis S. Changeover-time in psychosocial wellbeing of people living with HIV and people living close to them after an HIV stigma reduction and wellness enhancement community intervention. Afr J AIDS Res. 2015;14(1):1-12. doi:10.2989/16085906.2014.961940 | 2015 | South Africa | Prospective cohort | Means to an end | Other | Coping self-efficacy | Development and evaluation of interventions |
| Chidrawi HC, Greeff M, Temane QM. Health behaviour change of people living with HIV after a comprehensive community-based HIV stigma reduction intervention in North-West Province in South Africa. SAHARA J J Soc Asp HIV/AIDS Res Alliance. 2014;11(1):222-232. doi:10.1080/17290376.2014.985700 | 2014 | South Africa | Prospective cohort | Means to an end | Other | Measured health behaviour through 1. Self reported adherence 2. Self-care Symptom Management  Comparison (outcome) 3. HIV/AIDS targeted QoL | Development and evaluation of interventions |
| Corless IB, Hoyt AJ, Tyer-Viola L, et al. 90-90-90-Plus: Maintaining Adherence to Antiretroviral Therapies. AIDS Patient Care STDs. 2017;31(5):227-236. doi:10.1089/apc.2017.0009 | 2017 | Canada, Namibia, Thailand, United States, Puerto Rico | Cross sectional | Means to an end | Other | 1. Engagement with healthcare provider 2. Self efficacy 2a. HIV adherence self efficacy 2b. Chronic disease self efficacy  Comparison 3. Self reported adherence by VAS | Support ART management decisions (through initiating conversations with patients or providing healthcare workers with information) |
| Court R, Leisegang R, Stewart A, et al. Short term adherence tool predicts failure on second line protease inhibitor-based antiretroviral therapy: an observational cohort study. BMC Infect Dis. 2014;14:664. doi:10.1186/s12879-014-0664-3 | 2014 | South Africa | Retrospective cohort | Main focus | Adherence | Short term pharmacy refill adherence 1. Interval gap 2. Interval average (MPR) 3. Interval crude (pharmacy visit compliance)  Long term pharmacy refill adherence | Rationalise resources (such as expensive genotypes) |
| Crowley T, Van der Merwe A, Kidd M, Skinner D. Measuring Adolescent HIV Self-management: An Instrument Development Study. AIDS Behav. 2020;24(2):592-606. doi:10.1007/s10461-019-02490-z | 2020 | South Africa | Mixed Methods | Main focus | Other | 1. Adolescent HIV Self-management scale (AdHIVSM scale)  Comparisons 2. Process 2a. Treatment self efficacy (ASES) 2b. Resilience (CYRM-12) 3. Proximal outcomes 3a. Treatment adherence 3b. Retention (clinic attendance 3c. Sexual behaviour and substance use 4. Distal outcome 4a. VL 4b. QoL | Individual patient evaluation  Understanding patient behaviour |
| Crowley, T., van der Merwe, A., et al. (2020) ‘Adolescent human immunodeficiency virus self-management: Associations with treatment adherence, viral suppression, sexual risk behaviours and health-related quality of life’, Southern African Journal of HIV Medicine, 21(1), pp. 1–11. doi: 10.4102/SAJHIVMED.V21I1.1054. | 2020 | South Africa | Cross sectional | Main focus | Other | 1. Self-management  Comparisons 2. Adherence 3. VL 4. Health related QoL 5. Sexual risk behaviours | Understanding patient behaviour |
| Czaicki NL, Dow WH, Njau PF, McCoy SI. Do incentives undermine intrinsic motivation? Increases in intrinsic motivation within an incentive-based intervention for people living with HIV in Tanzania. PLoS One. 2018;13(6):e0196616. doi:10.1371/journal.pone.0196616 | 2018 | Tanzania | Secondary analysis | Means to an end | Other | Intrinsic motivation | Identification of factors to intervene on  Development and evaluation of interventions |
| Denison JA, Koole O, Tsui S, et al. Incomplete adherence among treatment-experienced adults on antiretroviral therapy in Tanzania, Uganda and Zambia. AIDS. 2015;29(3):361-371. doi:10.1097/QAD.0000000000000543 | 2015 | Tanzania, Uganda and Zambia | Cross sectional | Dual focus(main and applied to a question) | Adherence | 1. Self reported adherence 1a. 3 day recall (AACTG) 1b. 30 day recall 1c. CASE Index  1d. 30 day VAS 1e. ≥48 consecutive hours of missed ART 2. Healthcare provider adherence estimates 3. Pharmacy-based medication possession ratio  Comparison 4. VL | Routine monitoring and evaluation of programmes at facility and population levels for quality improvement and strategic decision-making |
| Denison JA, Packer C, Stalter RM, et al. Factors Related to Incomplete Adherence to Antiretroviral Therapy among Adolescents Attending Three HIV Clinics in the Copperbelt, Zambia. AIDS Behav. 2018;22(3):996-1005. doi:10.1007/s10461-017-1944-x | 2018 | Zambia | Cross sectional | Not primary purpose but evaluates partially | Other | 1. Self-management  Comparison 2.Adherence 2a. ≥48 hour treatment interruption 2b. 6 month MPR | Understanding patient behaviour  Identification of factors to intervene on |
| Dewing S, Mathews C, Lurie M, Kagee A, Padayachee T, Lombard C. Predictors of poor adherence among people on antiretroviral treatment in Cape Town, South Africa: a case-control study. AIDS Care. 2015;27(3):342-349. doi:10.1080/09540121.2014.994471 | 2015 | South Africa | Case control | Not primary purpose but evaluates partially | Other | 1. IMB Skills ART Adherence 1a. Information 1b. Motivation 1c. Behaviour skills  Comparison 2. Adherence defined by pill count or late for visit | Understanding patient behaviour  Identification of factors to intervene on |
| Dodzo LG, Mahaka HT, Mukona D, Zvinavashe M, Haruzivishe C. HIV self-care practices during pregnancy and maternal health outcomes among HIV-positive postnatal mothers aged 18-35 years at Mbuya Nehanda maternity hospital. AIDS Care. 2017;29(6):741-745. doi:10.1080/09540121.2016.1242710 | 2017 | Zimbabwe | Cross sectional | Not primary purpose but evaluates partially | Other | 1. Self care practices  Comparison 2. Maternal outcome | Understanding patient behaviour |
| Drain P, National Institute of Allergy and Infectious Diseases (NIAID), Centre for the AIDS Programme of Research in South Africa. Simplifying Treatment and Monitoring for HIV. ClinicalTrials.gov. NCT04341779 | 2020 | South Africa | RCT | Dual focus(main and applied to a question) | Other | 1. Intervention: POC 1a. Tenofovir diphosphate 1b. VL  Comparison (primary outcomes) 2. Adherence by ARV drug concentration 3. Composite measure of engagement 3a. Virological suppression 3b. Retention | Routine evaluation of engagement in patients in facility care |
| Eby J, Chapman J, Marukutira T, et al. The adherence-outcome relationship is not altered by diary-driven adjustments of microelectronic monitor data. Pharmacoepidemiol Drug Saf. 2015;24(12):1313-1320. doi:10.1002/pds.3887 | 2015 | Botswana | Prospective cohort | Main focus | Adherence | 1. MEMs data: Medication Event Monitoring System (MEMS) 1.1. Raw MEMS data Modifications: 1.2. Diary-cleaned MEMS data 1.3. Capped MEMS data 1.4. Capped and diary-cleaned MEMS data  Comparison 2. VL | Understanding patient behaviour |
| Erb S, Letang E, Glass TR, et al. A simple visual analog scale is a valuable tool to assess self-reported adherence in HIV-infected patients on antiretroviral treatment in a resource-limited setting. J AIDS Clin Res. 2017;8(9):731. doi:http://dx.doi.org/10.4172/2155-6113.1000731 | 2017 | Tanzania | Secondary analysis | Main focus | Adherence | 1. pictogram-enhanced visual analogue scale (peVAS)  Comparison 2. Standardised self-report questionnaire 3. ARV concentration 4. VL | Individual patient evaluation |
| Esber A, Polyak C, Kiweewa F, et al. Persistent Low-level Viremia Predicts Subsequent Virologic Failure: Is It Time to Change the Third 90? Clin Infect Dis an Off Publ Infect Dis Soc Am. 2019;69(5):805-812. doi:10.1093/cid/ciy989 | 2018 | Uganda, Kenya, tanzania, Nigeria | Prospective cohort | Means to an end | Adherence | 1. Persistant low level viraemia  Comparison 2. Viral failure 3.Self-reported adherence | Flag patients early for intervention |
| Fedlu A, Alie B, Siraj Mohammed A, Adem F, Hassen A. Adherence to Antiretroviral Treatment for Prevention of Mother-to-Child Transmission of HIV in Eastern Ethiopia: A Cross-Sectional Study. HIV AIDS (Auckl). 2020;12:725-733. doi:10.2147/HIV.S274012 | 2020 | Ethiopia | Cross sectional | Means to an end | Adherence | Adherence | Routine monitoring and evaluation of programmes at facility and population levels for quality improvement and strategic decision-making |
| Font H, Rollins N, Essajee S, et al. Retention-in-care in the PMTCT cascade: definitions matter! Analyses from the INSPIRE projects in Malawi, Nigeria and Zimbabwe. J Int AIDS Soc. 2020;23(10):e25609. doi:10.1002/jia2.25609 | 2020 | Malawi, Nigeria, Zimbabwe | Secondary analysis | Main focus | Retention | Based on missed visits 1. Missed visits - 14 days 2. Missed visits - 30 days 3. Missed visits - 60 days 4. <25% missed visits  5. Attendence at a final visit  6. Number clinic visits 7. Visit constancy 8. Gaps in care 9. Number of isolated clinic visits | Routine monitoring and evaluation of programmes at facility and population levels for quality improvement and strategic decision-making |
| Fox M., Bor J, Brennan AT, et al. Estimating retention in HIV care accounting for patient transfers: A national laboratory cohort study in South Africa. PLoS Med. 2018;15(6):e1002589. doi:http://dx.doi.org/10.1371/journal.pmed.1002589 | 2018 | South Africa | Retrospective cohort | Means to an end | Retention | 1. Retention at clinic level  1a. Point estimate retention at end of follow up time  1b. Continually retained  2. Retention at national level  2a. Point estimate retention at end of follow up time 2b. Continually retained | Routine monitoring and evaluation of programmes at facility and population levels for quality improvement and strategic decision-making |
| Friedland BA, Gottert A, Hows J, et al. The People Living with HIV Stigma Index 2.0: generating critical evidence for change worldwide. AIDS. 2020;34 Suppl 1:S5-S18. doi:10.1097/QAD.0000000000002602 | 2020 | Senegal, Cameroon, Uganda | Cross sectional | Main focus | Other | 1. Internalized stigma and resilience 2. Interactions with healthcare services | Development and evaluation of interventions |
| Gachara G, Mavhandu LG, Rogawski ET, Manhaeve C, Bessong PO. Evaluating Adherence to Antiretroviral Therapy Using Pharmacy Refill Records in a Rural Treatment Site in South Africa. AIDS Res Treat. 2017;2017:5456219. doi:10.1155/2017/5456219 | 2017 | South Africa | Cross sectional | Not primary purpose but evaluates partially | Adherence | 1. Pharmacy refill adherence  Comparison 2. Viral failure | Routine monitoring and evaluation of programmes at facility and population levels for quality improvement and strategic decision-making |
| Genn L, Chapman J, Okatch H, et al. Pharmacy Refill Data are Poor Predictors of Virologic Treatment Outcomes in Adolescents with HIV in Botswana. AIDS Behav. 2019;23(8):2130-2137. doi:10.1007/s10461-018-2325-9 | 2019 | Botswana | Prospective cohort | Main focus | Adherence | Pharmacy refill adherence 1. Binary outcome 2. Continuous outcome  3. Electronic medication adherence  4. CD4 as proxy  5. Medication autonomy | Flag patients early for intervention |
| George L, Muro EP, Ndaro A, Dolmans W, Burger DM, Kisanga ER. Nevirapine concentrations in saliva measured by thin layer chromatography and self-reported adherence in patients on antiretroviral therapy at Kilimanjaro Christian Medical Centre, Tanzania. *Ther Drug Monit*. 2014;36(3):366-370. doi:10.1097/FTD.0000000000000005 | 2014 | Tanzania | Cross sectional | Main focus | Adherence | 1. ARV drug concentration in saliva (nevirapine)  Comparisons 2. Self-reported adherence | Individual patient evaluation |
| Grabowski MK, Reynolds SJ, Kagaayi J, et al. The validity of self-reported antiretroviral use in persons living with HIV: A population-based study. AIDS. 2018;32(3):363-369. doi:10.1097/QAD.0000000000001706 | 2018 | Uganda | Cross sectional | Main focus | Other | 1. Self reported ART use | Routine monitoring and evaluation of programmes at facility and population levels for quality improvement and strategic decision-making |
| Guira O, Kaboré DSRR, Dao G, et al. The Modalities of Nonadherence to Highly Active Antiretroviral Therapy and the Associated Factors Related to Patients’ Sociodemographic Characteristics and Their Caregiving Perceptions in Ouagadougou (Burkina Faso). J Int Assoc Provid AIDS Care. 2016;15(3):256-260. doi:10.1177/2325957415616492 | 2016 | Burkina Faso | Cross sectional | Means to an end | Adherence | 1. Global adherence 2. Quantitative adherence 3. Qualitative adherence | Understanding patient behaviour  Identification of factors to intervene on |
| Harris RA, Haberer JE, Musinguzi N, et al. Predicting short-term interruptions of antiretroviral therapy from summary adherence data: Development and test of a probability model. PLoS One. 2018;13(3):e0194713. doi:10.1371/journal.pone.0194713 | 2018 | Uganda | Modelling | Main focus | Adherence | 1. Treatment interruption prediction score  Comparisons 2. Treatment gap - measure of retention | Flag patients early for intervention |
| Henegar CE, Westreich D, Maskew M, et al. Comparison of pharmacy-based measures of adherence to antiretroviral therapy as predictors of virological failure. AIDS Behav. 2015;19(4):612-618. doi:10.1007/s10461-014-0953-2 | 2015 | South Africa | Prospective cohort | Main focus | Combination | Pharmacy measures 1. Pickup timing 1a. On time/ late pickup if late: 1b. Relative to median lateness 1c. 30 day lateness  2. Pill coverage 2a. 100% coverage 2b. ≥90% coverage 2c. ≥80% coverage  3. Composite pharmacy refill metric of attendance and coverage  4. Self report  Comparison  5. VL | Support ART management decisions (through initiating conversations with patients or providing healthcare workers with information) |
| Hermans LE, Steegen K, Ter Heine R, et al. Drug level testing as a strategy to determine eligibility for drug resistance testing after failure of ART: a retrospective analysis of South African adult patients on second-line ART. J Int AIDS Soc. 2020;23(6):e25501. doi:10.1002/jia2.25501 | 2020 | South Africa | Retrospective cohort | Main focus | Adherence | 1. Qualitative ART exposure  Comparison 2. ARV resistance | Rationalise resources (such as expensive genotypes) |
| Hickey MD, Salmen CR, Tessler RA, et al. Antiretroviral concentrations in small hair samples as a feasible marker of adherence in rural Kenya. J Acquir Immune Defic Syndr. 2014;66(3):311-315. doi:10.1097/QAI.0000000000000154 | 2014 | Kenya | Prospective cohort | Main focus | Adherence | 1. Nevirapine concentration from 1cm hair  2. Self report via the AIDS Clinical Trials Group (ACTG) 4 day adherence recall questionnaire | Rationalise resources (such as expensive genotypes) |
| Hine P, Smith R, Eshun‐Wilson I, et al. Measures of antiretroviral adherence for detecting viral non‐suppression in people living with HIV. Cochrane Database Syst Rev. 2018;(7). doi:10.1002/14651858.CD013080 | 2018 | global | Systematic review | Main focus | Adherence | Simple measures of adherence 1. Patient self‐report 2. Tablet counts 3. Pharmacy records 4. Electronic monitoring 5. Composite methods  Comparisons 6. Non‐suppressed viral load | Rationalise resources (such as viral load testing) |
| Hirasen K, Evans D, Jinga N, et al. Using a Self-Administered Electronic Adherence Questionnaire to Identify Poor Adherence Amongst Adolescents and Young Adults on First-Line Antiretroviral Therapy in Johannesburg, South Africa. Patient Prefer Adherence. 2020;14:133-151. doi:10.2147/PPA.S210404 | 2020 | South Africa | Cross sectional | Main focus | Adherence | 1. Electronic self-administered self report 2. Paperbased interviewer administered self report  3. Simplified Medication Adherence Questionnaire (SMAQ) 4. South African NDoH Adherence Questionnaire  Reference adherence 5. ARV drug level in plasma: efavirenz 6. VL | Flag patients early for intervention |
| Jackson IL, Umoh SS, Erah PO. Medication Adherence and Health Status in HIV Positive Patients in Akwa Ibom State, Nigeria. Trop J Pharm Res. 2020;19(10):2197-2204. doi:10.4314/tjpr.v19i10.25 | 2020 | Nigeria | Cross sectional | Means to an end | Adherence | 1. Self reported Adherence to Refills and Medication Scale (ARMS-7)  Comparison 2. Self reported health status | Routine monitoring and evaluation of programmes at facility and population levels for quality improvement and strategic decision-making  Routine evaluation of engagement in patients in facility care |
| John ME, Samson-Akpan PE, Etowa JB, Akpabio II, John EE. Enhancing self-care, adjustment and engagement through mobile phones in youth with HIV. Int Nurs Rev. 2016;63(4):555-561. doi:10.1111/inr.12313 | 2016 | Nigeria | Non randomised interventional | Means to an end | Other | 1. Patient activation 2. Self-care | Development and evaluation of interventions |
| Johnson LF, Estill J, Keiser O, et al. Do Increasing Rates of Loss to Follow-up in Antiretroviral Treatment Programs Imply Deteriorating Patient Retention? Am J Epidemiol. 2014;180(12):1208-1212. doi:10.1093/aje/kwu295 | 2014 | South Africa | Modelling | Main focus | Retention | 1. Retrospective definition of LTFU 2. Prospective definition of LTFU | Routine monitoring and evaluation of programmes at facility and population levels for quality improvement and strategic decision-making |
| Johnston J, Wiesner L, Smith P, Maartens G, Catherine Orrell. Correlation of hair and plasma efavirenz concentrations in HIV-positive South Africans. South Afr J HIV Med. 2019;20(1):1-6. doi:10.4102/sajhivmed.v20i1.881 | 2019 | South Africa | Secondary analysis | Main focus | Adherence | 1. Hair ARV concentration 2. Plasma ARV correlation  Comparisons 3. Adherence by electronic device 4. Viral load | Support ART management decisions (through initiating conversations with patients or providing healthcare workers with information) |
| Kapiamba G, Masango T, Mphuthi D. Antiretroviral adherence and virological outcomes in HIV-positive patients in Ugu district, KwaZulu-Natal province. Afr J AIDS Res. 2016;15(3):195-201. doi:10.2989/16085906.2016.1170710 | 2016 | South Africa | Retrospective cohort | Dual focus(main and applied to a question) | Adherence | 1. Pharmacy refill  Comparisons 2. VL | Routine monitoring and evaluation of programmes at facility and population levels for quality improvement and strategic decision-making  Routine evaluation of engagement in patients in facility care |
| Kekwaletswe CT, Morojele NK. Patterns and predictors of antiretroviral therapy use among alcohol drinkers at HIV clinics in Tshwane, South Africa. AIDS Care. 2014;26 Suppl 1:S78-82. doi:10.1080/09540121.2014.906558 | 2014 | South Africa | Prospective cohort | Means to an end | Adherence | 1. Patterns of ART use when participating in a specific activity 2. Adherence self-efficacy | Understanding patient behaviour  Identification of factors to intervene on |
| Ketchaji A, Assah F, Fokam J, Tanue EA, Monebenimp F, Ngowe MN. Predictors of non-adherence to antiretroviral therapy among adolescents living with HIV in the Centre Region of Cameroon. *Am J Public Heal Res*. 2019;7(4):126-136. http://pubs.sciepub.com/ajphr/7/4/1/index.html | 2019 | Cameroon | Cross sectional | Means to an end | Adherence | 1. Composite measure of adherence 1a. Self report 1b. Pill count | Understanding patient behaviour  Identification of factors to intervene on |
| Kim E, Ndege PK, Jackson E, Clauw DJ, Ellingrod VL. Patient perspectives on medication self-management in rural Kenya: a cross-sectional survey. Int J Qual Heal care J Int Soc Qual Heal Care. 2019;31(5):353-358. doi:10.1093/intqhc/mzy187 | 2019 | Kenya | Cross sectional | Not primary purpose but evaluates partially | Other | Measures of Drug Self-Management Scale (MeDS) | Understanding patient behaviour |
| Kim MH, Tembo TA, Mazenga A, et al. The Video intervention to Inspire Treatment Adherence for Life (VITAL Start): protocol for a multisite randomized controlled trial of a brief video-based intervention to improve antiretroviral adherence and retention among HIV-infected pregnant women in . Trials. 2020;21(1):207. doi:10.1186/s13063-020-4131-8 | 2020 | Malawi | RCT | Means to an end | Other | 1. Composite outcome of engagement 1a. Adherence measure by VL 1b. Retention measured by attrition/ in care  2. Behavioural adherence 2a. Self reported adherence 2b. Pharmacy refill adherence 2c. Tenofovir diphosphate at 12 months | Development and evaluation of interventions |
| Kimulwo MJ, Okendo J, Aman RA, et al. Plasma nevirapine concentrations predict virological and adherence failure in Kenyan HIV-1 infected patients with extensive antiretroviral treatment exposure. PLoS One. 2017;12(2):e0172960. doi:10.1371/journal.pone.0172960 | 2017 | Kenya | Mixed Methods | Main focus | Adherence | 1. Plasma ART concentration (nevirapine)  Comparisons 2. VL 3. Adherence  3a. Self report 3b. Pill count | Individual patient evaluation  Support ART management decisions (through initiating conversations with patients or providing healthcare workers with information) |
| Kioko MT, Pertet AM. Factors contributing to antiretroviral drug adherence among adults living with HIV or AIDS in a Kenyan rural community. African J Prim Heal care Fam Med. 2017;9(1):e1-e7. doi:10.4102/phcfm.v9i1.1343 | 2017 | Kenya | Cross sectional | Not primary purpose but evaluates partially | Adherence | 1. Patient Medicine Adherence Questionnaire 2. Self reported missed pills 3.Home-based pill count adherence | Understanding patient behaviour  Identification of factors to intervene on |
| Koss CA, Natureeba P, Mwesigwa J, et al. Hair concentrations of antiretrovirals predict viral suppression in HIV-infected pregnant and breastfeeding Ugandan women. AIDS. 2015;29(7):825-830. doi:10.1097/QAD.0000000000000619 | 2015 | Uganda | Secondary analysis | Main focus | Adherence | 1. Hair ARV concentrations  2. Self- report  Comparisons 3. VL | Understanding patient behaviour |
| Luma HN, Mbatchou Ngahane BH, Mapoure YN, et al. Cross-sectional assessment of three commonly used measures of adherence to combination antiviral therapy in a resource limited setting. Int J STD AIDS. 2017;28(1):69-76. doi:10.1177/0956462415627394 | 2017 | Cameroon | Cross sectional | Main focus | Adherence | 1. Self reported adherence by visual analog scale (VAS) 2. Self reported adherence by 4 day recall (FDR) 3. Clinic attendance (as a measure of adherence in this study)  Comparisons 4. CD4 | Support ART management decisions (through initiating conversations with patients or providing healthcare workers with information) |
| Maclachlan EW, Shepard-Perry MG, Ingo P, et al. Evaluating the effectiveness of patient education and empowerment to improve patient-provider interactions in antiretroviral therapy clinics in Namibia. AIDS Care. 2016;28(5):620-627. doi:10.1080/09540121.2015.1124975 | 2015 | Namibia | RCT | Means to an end | Other | Measure of patient-provider interaction quality 1. Doctor factors in interaction 1.1 Doctor verbal dominance 1.2 Facilitation and patient activation 1.3 Doctor positive affect 1.4 Patient centredness 1.5 Doctor information gathering  2. Patient factors in interaction 2.1 All patient question asking 2.2. Patient activation and engagement 2.3 Patient positive affect  3. Global affect 3.1 Doctor global affect (positive affect, dominance and assertiveness, interactivity) 3.2 Patient global affect (positive affect, interactivity)  4. Empowerment - did not measure directly, but measured indirectly though the differences between the intervention and control groups | Development and evaluation of interventions |
| Magidson JF, Saal W, Nel A, Remmert JE, Kagee A. Relationship between depressive symptoms, alcohol use, and antiretroviral therapy adherence among HIV-infected, clinic-attending patients in South Africa. J Health Psychol. 2017;22(11):1426-1433. doi:10.1177/1359105316628743 | 2017 | South Africa | Cross sectional | Means to an end | Adherence | Self-reported weekend non adherence | Understanding patient behaviour  Identification of factors to intervene on |
| Mbengue MAS, Chasela C, Onoya D, Mboup S, Fox MP, Evans D. Clinical predictor score to identify patients at risk of poor viral load suppression at six months on antiretroviral therapy: results from a prospective cohort study in Johannesburg, South Africa. Clin Epidemiol. 2019;11:359-373. doi:10.2147/CLEP.S197741 | 2019 | South Africa | Prospective cohort | Main focus | Other | 1. Clinical prediction score - Baseline characteristics 1a. Age 1b. Gender 1c. WHO stage - Variables at 6 months on ART 1d. Platelet count 1e. Mean cell volume (MCV) increase 1f. Retention: days late for ART visit 1g. Adherence: VAS  Comparisons Adherence:  1g. VAS 2. Simplified Medication Adherence Questionnaire (SMAQ) 3. Pill identification test 4. Composite measure of adherence  5. VL as the gold standard | Flag patients early for intervention |
| Mbengue MAS, Sarr SO, Diop A, Ndour CT, Ndiaye B, Mboup S. Prevalence and determinants of adherence to antiretroviral treatment among HIV patients on first-line regimen: A cross-sectional study in Dakar, Senegal. Pan Afr Med J. 2019;33:1-13. doi:10.11604/pamj.2019.33.95.17248 | 2019 | Senegal | Cross sectional | Means to an end | Adherence | 1. Composite adherence measure 1a. 4 day recall self report 1b. Visual analog scale self report 1c. Simplified Medication Questionnaire (SMAQ) | Routine monitoring and evaluation of programmes at facility and population levels for quality improvement and strategic decision-making  Understanding patient behaviour  Identification of factors to intervene on |
| McKinney O, Modeste NN, Lee JW, Gleason PC. Predicting Malawian Women’s Intention to Adhere to Antiretroviral Therapy. J Public health Res. 2015;4(2):533. doi:10.4081/jphr.2015.533 | 2015 | Malawi | Cross sectional | Not primary purpose but evaluates partially | Other | 1. Adherence intention 2. Perceived behavioural control   Comparisons 3. Adherence | Understanding patient behaviour  Identification of factors to intervene on |
| Mekuria LA, Prins JM, Yalew AW, Sprangers MAG, Nieuwkerk PT. Which adherence measure - self-report, clinician recorded or pharmacy refill - is best able to predict detectable viral load in a public ART programme without routine plasma viral load monitoring? Trop Med Int Health. 2016;21(7):856-869. doi:10.1111/tmi.12709 | 2016 | Ethiopia | Retrospective cohort | Main focus | Adherence | 1. Self reported adherence  2. Clinician Recorded Adherence  3. Pharmacy Refill | Rationalise resources (such as viral load testing) |
| Meresse M, March L, Kouanfack C, et al. Patterns of adherence to antiretroviral therapy and HIV drug resistance over time in the Stratall ANRS 12110/ESTHER trial in Cameroon. HIV Med. 2014;15(8):478-487. doi:10.1111/hiv.12140 | 2014 | Cameroon | Prospective cohort | Main focus | Adherence | 1. Pattern of adherence  Comparison 2. Development of resistance 2a. Early resistance 2b. Late resistance | Routine monitoring and evaluation of programmes at facility and population levels for quality improvement and strategic decision-making |
| Mody A, Eshun-Wilson I, Sikombe K, et al. Longitudinal engagement trajectories and risk of death among new ART starters in Zambia: A group-based multi-trajectory analysis. PLoS Med. 2019;16(10):1-25. doi:10.1371/journal.pmed.1002959 | 2019 | Zambia | Retrospective cohort | Not primary purpose but evaluates partially | Other | 1. Engagement trajectory 1a. Adherence  1b. Retention  Comparisons 2. Mortality | Routine monitoring and evaluation of programmes at facility and population levels for quality improvement and strategic decision-making  Flag patients early for intervention |
| Mongo-Delis A, Mombo LE, Mickala P, et al. Factors associated with adherence to ARV treatment in people living with HIV/AIDS in a rural area (Koula-Moutou) in East Gabon. Afr J AIDS Res. 2019;18(1):51-57. doi:10.2989/16085906.2018.1552878 | 2019 | Gabon | Descriptive | Main focus | Adherence | 1. DECLARATIVE ADHERENCE 1a. Quantitative declarative adherence 1b. Qualitative declarative adherence 2. PHARMACY VIST COMPLIANCE 2a. Duration of ART 2b. Last 9 months | Understanding patient behaviour  Identification of factors to intervene on |
| Mudhune V, Gvetadze R, Girde S, et al. Correlation of Adherence by Pill Count, Self-report, MEMS and Plasma Drug Levels to Treatment Response Among Women Receiving ARV Therapy for PMTCT in Kenya. AIDS Behav. 2018;22(3):918-928. doi:10.1007/s10461-017-1724-7 | 2017 | Kenya | Secondary analysis | Main focus | Adherence | 1. Pill count 2. Self-report 3. MEMS 4. Plasma drug level | Routine monitoring and evaluation of programmes at facility and population levels for quality improvement and strategic decision-making |
| Mûnene E, Ekman B. Association between patient engagement in HIV care and antiretroviral therapy medication adherence: cross-sectional evidence from a regional HIV care center in Kenya. AIDS Care. 2015;27(3):378-386. doi:10.1080/09540121.2014.963020 | 2015 | Kenya | Cross sectional | Main focus | Retention | 1. Engagement in care index  Comparisons 2. Mean medication adherence since enrolment | Understanding patient behaviour |
| Mungwira RG, Divala TH, Nyirenda OM, et al. A targeted approach for routine viral load monitoring in Malawian adults on antiretroviral therapy. Trop Med Int Health. 2018;23(5):526-532. doi:10.1111/tmi.13047 | 2018 | Malawi | Cross sectional | Main focus | Other | 1. Summary score to predict viral failure  Comparison 2. VL | Rationalise resources (such as viral load testing) |
| Murnane PM, Bacchetti P, Currier JS, et al. Tenofovir concentrations in hair strongly predict virologic suppression in breastfeeding women. AIDS. 2019;33(10):1657-1662. doi:10.1097/QAD.0000000000002237 | 2019 | South Africa, Malawi, Tanzania, Uganda, Zambia, Zimbabwe | Prospective cohort | Main focus | Adherence | 1. Hair ARV concentration (tenofovir)  Comparisons 2. VL | Understanding patient behaviour |
| Musumari PM, Wouters E, Kayembe PK, et al. Food insecurity is associated with increased risk of non-adherence to antiretroviral therapy among HIV-infected adults in the Democratic Republic of Congo: a cross-sectional study. PLoS One. 2014;9(1):e85327. doi:10.1371/journal.pone.0085327 | 2014 | Congo | Cross sectional | Means to an end | Adherence | 1. Composite measure of adherence 1a. 6 month Pharmacy refill 1b. Self report | Understanding patient behaviour  Identification of factors to intervene on |
| Myer L, Phillips TK, Zerbe A, et al. Integration of postpartum healthcare services for HIV-infected women and their infants in South Africa: A randomised controlled trial. PLoS Med. 2018;15(3):e1002547. doi:10.1371/journal.pmed.1002547 | 2018 | South Africa | RCT | Means to an end | Combination | 1. Composite outcome 1a. Retention in care 1b. Viral suppression | Development and evaluation of interventions |
| Nduaguba SO, Soremekun RO, Olugbake OA, Barner JC. The relationship between patient-related factors and medication adherence among Nigerian patients taking highly active anti-retroviral therapy. Afr Health Sci. 2017;17(3):738-745. doi:10.4314/ahs.v17i3.16 | 2017 | Nigeria | Cross sectional | Means to an end | Adherence | 1. Two-week self-recall 2. Barriers to adherence 3. Treatment satisfaction | Understanding patient behaviour  Identification of factors to intervene on |
| Ngara B, Zvada S, Chawana TD, Stray-Pedersen B, Nhachi CFB, Rusakaniko S. A population pharmacokinetic model is beneficial in quantifying hair concentrations of ritonavir-boosted atazanavir: a study of HIV-infected Zimbabwean adolescents. BMC Pharmacol Toxicol. 2020;21(58). doi:http://dx.doi.org/10.1186/s40360-020-00437-y | 2020 | Zimbabwe | Secondary analysis | Main focus | Adherence | 1. Hair ARV concentration  Comparison 2. Self reported adherence | Individual patient evaluation |
| Nnambalirwa M, Govathson C, Evans D, McNamara L, Maskew M, Nyasulu P. Markers of poor adherence among adults with HIV attending Themba Lethu HIV Clinic, Helen Joseph Hospital, Johannesburg, South Africa. Trans R Soc Trop Med Hyg. 2016;110(12):696-704. doi:10.1093/trstmh/trx003 | 2016 | South Africa | Retrospective cohort | Main focus | Adherence | 1. Last self-reported adherence 2. Missed clinic visits 2a. Total missed visits 2b. Missed refill visits 2c. Missed clinical visits 3. Mean corpuscular volume (MCV) 3a. MCV at visit 3b. MCV difference 4. CD4 4a. CD4 at visit 4b. CD4 difference | Rationalise resources (such as viral load testing)  Support ART management decisions (through initiating conversations with patients or providing healthcare workers with information) |
| Nutor JJ, Slaughter-Acey JC, Marquez SP, DiMaria-Ghalili RA, Momplaisir F, Jemmott LS. Influence of toilet access on antiretroviral adherence intention among pregnant and breastfeeding women who are HIV-positive and enrolled in Option B. Health Care Women Int. Published online April 2020:1-15. doi:10.1080/07399332.2020.1746791 | 2020 | Zambia | Cross sectional | Means to an end | Other | Adherence intention | Understanding patient behaviour  Identification of factors to intervene on |
| Nutor JJ, Slaughter-Acey JC, Marquez SP, et al. Impact of attitudes and beliefs on antiretroviral treatment adherence intention among HIV-positive pregnant and breastfeeding women in Zambia. BMC Public Health. 2020;20(1). doi:10.1186/s12889-020-09505-8 | 2020 | Zambia | Cross sectional | Means to an end | Other | Adherence intention | Understanding patient behaviour  Identification of factors to intervene on |
| Okatch H, Beiter K, Eby J, et al. Brief Report: Apparent Antiretroviral Overadherence by Pill Count is Associated With HIV Treatment Failure in Adolescents. J Acquir Immune Defic Syndr. 2016;72(5):542-545. doi:10.1097/QAI.0000000000000994 | 2016 | Botswana | Prospective cohort | Main focus | Adherence | 1. Overadherence by pill count  Comparisons 2. Adherence  3. VL | Flag patients early for intervention |
| Okoronkwo I, Ishaku S, Chinweuba A, Akpan-Idiok P, Ihudiebube C, Odira C. Assessing self care practices of people living with AIDS attending antiretroviral clinic Kafanchan, Kaduna State, Nigeria. J AIDS Clin Res. 2015;6(12):528. http://www.omicsonline.org/open-access/assessing-self-care-practices-of-people-living-with-aids-attendingantiretroviral-clinic-kafanchan-kaduna-state-nigeria-2155-6113-1000528.php?aid=66393 | 2015 | Nigeria | Cross sectional | Means to an end | Other | Self care | Understanding patient behaviour |
| Ongubo DM, Lim R, Tweya H, et al. A cross-sectional study to evaluate second line virological failure and elevated bilirubin as a surrogate for adherence to atazanavir/ritonavir in two urban HIV clinics in Lilongwe, Malawi. BMC Infect Dis. 2017;17(1):461. doi:10.1186/s12879-017-2528-0 | 2017 | Malawi | Cross sectional | Main focus | Adherence | 1. Bilirubin as a measure of adherence to atazanavir/ritonavir 2. Pill count adherence  Comparison  3. VLpl | Rationalise resources (such as expensive genotypes) |
| Orne-Gliemann J, Font H, Maphosa T, et al. Patterns of Attendance at Mother Support Groups in Zimbabwe. The EPAZ Trial (2014-2016). J Acquir Immune Defic Syndr. 2017;75 Suppl 2:S216-S223. doi:10.1097/QAI.0000000000001348 | 2017 | Zimbabwe | Secondary analysis | Means to an end | Retention | Attendance at club visits 1. Frequency of visits 2. Pattern of attendance  Comparison 3. Retention in care at 12 months | Understanding patient behaviour |
| Orrell C, Cohen K, Leisegang R, Bangsberg DR, Wood R, Maartens G. Comparison of six methods to estimate adherence in an ART-naïve cohort in a resource-poor setting: which best predicts virological and resistance outcomes? AIDS Res Ther. 2017;14(1):20. doi:10.1186/s12981-017-0138-y | 2017 | South Africa | Prospective cohort | Main focus | Adherence | 1. 3 day self-report (SR) 2. clinic-based pill count (CPC) 3. Pharmacy refill: average adherence (Prave) 4. Pharmacy refill medication--free days (Prgap) 5. Efavirenz drug monitoring (EDM) 6. Electronic adherence monitoring device (EAMD) | Routine monitoring and evaluation of programmes at facility and population levels for quality improvement and strategic decision-making  Individual patient evaluation |
| Peterson K, Menten J, Peterson I, et al. Use of Self-Reported Adherence and Keeping Clinic Appointments as Predictors of Viremia in Routine HIV Care in the Gambia. J Int Assoc Provid AIDS Care. 2015;14(4):343-347. doi:10.1177/2325957413500344 | 2015 | The Gambia | Prospective cohort | Main focus | Combination | 1. Self reported adherence 1a. Previous 1 week 1b. Previous 4 weeks 2. Appointment adherence | Flag patients early for intervention |
| Phillips T, Brittain K, Mellins CA, et al. A Self-Reported Adherence Measure to Screen for Elevated HIV Viral Load in Pregnant and Postpartum Women on Antiretroviral Therapy. AIDS Behav. 2017;21(2):450-461. doi:10.1007/s10461-016-1448-0 | 2017 | South Africa | Cross sectional | Main focus | Adherence | Self report | Flag patients early for intervention |
| Phillips TK, Orrell C, Brittain K, Zerbe A, Abrams EJ, Myer L. Measuring retention in HIV care: the impact of data sources and definitions using routine data. AIDS. 2020;34(5):749-759. doi:10.1097/QAD.0000000000002478 | 2020 | South Africa | Secondary analysis | Main focus | Retention | 1. Retention 1a. 180 day visit gap 1b. 6 month visit constancy 1c. 12 month visit constancy 1d. HRSA-HRB: ≥90 days between visits  Comparison 2. VL        . | Routine monitoring and evaluation of programmes at facility and population levels for quality improvement and strategic decision-making |
| Phillips TK, Sinxadi P, Abrams E, et al. A comparison of plasma efavirenz and tenofovir, dried blood spot tenofovir-diphosphate, and self-reported adherence to predict virologic suppression among South African women. J Acquir Immune Defic Syndr. 2019;81(3):311-318. | 2019 | South Africa (Gugulethu) | Cross sectional | Main focus | Adherence | 1. Plasma ART concentration 1a. Tenofovir 1b. Efavirenz 2. Dried Blood spot ART concentration (Tenofovir diphosphate) 3. Self report 4. White coat adherence | Support ART management decisions (through initiating conversations with patients or providing healthcare workers with information) |
| Phillips TK, Wilson IB, Brittain K, et al. Decreases in Self-Reported ART Adherence Predict HIV Viremia Among Pregnant and Postpartum South African Women. J Acquir Immune Defic Syndr. 2019;80(3):247-254. doi:10.1097/QAI.0000000000001909 | 2019 | South Africa (Gugulethu) | Prospective cohort | Main focus | Adherence | Change in self-reported adherence | Rationalise resources (such as viral load testing)  Support ART management decisions (through initiating conversations with patients or providing healthcare workers with information) |
| Platt L, Xu A, Giddy J, et al. Identifying and predicting longitudinal trajectories of care for people newly diagnosed with HIV in South Africa. PLoS One. 2020;15(9):e0238975. doi:10.1371/journal.pone.0238975 | 2020 | South Africa | Secondary analysis | Means to an end | Other | CD4 trajectory | Flag patients early for intervention |
| Platt MO, Evans D, Keegan PM, et al. Low-Cost Method to Monitor Patient Adherence to HIV Antiretroviral Therapy Using Multiplex Cathepsin Zymography. Mol Biotechnol. 2016;58(1):56-64. doi:10.1007/s12033-015-9903-0 | 2016 | South Africa | Prospective cohort | Main focus | Adherence | 1. Multiplex Cathepsin Zymography  Comparisons 2. Self report using SMAQ 3. VL | Routine monitoring and evaluation of programmes at facility and population levels for quality improvement and strategic decision-making |
| Poles G, Li M, Siril H, et al. Factors associated with different patterns of nonadherence to HIV care in Dar es Salaam, Tanzania. J Int Assoc Provid AIDS Care. 2014;13(1):78-84. doi:10.1177/1545109712467068 | 2014 | Tanzania | Prospective cohort | Not primary purpose but evaluates partially | Retention | Retention (visit adherence) 1. Visit constancy 2. Gaps in care 3. Visit in last quarter | Understanding patient behaviour |
| Rachlis B, Cole DC, van Lettow M, Escobar M. Survival functions for defining a clinical management Lost To Follow-Up (LTFU) cut-off in Antiretroviral Therapy (ART) program in Zomba, Malawi. BMC Med Inform Decis Mak. 2016;16:52. doi:10.1186/s12911-016-0290-7 | 2016 | Malawi | Retrospective cohort | Main focus | Retention | Loss to follow up | Rationalise resources (such as resources to trace those lost to follow up) |
| Rhead R, Masimirembwa C, Cooke G, et al. Might ART Adherence Estimates Be Improved by Combining Biomarker and Self-Report Data? PLoS One. 2016;11(12):e0167852. doi:10.1371/journal.pone.0167852 | 2016 | Zimbabwe | Cross sectional | Main focus | Adherence | 1. Combination measure of adherence 1a. ARV drug concentration 1b. Self-reported adherence | Routine monitoring and evaluation of programmes at facility and population levels for quality improvement and strategic decision-making |
| Roy M, Holmes C, Sikazwe I, et al. Application of a Multistate Model to Evaluate Visit Burden and Patient Stability to Improve Sustainability of Human Immunodeficiency Virus Treatment in Zambia. Clin Infect Dis an Off Publ Infect Dis Soc Am. 2018;67(8):1269-1277. doi:10.1093/cid/ciy285 | 2018 | Zambia | Retrospective cohort | Means to an end | Other | Visit attendance 1. Visit volume 2. Appointment intervals 3. Characterisation of patient state | Routine evaluation of engagement in patients in differentiated service delivery models |
| Sangeda RZ, Mosha F, Prosperi M, et al. Pharmacy refill adherence outperforms self-reported methods in predicting HIV therapy outcome in resource-limited settings. BMC Public Health. 2014;14:1035. doi:10.1186/1471-2458-14-1035 | 2014 | Tanzania | Prospective cohort | Main focus | Adherence | 1. Pharmacy refill 2. Self reported adherence 2a. Visual analogue scale (VAS) 2b. Swiss HIV Cohort Study Adherence Questionnaire (SHCS-AQ) 3. Pill count 4. Appointment keeping adherence  Comparison 5. VL at 1 year = gold standard 6. CD4 | Rationalise resources (such as expensive genotypes or viral load testing) |
| Sikkema KJ, Mulawa MI, Robertson C, et al. Improving AIDS Care After Trauma (ImpACT): Pilot Outcomes of a Coping intervention Among HIV-Infected Women with Sexual Trauma in South Africa. AIDS Behav. 2018;22(3):1039-1052. doi:10.1007/s10461-017-2013-1 | 2018 | South Africa | RCT | Means to an end | Other | 1. Engagement 2. Adherence motivation 3. Coping  4. VL | Development and evaluation of interventions |
| Ssewamala FM, Byansi W, Bahar OS, et al. Suubi+Adherence study protocol: A family economic empowerment intervention addressing HIV treatment adherence for perinatally infected adolescents. Contemp Clin trials Commun. 2019;16:100463. doi:10.1016/j.conctc.2019.100463 | 2021 | Uganda | Prospective cohort | Means to an end | Adherence | 1. Primary outcome of engagement: Viral suppression  2. Adherence 2a. Self-reported adherence 2b. Home based, unannounced pill counts 2c. Adherene self-efficacy  Other 3. Self efficacy 4. Motivation to participate | Understanding patient behaviour |
| Tabb ZJ, Mmbaga BT, Gandhi M, et al. Antiretroviral drug concentrations in hair are associated with virologic outcomes among young people living with HIV in Tanzania. AIDS. 2018;32(9):1115-1123. doi:10.1097/QAD.0000000000001788 | 2018 | Tanzania | Cross sectional | Main focus | Adherence | 1. Self report 2. Hair drug concentration | Support ART management decisions (through initiating conversations with patients or providing healthcare workers with information) |
| Teklu AM, Yirdaw KD. Patients who restart antiretroviral medication after interruption remain at high risk of unfavorable outcomes in Ethiopia. BMC Health Serv Res. 2017;17(1):247. doi:10.1186/s12913-017-2172-9 | 2017 | Ethiopia | Retrospective cohort | Means to an end | Retention | 1. Treatment interruption  Comparison 2. Unfavourable treatment outcomes | Routine evaluation of engagement in patients who struggle with care |
| Thorman J, Björkman P, Tesfaye F, Jeylan A, Balcha TT, Reepalu A. Validation of the Viral Load Testing Criteria - an algorithm for targeted viral load testing in HIV-positive adults receiving antiretroviral therapy. Trop Med Int Health. 2019;24(3):356-362. doi:10.1111/tmi.13201 | 2019 | Ethiopia | Prospective cohort | Main focus | Other | 1. Viral Load Testing Criteria (VLTC)  Comparison 2. VL | Rationalise resources (such as viral load testing) |
| Tique JA, Howard LM, Gaveta S, et al. Measuring Health Literacy Among Adults with HIV Infection in Mozambique: Development and Validation of the HIV Literacy Test. AIDS Behav. 2017;21(3):822-832. doi:10.1007/s10461-016-1348-3 | 2016 | Mozambique | Cross sectional | Main focus | Other | HIV Health Literacy Test (HIV-LT) | Development and evaluation of interventions |
| Umar E, Levy JA, Bailey RC, Donenberg G, Hershow RC, Mackesy-Amiti ME. Virological Non-suppression and Its Correlates Among Adolescents and Young People Living with HIV in Southern Malawi. AIDS Behav. 2019;23(2):513-522. doi:10.1007/s10461-018-2255-6 | 2019 | Malawi | Cross sectional | Dual focus(main and applied to a question) | Adherence | 1. Pill count 2. Self report 2a. Likert scale 2b. Visual analogue scale (VAS) 3. ART Medication self-efficacy (HIV-ASES)  Comparison 4. VL | Understanding patient behaviour |
| Wachira J, Middlestadt S, Reece M, Peng C-YJ, Braitstein P. Physician communication behaviors from the perspective of adult HIV patients in Kenya. Int J Qual Heal care. 2014;26(2):190-197. doi:10.1093/intqhc/mzu004 | 2014 | Kenya | Cross sectional | Not primary purpose but evaluates partially | Other | 1. Perceived physician communication behaviours  Comparison 2. Physician-patient relationship characteristics 3. Predisposition to attend clinic 4. Missed appointments 5. Missed ARV | Understanding patient behaviour |
| Wesevich A, Hosseinipour MC, Golin CE, et al. Female adherence self-efficacy before and after couple HIV testing and counseling within Malawi’s Option B+ program. AIDS Care. 2020;32(2):170-174. doi:10.1080/09540121.2019.1634789 | 2020 | Malawi | Prospective cohort | Means to an end | Other | 1. Adherence self-efficacy  Comparison 2. Self reported adherence | Development and evaluation of interventions |
| Zerbe A, Brittain K, Phillips TK, et al. Community-based adherence clubs for postpartum women on antiretroviral therapy (ART) in Cape Town, South Africa: a pilot study. BMC Health Serv Res. 2020;20(1):621. doi:10.1186/s12913-020-05470-5 | 2020 | South Africa | Non randomised interventional | Means to an end | Other | 1. Composite outcome 1a. Retention in care 1b. Viral suppression | Development and evaluation of interventions |
| Grey literature | | | | | | |  |
| Nawar E. Longitudinal analysis of interruptions in HIV care and treatment among HIV-positive pregnant women engaged in clinical care in Kigali, Rwanda. Diss Abstr Int Sect A Humanit Soc Sci. 2020;81(7-A):No-Specified. http://ovidsp.ovid.com/ovidweb.cgi?T=JS&PAGE=reference&D=psyc17&NEWS=N&AN=2020-10497-132 | 2019 | Rwanda | Prospective cohort | Dual focus(main and applied to a question) | Retention | Patterns of interruption 1. Number of interruptions 2. Length of longest interruption (duration) 3. Total number missed visits 4. Timing of interruption stratefied by length of interruption, relative to a fixed point (e.g. enrolment or initiation) 5. Cumulative duration of all interruptions in a period | Development and evaluation of interventions |
| Davis A, Norcini-Pala A, Nguyen N, et al. Longitudinal ART Adherence Trajectories and Sociodemographic and Psychosocial Predictors among ART Initiators in Cape Town, South Africa. In: Adherence 2019 Conference. International Association of Providers of AIDS Care; 2019. | 2020 | South Africa | Secondary analysis | Means to an end | Adherence | Adherence trajectory | Understanding patient behaviour |
| Nguyen N, Robbins R, Courtney I, et al. Can Self-Reported Adherence Predict ART Adherence Assessed by an Electronic Monitoring Device (Wisepill) in Resource-Constrained Settings in Cape Town, South Africa? In: Adherence 2019 Conference. ; 2019. | 2019 | South Africa | Secondary analysis | Dual focus(main and applied to a question) | Adherence | 1. Self-reported adherence  2. MEMS | Flag patients early for intervention |
| Google search | | | | | | |  |
| Published | | | | | | |  |
| Gosset A, Protopopescu C, Larmarange J. Retention in Care Trajectories of HIV-Positive Individuals Participating in a Universal Test-and-Treat Program in Rural South Africa (ANRS 12249 TasP Trial ). J Acquir Immune Defic Syndr. 2019;80(4):375-385. | 2018 | South Africa | RCT | Means to an end | Retention | 1. Retention in care 2. Retention trajectory | Routine monitoring and evaluation of programmes at facility and population levels for quality improvement and strategic decision-making |
| Jackson IL, Okonta JM, Ukwe C V. Development and psychometric evaluation of the patient’s HIV knowledge questionnaire (PHKQ). Int J Clin Pharm. 2020;42(2):695-702. doi:10.1007/s11096-020-00963-z | 2020 | Nigeria | Mixed Methods | Main focus | Other | The Patient’s HIV Knowledge Questionnaire | Development and evaluation of interventions |
| Barchi F, Winter SC, Ramaphane P, Dougherty D. The role of self-efficacy in women’s health-seeking behaviors in northwestern Botswana. J Health Care Poor Underserved. 2019;30(2):653-667. doi:10.1353/hpu.2019.0048 | 2019 | Botswana | Cross sectional | Dual focus(main and applied to a question) | Other | 1. Health-seeking behaviour 2. Generalised Self-efficacy Scale (GSE) 3. Health status | Understanding patient behaviour |
| Grey literature | | | | | | |  |
| Joshi MP, Clark A, Ludman M. Systems-Based Approaches to Improving Medication Adherence.; 2016. http://www.ijcm.org.in/text.asp?2016/41/1/55/170970 | 2016 | Global | Report | Not primary purpose but evaluates partially | Adherence | 1. Directly observed therapy 2. Biochemical measurement 3. Pharmacy records 4. Pill count 5. Medication events monitoring system 6. Tracking appointment attendence 7. Self report | Routine monitoring and evaluation of programmes at facility and population levels for quality improvement and strategic decision-making |
| World Health Organization. HIV Test–Treat–Retain Cascade Analysis: Guide and Tools.; 2017. | 2017 | Global | Report | Main focus | Combination | 1. HIV Care Coverage 2. ART coverage 3. ART retention 4. VL suppression | Routine monitoring and evaluation of programmes at facility and population levels for quality improvement and strategic decision-making |
| Stakeholder suggested | | | | | | |  |
| Grey literature | | | | | | |  |
| Jennings L, Kellerman T, Spinelli M, et al. Drug resistance, rather than low tenofovir levels in blood or urine, is associated with tenofovir, emtricitabine and efavirenz (TEE) failure in resource-limited settings. PREPRINT. Published online 2021. | 2021 | South Africa | Cross sectional | Dual focus(main and applied to a question) | Adherence | 1. Urine tenofovir concentration 2. Self reported adherence 3. Pharmacy refill 4. Viral load 5. HIV resistance  6. Intracellular tenofovir diphosphate  Comparison  7. Viral load | Understanding patient behaviour |

## Table 5: List of measures removed during analysis

*This list describes measures that were identified in the data extraction as possibly reflecting the measurement of engagement, but were later removed as the list was refined. They were excluded as, during discussions with stakeholders, they were felt to reflect factors that affect engagement rather than engagement behaviour itself.*

| HEALTH SYSTEM FACTORS | INDIVIDUAL FACTORS | |
| --- | --- | --- |
| **Provider-patient relationship characteristics and communication**  Time spent in consultation  Trust in provider  Decision-making role preference  Physician communication behaviours  Patient perception of engagement with healthcare provider  **Coverage**  HIV Care coverage  ART coverage  Self-reported ART coverage | **ENGAGEMENT ABILITY (categorised according to the Situated Information Motivation Behavioral Skills Model of Health Care Initiation and Maintenance model**5**)** | |
|  | **Information and beliefs**  HIV Health Literacy Test (HIV-LT)  ART knowledge  The Patient’s HIV Knowledge Questionnaire  Pill identification  Beliefs about medication  **Motivation**  Motivation to adhere to ART  Personal adherence motivation  Intrinsic motivation  ART satisfaction  Perceived behavioural control  Intention to adhere to ART  Internalised AIDS-related stigma scale (IA-RSS) | **Skills and self-efficacy**  HIV self-efficacy  HIV adherence self-efficacy scale (HIV-ASES)  Culturally-adapted 10-item Adherence self-efficacy  General health self-efficacy  Generalised self-efficacy scale (GSE)  Chronic disease self-efficacy  Coping self-efficacy  Pictogram ability self-report  Morisky Scale  Medication autonomy  Resilience  **Composite of ability**  Life Windows Information Motivation Behavioral Skills ART Adherence Questionnaire (LW-IMB-AAQ).  Adapted from the Patient Medicine Adherence Questionnaire (PMAQ) |
| CONTEXTUAL FACTORS |  |  |
| Quantification of barriers to adherence  Revised AACTG reasons for non-adherence |  |  |

##

## Table 6: Measures of retention with detailed information on their use

| Measure | Description | | Data required | | Evaluation | | | Ref |
| --- | --- | --- | --- | --- | --- | --- | --- | --- |
|  | Definition | Pattern and duration of measurement | Data collected and source | Processing: presentation, scoring, calculation, categorisation and interpretation | Strengths | Limitations | Evidence (for association with clinical outcomes/ other elements of engagement/ validity) |  |
| In care | | | | | | | | |
| Visit attendance | Number of visits in a time period | Reflects the number of visits in a defined interval  Patient perspective: by time since ART initiation  Programme perspective: by calendar date ^1^ | From routine patient record  Visit dates^2^  Missed visits: scheduled appointments | **Presentation:**   - The median number of visits^3^ - Binary outcome of having attended a specific number of visits above a threshold within a specific time period^2,3^ - Proportion of visits by type of encounter^1^   **Categorisation:**  Retained:  Attending ≥1 visit in the time period^3^  OR  ≥4 times in 12 months^2^ | Directly measures retention | Attendance measures assume that medication is taken and do not automatically imply adherence^3^ | A binary visit frequency outcome produced higher estimates of retention (55% for ≥4visits in a year) than missed visits, visit constancy, single time point retention and gaps in care, but had a lower estimate of retention compared to the HRSA definition (76%) ^2^  A binary visit frequency (threshold ≥4visits in a year) had higher NPVs for detecting retention compared to missed visits, visit constancy, single time point retention and gaps in care^2^ | ^3^  ^2^  ^1^ |
| Average retention | Proportion of kept visits^4^ or expected visits attended in a time period^3^  Also used as a measure of adherence^5^  Considered kept/ attended if within a specified window around the expected or scheduled date:   - Window e.g. ±3^6^ - Within 7, 14 days, 30 days or 60 days after the scheduled/ expected appointment^2,7^ - Define retention cut-off after an expected or scheduled visit e.g. ≥9 weeks^8^, >90 days (WHO definition^9,10^) or >180 days without a visit^11^ (early visits considered on-time)^10^ | Reflects the number of visits in a defined interval ^5^ | From routine patient records  Visit dates  Expected visits: refill duration  Missed visits: appointment date^4,5^ | **Calculation:**  Visit adherence = number appointments attended/ number appointments schedule or expected*100^5^  Proportion of possible visits attended: calculate the number of visits expected in the time period based on the usual refill duration  **Categorisation:**  Optimal retention: 100%, visits ≥95% or ≥80% attendance  Not retained or suboptimal retention: <100%, <95% or <80% attendance^4,12^ |  |  | ≥80% retention was associated with viraemia (VL>400 copies/mL)  ROC AUC: 0.53, 0.59 adjusted for CD4. Sensitivity 42%, specificity 76% ^4^  ≥95% clinic attendance associated with CD4 ≥200 cells/mm3:  adjOR 2.6 [1.3-5.9]^5^  Not correlated with self-reported measures of adherence, producing lower estimates of engagement that four-day recall but higher than the visual analogue scale (73% vs 83% and 68% respectively)^5^ | ^4^  ^2^  ^3^  ^5^ |
| Engagement in care index | Measure that weights the average retention using an engagement index to adjust for re-engagements (clinic visit >90 days from scheduled visit)^6^ | Reflects the number of visits in a defined interval | From routine patient records  HIV visits^6^ | **Calculation:**  Average retention = expected or scheduled visits attended on time/ total number scheduled visits ^6^  Engagement index = visit constancy/log(number of reengagements)x2 ^6^  - Multiplying the denominator by a constant eliminated log 1 --> means a denominator of 1 indicated individuals who had never re-engaged^6^ | The weighting transformed average retention into a more robust definition of engagement in HIV care^6^  Incorporating reengagement as part of the variable defining engagement, captures the theoretical progression from poor attendance to eventual attrition in a single measure^6^.  Easy to interpret^6^ | Doesn’t consider dynamic nature of engagement and the possible time-dependent characteristic of serial adherence measures^6^ | A unit increase in the HIV care engagement index was associated with a 9.2% [3.2-15.1] increase in medication adherence (adjusted for sociodemographics, health status and treatment factors)^6^ | ^6^ |
| Fixed point retention | Evidence of interaction with the health system at a specific time point ^13^ regardless of the continuity of care before^14^.  Non-retention is synonymous with attrition, and if the patient is not retained they are considered ‘lost to follow up’^8^. This is a retrospective definition of loss to follow up: defined based on whether the individual was considered to have been in care in a window before the analysis date^15^  Use a window or period after an expected visit to define the time period for a visit to occur   - Window around the time point, e.g. window of 9-18 months^13^ or 331-390 days for one year retention^3^, 180 days around the fixed time point^11^ or 2 years without a laboratory result ^14^ - Window beyond the time point e.g. presence in care ≥12 months post initiation (12 months or any time thereafter) ^10^ - Define retention cut-off after an expected or scheduled visit e.g. ≥9 weeks^8^, >90 days (WHO definition^9,10,16^) or >180 days without a visit^11^ (early visits considered on-time)^10^ | Summary patient-level measure ^11^  Visit level data  Reflects retention at a specific point, generally six, 12, 18, 24 or 36 months from a fixed time point^11^ (e.g. after initiation of treatment or an intervention^11,13^) or in a specific interval (e.g. in a specific calendar quarter^17^) | From patient routine records  Date of evidence of a visit/ interaction with the health system ^8,10,11,13,17^  Status (with or without weighting or adjustment for tracing): transferred out, died or stopped taking ART^18^  ART refill (# dispensed pills at each visit) to calculate expected visit date^8^  For denominator need all those who initiated care in the preceding interval if calculating retention according to time since initiation (those who  start ART between 27 and 15 months  before the survey start date for 12-month retention)^16^ or all those who should be in care (ever initiated) | **Presentation:**   - Retention - Attrition (1 - retention^14^)   **Calculation:**  Those transferred out or who died are excluded from analysis^10,18^  **Categorisation:**  Retained if have evidence of interaction^13^ in a particular time period (window or period after an expected visit) ^17^  Non-retention/ attrition/ loss to follow up: not retained at a particular time point | Simple and easy to collect data and calculate result^2,10^  Clearly defines “out-of-care patients ^2^  NPV and PPV for viral suppression are similar to other definitions. Capture a large part of the information conveyed by the more complicated measures ^2^  Has high specificity across definitions and various windows for non-retention^10^  Evidence-based definitions of loss to follow up available to reduce the risk of misclassification^8^  Does not consider transient interruptions as complete non-retention, except for at the time of evaluation^15^  This retrospective definition is appropriate when non-retention is not the primary outcome e.g. a primary outcome of mortality, as it allows for more follow-up time than censoring patients at the first instance of non-retention^15^ | Point estimate of retention in care does not reflect continuity or regularity of care before this point^3^ and does not capture milestones before the point of analysis^10^  Considers transient interruptions at the time of evaluation to be non-retention^15^  Attendance measures assume that medication is taken and do not automatically imply medication adherence^3^  Missing data is a limitation to the accuracy of this metric ^8^  Summary measures are vulnerable to misclassification of competing outcomes (such as transfer or death) as non-retention, leading to overestimation of attrition (loss to follow up) and underestimation of retention^11^. Has higher misclassification than measures that evaluate patterns and the continuum of care ^10^  A universal definition of loss to follow up may not be appropriate . The ideal thresholds for defining a window or loss to follow up may be context specific (e.g. dependent on visit frequency guidelines), requiring calculation for each setting^8^  A retrospective definition limits standard survival analysis to model non-retention as an outcome, if loss to follow up is the primary outcome of interest^15^  Does not distinguish between those with good and those with poor measures of regimen adherence (timeliness, completeness, etc.)^16^ | Point retention produce both lower estimates of retention (55%) than number of isolated clinic visits (76%) or missed visits with a 60 day definition (63%) but higher than missed visits with a 14 day (30%) or 30 day (38%) definition, visit constancy (53%) or gaps in care (43%)^2^, and higher estimates of retention (86% for visit in last quarter) than visit constancy and gaps in care^17^  Point retention misclassified patients’ retention compared to a composite measure of point retention and ≥75% appointments met on time (with a 15 day window):   - Presence in care ≥12 months post initiation: 97.6% misclassified - WHO 3 months definition: 78.1% misclassified - Alive and in care at 12 months (Mozambique MoH definition): 54% misclassified^10^   Loss to follow up should be defined as ≥9 weeks late for an appointment, as this is the time frame which minimises misclassification of patients as lost to follow up – most (±70%) patients returned of their own accord within 9 weeks of missing an appointment ^8^ | ^13^  ^17^  ^3^  ^11^  ^10^  ^2^  ^14^  ^8^  ^9^  ^18^  ^15^  ^16^ |
| Re-engagement retention | Measures re-entry into the system (evidence of interaction with the system) after a gap in care (e.g. 180 days from the last appointment or 90 days from a missed appointment^27^) after the date of in-person tracing contact^27^)^18^. |  | From routine patient records  Evidence of interaction with the system after a gap.  Expected or scheduled appointment, visit dates ^27^ | **Presentation:**   - Status of re-engagement - Time to re-engagement   Proportion re-engaged for a population ^27^ |  | If patients receive a new patient number, or return to a new facility, when they return it is difficult to link them to those who have not been in care: may underestimate return^27^  Death is a competing risk but mortality data is often poor quality, underestimating the rate of return (misclassification)^27^ |  | ^27^  ^18^ |
| Continuity | | | | | | | | |
| Visit gap/ treatment interruption | Gap in care: duration of time without a visit ^22^ where the patient returns to care after the gap^23^  Gap can be defined as:   - One month without treatment (i.e. late for a visit by a month)^18^   or   - 60 days^17,22,24^ - 90 days^2,22^ - 120 days^17^ - 180 days^11,22^   without a visit | Cross sectional: presence of a gap in care in an interval or not ^22^ | From routine patient records  Visit dates ^11,17,22–24^  Scheduled or expected dates^23^  Treatment status - dead, lost to follow up, stopped treatment, transferred out | **Presentation:**   - Number of gaps in an interval^22,23^ - Number of missed visits in an interval (vs a gap which can cover multiple missed visits) ^23^ - Cumulative duration of all interruptions in a period^23^ - Duration of longest interruption^23^ - Binary outcome of the presence or absence of a treatment gap^18^ - Time to a gap from a fixed point^18^, can stratify by duration of interruption^23^   **Calculation:**  Need at least 1 visit in the interval to qualify for the analysis^11^  Either calculate time between visits or count missed scheduled/expected visits to calculate a gap  **Categorisation:**  Retained: interval between two visits is shorter than or equal to the defined gap  Late or not retained: interval between two visits is longer than the defined gap^17,24^ | Adds to the nuance and understanding of retention when combined with summary measures like point retention^11^ | Vulnerable to misclassification of competing outcomes (such as transfer or death) as non-retention, leading to overestimation of attrition (loss to follow up) and underestimation of retention^11,18^.  VL rebounds rapidly so vulnerable to misclassification if using later VL as gold standard^23^  Not suitable to indicate non-retention in differentiated models with longer monthly refills - needs to be adapted^22^ | A 180 day visit gap was associated with viral failure (VL >1000copies/mL) more strongly (and with higher sensitivity) compared to visit constancy and HRSA-HRB: OR 24.3 (12.0-48.9) with high sensitivity (93% [86-97])^22^.  A one month treatment gap was associated with unfavourable outcomes (including attrition, death or stopping treatment) with a hazard ration of 1.9 [1.5-2.4] if the gap was in the first 6 months, 2.4 [2.0-2.8] and a hazard ratio of 2.4-2.6 if the gap was in the first 12, 18 or 24 months^18^  Earlier gaps in care produced higher estimates of non-adherence than later gaps:  Gap 60-90 days: 22%, 90-120 days: 6%, ≥120 days: 22% ^17^  90 day gap produced a lower estimate of retention (43%) compared to number of isolated clinic visits, visit constancy, single time point retention, <25% missed visits and missed visits with a definition of 60 days, but a higher estimate than missed visits with a definition of 14 (30%) or 30 (38%) days ^2^. A 60 day gap had a higher estimation of non-adherence (49.6%) compared to visit constancy and a binary measure of retention ^17^  Number of interruptions associated with viral failure (VL>1000copies/mL) , with the adjOR for ≥4 interruptions: 4.95 [1.40-17.49], and viraemia with adjOR 2.52 [1.06-6.02] ^23^  Cumulative duration of all interruptions in a period associated with viral failure (VL>1000 copies/mL) with adjOR for ≥4 months missed 5.14 [1,64-16.12], and viraemia with the adjOR 2.77 [1.32-5.84] ^23^  Duration of longest interruption associated with viral failure (VL>1000 copies/mL) with the adjOR for ≥3 months interruption: 7.05 [2.13-23.29], and viraemia with adjOR 3.73 [1.64-8.50] ^23^ | ^18^  ^24^  ^17^  ^2^  ^22^  ^11^  ^23^ |
| Visit constancy | A measure reflecting the presence of ≥1 HIV visit in each time interval^2,17^  Interval can be defined as:   - Quarter/3 months^2,17^ - 6 months ^11,22^ - 12 months^22^ | Visit level measure^11^  Reflects a specified time period and can be cross sectional or longitudinal^22^ | From routine patient record  Visit dates ^17,22^ | **Presentation:**   - Cross sectional: visits in a particular window - Longitudinal: visits in each of multiple consecutive windows ^22^   **Calculation:**  Proportion of six-month person periods with ≥1 HIV-specific visit ^11^  **Categorisation:**  Retained: ≥1 visit in every interval  Not retained: no visit in ≥1 interval^22^  Good retention: having ≥1 visit in all intervals  Moderate retention: having ≥1 visit in ≥50% of intervals  Poor retention: having ≥1 visit in <50% intervals^17^ | Can demonstrate pattern of retention and engagement type of patients who are retained in care by summary patient-level measures like fixed time points or loss to follow up Visit level measure ^11^ |  | Visit constancy was associated with viral failure (VL>1000 copies/mL):   - 6 month constancy: OR 11.9 [6.9-20.0]^22^ - 12 month constancy: OR 8.2 [4.8-13.8]^22^   Shorter intervals had higher sensitivity (83% for 6 months vs 42% for 12 months) and NPV (92% for 6 months vs 92% for 12 months)^22^  Quarterly visit constancy (53%^2^ and 72%^17^) produced a lower estimate of retention than number of isolated clinic visits, single time point retention and missed visits with a definition of 60 days (63%^2^) but higher than missed visits by 14 (30%^2^) or 30 (38%^2^) days and gaps in care (43%^2^ and 50%^17^) | ^22^  ^17^  ^11^  ^2^ |
| The Health Resources and Services Administration HIV/AIDS Bureau (HRSA-HAB) | Two HIV-specific contacts separated by 90 days or more within each 12 month window^22^ | Longitudinal measure: presence of the definition of retention in each of the windows^22^ | From routine patient record  Visit dates ^22^ | **Categorisation:**  Retained: ≥2 HIV-specific contacts ≥90 days apart in each 12 month window ^2,22^  Not retained:  <2 visits or 2 visits separated by <90 days in each window | Has a high NPV^2,22^ |  | Developed by the Health Resources and Services Administration HIV/AIDS Bureau ^25^  Associated with viral failure: OR 9.5 [6.0-15.2]^22^  Produced higher estimates of retention (76%) than missed visits, visit constancy, single time point retention and gaps in care^2^ | ^22^  ^25^  ^2^ |
| Continually retained | This is a prospective definition of point retention - the first time a patient meets the definition of not retained, they are censored ^14,15^. It may be described as a cumulative probability of non-retention ^15^  The same windows or definitions of non-retention are used as above for fixed-point retention | Reflects continual unbroken retention over a specific period or the presence of any attrition within that period. | From routine data  Date of evidence of a visit/ interaction with the health system^14^ | **Presentation:**   - Retention - Attrition (1 - retention^14^)   **Categorisation:**  Retained; does not meet the definition of non-retention by the end of the observation period  Attrition/ not retained/ lost to follow up: meets the definition of non-retention within the study period | Does not have a differential classification of patients as not retained depending on the timing relative to analysis^15^ | Estimates a higher rate of non-retention as it assumes any transient interruption is lost to follow up (misclassification) ^15^ |  | ^14^  ^15^ |
| Inter-agency Task Team (IATT) definition | Alive and in care at 1, 2, 3, (early retention) and 6, 9 and 12 months post initiation (measures regularity and continuity of attendance) |  | From routine patient records  Visit date, scheduled visit date ^10^ | **Categorisation:**  Retained: Visit at each time point  Not retained: failure to attend ≥1 visit^10^ | Capture visit consistency along the continuum of care ^10^  Higher sensitivity (though still<10%) and lower misclassification than single time-point measures for ‘true’ retention (measured by a composite outcome of point retention and ≥75% appointments met on time)^10^ | More complex to calculate and require more data points to be captured - less feasible in busy clinics with low staff numbers ^10^  Data tools are not adapted to longitudinal follow up^10^  Patients may be seen in different departments and data needs to encompass this ^10^ | Suggested by the IATT in 2015^19^  Lower estimate of retention (16.4%) relative to point retention at 12 months^10^  IATT definition misclassified 15.9% of patients’ retention compared to a composite measure of point retention and ≥75% appointments met on time (with a 15 day window)^10^ | ^10^ |
| Visit pattern | Establishes and quantifies the patterns of attendance | Reflect a pattern of retention over a specified interval | From routine patient record  Visit dates | **Calculation:**  Use cluster analysis (dynamic time warping clustering method) to find patterns^3^  **Categorisation:**  Visits clustered into 2 main specific patterns:  - "Regular attendance" defined by regularity (attended ≥3 visits)  - "Non regular attendance" defined by frequency^3^ |  | To evaluate patterns in a population the follow up time needs to be comparable otherwise the patterns cluster around the follow up time rather than the frequency or regularity of visits^3^  Attendance measures assume that medication is taken and do not automatically imply adherence^3^ | Regular attendance more strongly associated with retention in care at 12 months than the non-regular attendance cluster: OR 3.14 [1.45-6.81]^3^ | ^3^ |
| Retention trajectory | Engagement is characterised by the pattern of different retentions states (in care, 3-6 months late for follow up, >6 months late for follow up, transferred out, died) ^26^ | Reflect a pattern of retention over a specified interval^26^ | From the routine patient record  Outcome: in care, 3-6 months late for follow up, >6 months late for follow up, transferred out, died^26^ | **Calculation:**  Probabilities of group membership estimated using a multinomial logistic model^26^  **Categorisation:**   1. Group 1 “remained” in care 2. Group 2 exited care then “returned” after a median of 4 [3–9] months 3. Group 3 “exited care rapidly” after a median of 4 [4–6] months 4. Group 4 “exited care later” after a median of 11 [9–13] months^26^ | Approach to analysing RIC is innovative: does not consider retention as a simple binary variable at a given point of time, rather a dynamic phenomenon where patients can cycle in and out of care, with multiple possible trajectories^26^ | Retention outcome susceptible to misclassification due to missing information (particularly on death or transfer)^26^ |  | ^26^ |
| Timing of retention | | | | | | | | |
| Late for visit | Describes the timing of a visit relative to the expected or scheduled appointment  Late visits can be defined as   - Visit >7, >14 days, >30 days or >60 days after the scheduled/ expected appointment^2,7^ - Cut-off can be defined using the median number of days late in the sample ^20^   Also used as a measure of adherence ^20^ | Can reflect a range of visits from once off to multiple consecutive visits ^21^ | From routine patient records  Date of clinic attendance and date of scheduled ^7,20^ or expected visit | **Presentation:**   - Binary outcome of presenting on time or late^21^ - Number of days late for the scheduled appointment^7^ - Cumulative number days ever presented late in a time period^7^ - A degree of lateness as the number of days late or a proportion of lateness^4^   **Calculation:**  Days late = visit date – expected/scheduled visit date^8^  Expected visit date = last visit + days of dispensed ART ^8^  Degree of lateness for appointment = 100% - (days late/total days between visits*100)^4^  **Categorisation:**  One visit:  On time pick up: ART refills within cut-off of the appointment date ^21^  Multiple visits:  Retention over all visits: on-time pick up at ≥90% visits  Non-retention: on time pick <90%^21^  Degree of lateness:  Attendance on schedule = 100% adherent  Late for appointment <100%  Missed visit = 0% adherent ^4^ | Less intrusive on patients than self-reported measures^21^  High specificity means that patients identified as non-retained are likely to non be retained and warrant intervention^20^  Can be performed more frequently than viral loads^20^ | Susceptible to missing data, which is greater when the interval between the scheduled and actual visit is greater^7^  Proxy measure of adherence^21^  Low sensitivity means these measures cannot replace viral load monitoring - an adherent result is fairly meaningless and should be regarded with scepticism ^20^ | Pharmacy refill pickup timing was associated with an unsuppressed VL (>400 copies/mL):   - On time vs late pickup: OR1.27 (1.16-1.38) - On time relative to median lateness OR 1.38 (1.24-1.53) - 30 day lateness OR 2.56 (2.16-3.03)^20^   Stronger association with a longer duration of ‘lateness’^20^  Being late for a visit was associated with a significant hazard of viraemia (>100 copies/mL)  ≥7 days: HR 1.80 (1.07-3.03)  >14 days: HR 1.74 (1.07-2.85) ^7^  Retention of ≥90% of on-time (within 7 days) ARV refills was associated with better HIV outcomes in patients on ART for ≥18 months: a better CD4 count response ((206 cells/dL [193.1-219.6]) than non-retained patients (186 cells/dL [179.4-193.7]) (p=0.0145)) and a reduction in the risk of opportunistic infections (significantly higher proportion of retained patients (81%) had no OIs at 12 months compared to non-retained patients (24%)) after 12 months treatment ^21^  14 day definition of a missed visit produced the highest PPV and lowest estimate of retention (30%) compared to longer definitions (30 days: 38%, 60 days: 63%), gaps in care, visit constancy and single time point retention^2^ | ^7^  ^21^  ^20^  ^4^ |
| Time to loss to follow up | Time to first episode of non-retention from enrolment | Reflects the time between initiation of ART or a starting point and the first episode of non-retention | From patient routine records  Visit date^11^ | **Presentation of time:**   - Days - Weeks - Months   Years. |  |  |  | ^11^ |
| Appointment intervals | Time between the visit date and the next scheduled appointment^1^ | Patient perspective: reflects time since ART initiation  Programme perspective: reflects time by a calendar date^1^ | From routine patient records  Visit dates, appointment dates^1^ | **Presentation**   - Interval between two visits - Average or median interval   Most frequent time interval between visits in a time period^3^ |  |  |  | ^1^  ^3^ |
| Composites of retention measures | | | | | | | | |
| Point retention and missed visits | Composite of   - Point retention (e.g. ≥335 days post-delivery)   And   - A specific proportion of visits are not missed (e.g. <25% of missed visits defined as >14 days of the scheduled appointment + no gap in care >90 days)^2^ | Reflects a pattern over a specified time period | From routine patient records  Visit dates, scheduled visits or estimated visits | **Categorisation:**  Engaged: both retained by the point retention definition and < specified proportion of missed visits |  |  | Had a higher estimate of retention (55%) than retention by missed visits (14 days definition: 30%, 30 days definition: 38%), gaps in care (43%), visit constancy (53%) and number isolated clinic visits (76%)^2^ | ^2^ |
| Point retention and average retention | Composite of   - Point retention (e.g. alive and in care at 12 months)   And   - A specific proportion of kept visits (e.g. with ≥75% appointments attended during follow up) ^10^   Also called ‘Appointment adherence’ ^10^ | Reflects a pattern over a specified time period | From routine patient records  Visit date, scheduled or expected visit date^10^ | **Calculation:**  Number of visits attended divided by the number of total scheduled/ expected visits during the observation period ^10^  **Categorisation:**  Retained: both retained by the point retention definition and with average appointment attendance ≥75%^10^. | Capture visit consistency along the continuum of care^10^  Higher sensitivity and lower misclassification than single time-point measures^10^  Very high specificity for retention^10^ | More complex to calculate and requires more data points to be captured - less feasible in busy clinics with low staff numbers^10^  Data tools are not adapted to longitudinal follow up^10^  Patients may be seen in different departments and data needs to encompass this ^10^ | Misclassified 18.3% patients’ retention compared to a composite measure of point retention and ≥75% appointments met on time (with a 15 day window)^10^ | ^10^ |
| On time attendance retention | Composite of   - Point retention (e.g. alive and in care at 12 months)   And   - Timing: a specific proportion of appointments on time (e,g, ≥75% appointments met on time (+- 15 days) during follow up) ^10^ | Reflects a pattern over a specified time period | From routine patient records  Visit date, scheduled or expected visit date^10^ | **Calculation:**  Number of visits attended on-time divided by the number of total scheduled/ expected visits ^10^  **Categorisation:**  Retained: both retained by the point retention definition and with on time appointment attendance ≥75%^10^. | Capture visit consistency along the continuum of care^10^  Stringent definition^10^  Measures delay in attendance^10^ | More complex to calculate and require more data points to be captured - less feasible in busy clinics with low staff numbers^10^  Data tools are not adapted to longitudinal follow up^10^  Patients may be seen in different departments and data needs to encompass this^10^  Too time consuming and incompatible with current service systems^10^ | Lowest proportion retained (1.2%) relative to point retention, WHO, IATT and Mozambican MoH definitions ^10^ | ^10^ |

## Table 7: Measures of adherence with detailed information on their use

| Measure | Description | | Data required | | Evaluation | | | Ref |
| --- | --- | --- | --- | --- | --- | --- | --- | --- |
|  | Definition | Pattern and duration of measurement | Data collected and source | Processing: presentation, scoring, calculation, categorisation and interpretation | Strengths | Limitations | Evidence (for association with clinical outcomes/ other elements of engagement/ validity) |  |
| PRIMARY ADHERENCE^28^ | | | | | | | | |
| Pharmacy refill | | | | | | | | |
| Pharmacy visit compliance/ crude pharmacy interval | Adherence is measured as the  collection of antiretroviral medications from the pharmacy  at prescribed intervals. It is assumed that if patients collect  their medication, then they are likely to take the medication^29^  Also called a ‘crude interval’ as it does not consider the pills dispensed, only the number of refills^30^  Can be seen as a retention measure | Can be used over various time periods  either for a standardised calendar time period or time since ART initiation^31^ | From the routine patient records  Pharmacy refill visit date: scheduled/ estimated date and actual date attended^20^ | **Presentation**  Adherence level is expressed as a percentage of the number of times they should have collected medication over the period ^29^  **Calculation:**  Pharmacy visit compliance = (#pharmacy refills in interval)/(#months in interval)*100 ^30^  **Categorisation:**  Dichotomised  Adherent: ≥95% pills taken ^31^  Non-adherent: <95%^31^  OR  High adherence =  had all scheduled refills in the period  Moderate adherence =  missed 1 refill  Low adherence =  missed ≥2 refills^32^ | Easily collected data, particularly from digitised records, from data that is collected routinely in any case^29^  Inexpensive method^29^  More objective than self-reportsc  Retrospective so are not constrained by collection of prospective data^29^  Interval crude method is easy to calculate and could be implemented in pharmacies with staff shortages or who use manual records^30^  High specificity means that patients identified as non-adherent are non-adherent and warrant intervention^20^  Can be performed more frequently than VL^20^ | Low sensitivity^20^ and the fact that pharmacy refill adherence misclassified a large proportion of patients with detectable viral loads as suppressed^29^. Thus pharmacy refill adherence is not recommended for monitoring ART alone (without CD4 and VL)^20^, but can be combined with other methods^29^  Overestimate adherence if patients discard or share pills - gives the maximum possible adherence^29^  Do not reflect the dynamic nature of adherence ^29^  Indirect method of evaluating adherence^28^  Requires longitudinal data recording^28^ | Odds of viral failure (>1000 copies/mL) were 14.4 times (3.8-54.8) greater for low and 2.6 times (0.3-24.8) times greater for moderate adherence compared to high adherence^32^ and associated with viraemia (<400 copies/mL) with a ROC AUC between 0.5 and 0.6 ^30^. However, another study found no statistically significant relationship between adherence by pharmacy refill and viral suppression (r = 0.094) with high rates of misclassification: 82% of patients achieved optimal suppression but 38% of these did not have viral suppression ^29^  Performed worse than MPR and medication gaps at predicting viral outcomes^30^  Pharmacy refill compliance over nine months and since ART initiation are correlated: proportion of agreement 76.4% Cohen's Kappa 0.311 (95% CI 0.186-0.436) ^31^  Correlated with four-day recall self-report (r=0.36), VAS (r=0.66) and CAS (r=0.72) ^32^ | ^31^  ^32^  ^29^  ^30^  ^20^  ^28^ |
| Medication possession ratio (MPR) | Evaluates pill coverage/availability accounting for accrued and unused medication^30^: proportion of time that a patient has medication on hand as a proxy for the maximum possible adherence^33^  Also called ‘pharmacy refill adherence’ and ‘interval average’ ^30^ or refill average^34^  Averages the adherence across the whole period^34^ | Average adherence over a specified interval (minimum of 2 visits^33^)  Intervals between visits vary according to programme and service guidelines^33^ | From the patient’s routine records^35,36^, pharmacy records or secondary database analysis ^37^  or the patient through interview^33^  Refill dates, number of pills for each ARV dispensed and prescribed ^4,30,33,36,38^  Also collected info on explanations for more or fewer pills - refills from elsewhere, clinician advice to pause treatment or ART sharing - adjust/explain estimates^33^ | **Calculation:**  MPR = (#days with pills)/ (#days in the period)*100^9,33,34^  #days with pills = #pills dispensed/#doses per day^9,33,34^  #days in period = last dispense date – schedules appointment^39^  OR  Interval AVERAGE = (#days of medication from last refill + accrued days of medication from previous refill in prior interval - unused days medication from last refill)/ (#days in interval)^30^  OR  MPR = 100 - percentage time without medication^35^  Percentage days without medication = cumulative sum of days patient was late for pick up each month/total period of time*100 ^4,35,38^  Decisions to be made:  1.Carry over extra pills from a previous period if on the same regimen^33^  2.Cap MPR at 100% for each period^4,30,33^  3. MPR for each drug separately or for all ARVs overall (if not on fixed dose) ^33^  **Categorisation:**  Continuous variable ranging from 0-100% ^9^  OR dichotomised  Adherent: 100%^20,36^, ≥95%^33,38,39^, ≥90%^20,40^ or ≥80%^20^ pills available  Non-adherent: <100%^20,36^, 95%^33,38,39^, <90%^20,40^ or <80%^20^  Or subcategorised as:  Optimal adherence: ≥95% refill adherence  Suboptimal adherence: 80-94% refill adherence  Poorly adherent: <80% refill adherence^35^  Only adherence rates above 95% were observed to lead to <10% virologic failure thus this is the recommended threshold to evaluate adherence^35^. | Pragmatic measure in resource-limited settings^34^ - good feasibility:   - Cost-saving^4^ - Can be performed more frequently than VL^20^ - Cheaper than pharmacokinetic measures to predict need for VL^30^ - Not intrusive on patients or providers^39^   ART programmes generally have patients returning to the same facility on a regular basis for ART refills so have the data to calculate this metric already (easier if computerised)^4^  High specificity means that patients identified as non-adherent are non-adherent and warrant intervention ^20^  There is a dose response relationship between virological outcome and grouped pharmacy refill adherence^4^  Software to calculate these metrics could easily be added to existing electronic dispensing systems, which are already widely used in resource-limited settings^34^  Non-invasiv^28^ | Data abstraction, cleaning and calculation take time and effort, particularly if records are not electronic - decrease feasibility in practice  Interval average may overestimate adherence because patients receive more treatment (30 days) than the gap between refills (28 days) so the accrued pills are included in the numerator, reducing days of missed medication. Overestimation of adherence issues may lead to unnecessary clinical interventions^30^  Gives maximum possible adherence rather than pill-taking behaviour^36,40^ - proxy rather than a direct measure of adherence - assumes that the dispensed drugs are consumed which may not be the case ^4,20,28,39^ and it does not account for pill dumping or sharing^28^  Many false negatives due to low sensitivity ^4^  Assumes all refills are obtained from the same facility^4^  Depends on the quality of record keeping in the clinic^40^ and requires consistent access to all pharmacy data, significant back-end programming for calculations, and a closed pharmacy system^28^  Other causes of unsuppressed VL (resistance or issues with drug absorption or dosing) are not differentiated by pharmacy measures ^20^  Does not identify patterns of adherence (e.g. sustained adherence, cumulative adherence or treatment interruption) which may be more risky for developing resistance^4,20,28^  Evaluation of adherence over shorter periods is unstable and less accurate than longer periods^20,28,34^ | Associated with VL suppression (<400 copies/mL): ROC AUC 0.6 across 3-12 months ^30^ but non-adherence associated more strongly with VL ≥400 copies/mL than ≥40 copies/mL and with VL>1000 copies than >400 copies/mL^4^: better predicts viral failure than viral blips^33^  Associated as a continuous variable with viral failure 48 weeks after ART initiation: AUC 0.73 [0.61 to 0.85] and adjOR 0.78 (0.69-0.87) ^34^  Pill coverage associated with VL as a continuous variable (dose response relationship) ^4^ and as categories with viral failure (VL >1000 copies/mL) and viraemia (VL >40 or >400 copies/mL) varies by threshold with a greater strength of association with lower threshold of adherence^20^   - <100% coverage: OR 1.47 [1.02-2.15] for viral failure^36^, OR 1.26 [1.15-1.39] for VL>400 Sensitivity 14% [12-15] Specificity 89% [88-89], NPV 85% [84-86] ^20^ and OR 0.84 [0.39-1.77] for VL>40^33^. - **>95% coverage:** OR 3.25 [1.86-5.68] for VL>40^33^ and was associated with virologic suppression (adjOR 1.61 [1.15-2.27]^39^ and OR 2.92 [1.63–5.22] compared to adherence <80%^35^). The best threshold for predicting viraemia (>400 copies/mL) was 95% ROC AUC: 0.61 sens 60% spec 62%, AUC 0.63 and sensitivity 69% (specificity 57%) adjusted for CD4 ^4^ and only adherence rates above 95% were observed to lead to <10% virologic failure^35^ - <90% coverage OR 1.71 [1.50-1.96] for VL>400 Sensitivity 7% [6-8], Specificity 96% [95-96], NPV 85% [85-86] ^20^ - <80% coverage OR 1.89 [1.62-2.20] for VL >400 Sensitivity 6% [5-6], Specificity 97% [97-97], NPV 85% [85-86] ^20^ and OR 4.94 [2.52-9.69] for VL>40 Sensitivity 25%. NPV 92%^33^   The metric predicted failure and resistance at 48 better than 16 weeks - suggested that cumulative evaluation of adherence over longer periods is more accurate^34^  Associated as a continuous variable with resistance 48 weeks after ART initiation: AUC 0.83 [0.65 to 1.0] and adjOR 0.77 [0.66-0.89]^34^  Pharmacy refill was associated with imunological failure (CD4<100 or lower than preART value): adjOR 1.92 [1.2-2.3]^39^, time to CD4 recovery ^4^ and sensitivity for VL was increased with CD4 in the model ^4^  Better sensitivity and NPV than self-report (by AACTG) and clinician-assessment of adherence ^33^ and stronger association with viral suppression than self-reported single item adherence ^20^. Pharmacy refill with a threshold of 95% outperformed self-report, pill count and appointment keeping adherence at evaluating adherence relative to VL outcomes, independent of other variables ^4^  Produced higher estimates of adherence than other self-reported measures (VAS, CASE Index, AACTG, 30 day recall and 48 hour missed pills) and healthcare provider estimates of adherence ^36^ but identified more non adherent patients than self-report (lower estimate of adherence 82% than self-report: 90%)^38^  Not significantly associated with self-management, except the item: only 'attends themselves' was significantly associated: those who attended themselves had a 2.23 greater odds of poor adherence by MPR [1.32-4.14] than those who attended with a caregiver ^40^ | ^33^  ^35^  ^36^  ^38^  ^30^  ^4^  ^37^  ^40^  ^20^  ^9^  ^39^  ^28^  ^41^ |
| Poor adherence proportion | Quantifies the number of visits with a low adherence measured by MPR for that interval | Reflects a specified interval | From routine patient records  Date of dispensing and visit, number dispensed. | **Calculation:**  Continuous outcome  Adherence = (# individual visits with <95% adherence)/(#visits)*100^42^  **Categorisation:**  Good adherence: ≥95% adherence  Poor adherence: <95% adherence^42^ |  |  | Associated with virologic failure (VL>400 copies/mL x2 or x1 then LTFU): ROC AUC 0.69 [0.61 to 0.76]^42^  Predicted virologic failure better than sustained poor adherence by refill but did not perform as well as electronic adherence monitoring ^42^ | ^42^ |
| Sustained poor adherence | Patients categorised as adherent or non-adherent depending on presence or absence of '≥2 consecutive individual refills with <95% adherence’^42^ | Reflects a specified interval | From routine patient records  Date of dispensing and visit, number dispensed. | **Categorisation:**  Refill adherence = <2 individual refills with <95% adherence  Refill non-adherence = ≥2 consecutive individual refills with <95% adherence^42^ |  |  | Associated with virologic failure (VL>400 copies/mL x2 or x1 then LTFU): ROC AUC 0.58 (95% CI 0.50 to 0.66) ^42^  More weakly associated with viral failure than the proportion of visits with poor adherence by refill, by electronic adherence monitoring and by CD4 ^42^ | ^42^ |
| Pharmacy refill gaps | Adherence based on medication-free days where patient could not have medication in hand and it would be impossible for a patient to have taken medication ^34^  Also called an “Interval gap” ^30^ | Reflects cumulative adherence for the interval between visits ^34^  Minimal interval tends to be one month ^34^ | From routine patient records  Date of dispensing and visit, number dispensed. ^30,34^ | **Calculation:**  MPRgaps = (#days in period - medication-free days)/(#days in the period)*100^30,34^  #days = date visit - date dispensed  medication-free days = #days in period - (#pills dispensed/#doses/day) ^34^  **Categorisation:**  Good adherence: >80% days with treatment  Poor adherence: <80% days with treatment^30^ | Pragmatic measure in resource-limited settings^34^  Software to calculate these metrics could easily be added to existing electronic dispensing systems, which are already widely used in resource-limited settings^34^  Interval gaps more accurately account for missed medication days than interval average^30^, and expected to yield lower but more accurate median adherence than average adherence by pharmacy refill^34^  Interval gaps performed consistently regardless of the duration of the interval ^30^ | Interval gap calculation is more complicated and difficult for use in busy clinics with staff shortages^30^ | Associated with viral failure 48 weeks after ART initiation: AUC 0.72 (95% CI 0.59 to 0.84) and adjOR 0.68 (0.56-0.82) ^34^ and with unsuppressed VL (>400 copies/mL) >0.6 across 3-12 months ^30^  Association with VL varied with interval and threshold   - The threshold of 80% performed best on ROC to predict VL suppression^30^ - Greater association with failure and resistance at 48 than 16 weeks (viral failure: adjOR 0.64 (0.47-0.88)) - suggested that cumulative evaluation of adherence over longer periods is more accurate^34^. 4 month interval gap pharmacy refill was selected as the optimum and most pragmatic measure of adherence, and had a 'dose response' relationship with VL suppression^30^. - Gaps outperformed average MPR and pharmacy visit adherence at predicting VL outcomes^30^   Associated with resistance 48 weeks after ART initiation: AUC 0.86 (95% CI 0.67 to 1.0)  adjOR 0.77 (0.66-0.89) ^34^  More stringent: produced lower estimates of adherence than average adherence by pharmacy refill, but this is interpreted as being more accurate^34^ | ^34^  ^30^ |
| SECONDARY ADHERENCE^28^ | | | | | | | | |
| Antiretroviral concentrations | | | | | | | | |
| Blood levels | Measures adherence by evaluating exposure to individual or combinations of ARVs in the patient’s plasma | Reflects short term adherence over the previous few days^34^, specifically ± 3 days ^43^or 4-5 days^44^ depending on the ARV. Longer periods reflected for efavirenz (long half-life^45^) | From patient  Self-reported time of dose ^34,44^  and  Blood sample by clinician:   - Whole blood collected and EDTA tube via venepuncture ^44,45^   or remnant after resistance testing ^46^   - Dried blood spot collected via finger prick^46^   Mid dosing interval: patient blood sample drawn in the morning after an evening dose/ 12-18 hours after the last dose^34,44^ | **Categorisation:**  Dichotomised  Qualitative categorisation:  Exposed: ≥1^46^ or ≥2^47^ ARVs detectable (≥ lower limit of detection^46^)  Not exposed: <1^46^ or <2^47^ ARVs detectable or none detectable/ 1 detectable/ 2 detectable ARVs other than ART regimen^43^   - Efavirenz: undetectable <0.02ug/mL^43^ or ≤100ug/mL^48^     Or  Adherent: ≥therapeutic concentration (between lower and upper limit of therapeutic range) or ≥2.5th percentile of published population levels^45^ or thresholds that maximise sensitivity and specificity (ROC)^44^  Non=adherent: sub-therapeutic concentration (<lower clinical cut-off^49^ or minimum inhibitory concentration (Cmin)^50^) or <2.5th percentile of published population levels^45^  - Lamivudine = 0.1mg/L^50^  - Nelfinavir = 0.7 mg/L^50^  - Efavirenz: therapeutic range for efavirenz concentrations is 1-4mg/L^34^ or ≥1.13 (max ROC)^44^  - Nevirapine concentrations of 3.9mg/L^50^ or 3.0 μg/ml may be an optimal threshold on the basis of steady-state trough concentration reached in the pharmacokinetic curve for nevirapine following a 200mg twice daily dose ^51^   - Plasma tenofovir: ≥23.5 (max ROC)^44^ - Higher cut offs for optimal performance are needed in Africa - likely due to higher proportion of slow metabolisers^49^   Or  Subcategorised  Adherent: ≥Cmin at all times  Once-off non-adherent: <Cmin once  Repeatedly non-adherent: <Cmin >once^50^ | Objective and direct measure of adherence ^46^ that is less affected by patient behaviour than self-report and pill counts^51^  Can be done with plasma or dried blood samples, and the same sample can be used for the ARV level then the genotype resistance testing^46^  Measures last dose taken^52^  It can help direct interventions to adherence vs biomedical reasons for low concentrations ^49^  PI-exposure can determine eligibility for expensive resistance testing and make effective use of limited resources - differentiate between different underlying causes of viral failure (adherence vs resistance)^46^  Those misclassified by drug exposure testing as non-adherent (vs intermittently adherent or resistant) would still benefit from adherence optimisation before genotypic resistance testing to ensure that the genotype identifies all possible mutations and that third line treatment is optimised appropriately^46^  The ARV plasma levels detected more non-adherence and indicate early non adherence, whereas the VL detected less non adherence and indicates late non-adherence ^48^ | Medication specific validation required^52^  Short term adherence (about 4-5 days) ^44^  Affected by   - Body weight (decreased weight increases concentration in hair likely due to supratherapeutic plasma concentrations)^53^ - Genetics and metabolism ^51^ which are fairly evenly split in the population ^54^, particularly efavirenz (dependant on CYP2B6 metabolism^44^) making interpretation complicated - Diet, absorption, and rate of excretion^28^   Poor feasibility:   - Expensive^44,49,52^ - Requires lab capacity and complex equipment^44^ - Not generally available, particularly in resource-limited settings^44,49,52^ - Require knowledge of timing of drug ingestion, making interpretation of low concentrations difficult unless this information is collected^34,49^ - Requires complicated logistics and storage (-80C)^34,44^ - Requires complex calculation^34^ - Dried blood spots are unrealistic for routine evaluation of adherence due to cost and delays in results^43^   Provides only recent dose information^28,52^ and vulnerable to white coat adherence falsely elevating concentrations^34^ - risks overestimation^52^. Variations in metabolism and medicine taking behaviour around visits can give a false impression of adherence^28^  Serum concentrations may not reflect intracellular concentration of the active triphosphates of the nucleoside analogues thus can misclassify the actual adherence^52^  Can misclassify adherence by   - Overestimation of adherence if sample not taken at nadir of concentration^48^ - Categorisation by 'detectable' vs 'non detectable' threshold^48^ can overestimate adherence if quantification of drug level is not used as drug may be present but subtherapeutic^43^ - Misclassification of patients as non-adherent but who had resistance, may be due to intermittent adherence ^46^   Plasma tenofovir performed even worse than efavirenz because the half-life is likely shorter due to a lack of effect of CYP^44^ | Mixed evidence of association with viral outcomes: ART levels(efavirenz, nevirapine, lopinavir and atazanavir) had some agreement with virologic failure (VL>1000 copies/mL): kappa 0.25 and 0.36 (at visit 1 and 2)^45^, plasma ARV concentrations are lower in those with failure than those without^49^ with had good specificity (90%) but poor sensitivity (38%) and sub-therapeutic ARV concentrations explained only 38.2% of viral failure^49^. Repeated subtherapeutic levels (of lamivudine, nevirapine or nelfinavir) were associated with sustained viraemia (absence persistent VL suppression <400 copies/mL x 3) adj RR of 0.43 [0.19-0.95] but a single episode of subtherapeutic levels was not significantly predictive of VL outcomes (adj RR 0.86 [0.66-1.11] compared to adherence:≥Cmin)^50^  Blood ARV concentration was not associated with CD4 trend ^50^  No relationship between self-reported adherence and presence of ARV medication^55^ and produced lower (more stringent) estimates of adherence than self-reports (74% vs 89% by VAS and 80% by ACTG) ^43^ and 60% vs self-report (90%) and pill count (74%) ^50^ but lower estimates of non-adherence in another study (7% vs 17% for a pictogram-enhanced VAS) ^45^  **Tenofovir**   - Tenofovir ≥23.5 ng/mL predicted viral suppression (<50 copies/mL): ROC adjAUC 0.864 [0.797 - 0.932]. This was significantly poorer than DBS TFV-DP (AUC 0.926) ^44^ - Higher drug cut offs increased the PPV and specificity ^44^   **Efavirenz**   - Mixed evidence from qualitative exposure categorisation being highly predictive of suppression with sensitivities approaching 100% and correct identification of 89% of viral suppressions for plasma efavirenz^44^, or moderate specificity (89%) and low sensitivity (42%) for viral failure^49^ to no association with virological failure at 48 weeks after ART initiation^34^ - Association depends on threshold   - Higher drug cut offs increased the PPV and specificity^44^   - Efavirenz: 2.7mg/L optimised the ROC AUC (0.61) but had low sensitivity (64%) and specificity (54%)^49^   - Efavirenz ≥1.13 ng/mL predicted VL suppression (<50 copies/mL): ROC adjAUC 0.903 [0.839 to 0.967]. This was not significantly different from DBS TFV-DP (AUC 0.926)^44^   **Nevirapine**   - Associated with viral failure with high specificity (94%) and low sensitivity (28%)^49^ - Association depends on threshold   - Nevirapine: 7.8mg/L optimised the ROC AUC (0.71) but had moderate sensitivity (78%) and low specificity (56%)^49^   - Achieving a concentration of ≥3 ug/mL at 4 hours post ingestion was associated with virological success (VL <1000 copies/mL) and a lower mean VL (not found at 1 and 24 hours after dose)^51^ - Mean nevirapine was twice as high in good or fair self-reported or pill count adherence ( 3 392 - 3 344 ng/mL for good and 3 368-3 433 ng/mL for fair) as in poor adherence (1 278-1 638 ng/mL). Achieving a NVP concentration of ≥3 ug/mL at 4 hours post ingestion was associated with good adherence (not found at 1 and 24 hours after dose) ^51^   **Protease inhibitors**   - Identified approximately half of genotypes conducted in response to VL monitoring in second-line as unnecessary^46^.   **Lopinavir**   - Mixed evidence with level inversely associated with VL (those with detectable ARVs had lower VL than those undetectable ARV levels)^46^ but mean concentration no different in unsuppressed vs virally suppressed patients, and associated with viral suppression with moderate specificity (83%) and low sensitivity (44%)^49^ - The sensitivity depended on the blood sample and the test used:   - DBS: sensitivity 100% [54-100] and NPV 100% [88-100]   - Plasma: EIA (enzyme immunoassay (EIA)): Sensitivity 88% [82-93] and NPV 93% [89-96]/ LCMS (liquid chromatography-tandem mass spectrometry): Sensitivity 89% [83-94] and NPV 94% [90-97] ^46^ - An undetectable lopinavir level excluded the presence of PI-resistant viral strains with a high degree of certainty ^46^, and detectable levels had high sensitivity (90% [84-94]) and NPV (95% [91-97]) for the presence of major ARV resistance mutations in those with a raised VL. Optimal sensitivity was found at a level of 0.01mg/L (Lowest limit of detection - low level threshold beneficial for a screening test). 1mg/L (minimum trough level) has similar ROC AU with moderate sensitivity (79%) for LPV/r drug resistance of 79%. ^46^   **Atazanavir:**   - Associated with viral suppression with moderate specificity (86%) and low sensitivity (53%)^49^ | ^52^  ^34^  ^43^  ^49^  ^45^  ^46^  ^48^  ^51^  ^50^  ^44^  ^47^  ^53^  ^28^ |
| Intracellular levels | Measures adherence by evaluating exposure to tenofovir diphosphate (TFV-DP), an intracellular metabolite of tenofovir^56^ | Reflects the preceding 2 weeks^56^ or +- 17 days^44^ | From patient  Dried blood spot collected via finger prick ^43,44^ | **Categorisation:**  Dichotomised  Qualitative categorisation:  Exposed: ≥ lower limit of detection^44^  Not exposed: < lower limit of detection^44^  Or  Adherent: tenofovir diphosphate ≥399 ng/mL (threshold maximised sensitivity and specificity (ROC))^44^  Non-adherent: <399 ng/mL^44^ | Assesses long term adherence (half-life about 17 days) ^44^  Can be conducted at the point of care^56^  Can be used to identify white coat adherence (good short term adherence before a health service visit but poorer longer term adherence) when compared to plasma tenofovir: comparison of plasma Tenofovir (short term) and DBS TFV-DP (long term) - if plasma tenofovir higher than DBS, patient took ART better before visit ^44^ | Dried blood spot concentrations not feasible in routine settings:  - Dried blood spot TFV-DP more expensive than plasma concentrations ^44^  - complex logistics of transport and storage (-80C storage)^44^  - complexity of laboratory assays required and lack of lab capacity^44^  TFV-DP concentrations affected by:   - Gender: lower in men than women - Racial differences: lower in black patients than white/Hispanic patients^44^ - Potential differences in thresholds and concentrations in pregnant women ^44^ | TFV-DP exposure was highly predictive of suppression (VL<50 copies/ml) with sensitivities approaching 100% and correct identification of 89% of viral suppressions^44^ and there was a dose response relationship between increasing concentrations of TFV-DP and viral suppression (VL<50 copies/ml):  Adj OR with 95% CI for:  <350 (<2 pills per week): reference  350-699 (2-3 pills per week); 37 (8 to 178)  700-1249 (4-6 pills per week): 47 (13 to 175)  ≥1250 (7 pills per week): 175 (20 to 1539) ^44^  Higher drug cut offs increased the PPV and specificity^44^  TFV-DP ≥399 ng/mL had the highest (compared to plasma ARV concentration and self-report) adjusted AUC (0.926 [0.876 to 0.976] to predict viral suppression^44^ | ^43^  ^44^  ^56^  ^41^ |
| Hair levels | Measures adherence by evaluating exposure to ARVs in the patient’s hair | Hair grows at about 1cm per month so a 1cm sample from the base of the hair will give the previous month’s adherence^54,57,58^ ^57^ | From patient^54^  Hair sample: 20-30 strands 1cm ^54^ from base of skull (most proximal part) ^57–59^ | **Calculation:**  Added the lower limit of detection (0.002ng/mg for tenofovir) to all values to reduce potential for undue influence from differences between very small values^58^  Log transformed concentrations for analysis^58^  Adjustment for tenofovir and emtricitabine: if hair sample included any portion unexposed ARV, overall concentration is adjusted =  hair growth period (centimetres*30 days)/days of growth with exposure to ARV (assumed to start 6 days after initiation to allow TFV levels to build up in the systemic circulation and hair to grow out of the scalp)^58^.  **Categorisation:**  Efavirenz  Lower limit of detection: 0.625ng/mg  Upper limit: 40ng/mg ^54^  Best threshold to detect viraemia: 1.04ng/mg^59^ | Hair concentrations give a direct, quantitative ^60^, objective^57^, biomarker measure^57^ of cumulative ARV exposure^60^ that reflects long term^57^ adherence  Reflects drug exposure^61^ and integrates the effects of long-term adherence and steady-state pharmacokinetics ^53,60^  Whereas single plasma drug levels record exposure over the prior 24–48 h and may vary significantly from day-to-day, antiretroviral hair concentrations reflect uptake from the systemic circulation over weeks to months^60,61^  Hair sampling and testing was feasible^57^:   - Hair sampling was inexpensive^59,62^, easy^59,62^ (required minimal training and no specialised equipment^57,60^), was non-invasive^60^ and acceptable (59% to 95% participants donated hair) ^54,57,60^ - Transport and storage of hair samples was cheap^57^ and can be done at room temperature^60^ - Cost less than VL ($30 vs $50, option of $5 for a different test) ^57^ - Cheaper tests being developed^59,62^   Reflects ARV exposure while being independent of resistance (uptake in hair is not affected by the presence of resistance)^61^ so can help discriminate reasons for VL failure and identify those that are non-adherent over those that have resistance^57^  Antiretroviral concentrations in hair provide a longer window (previous month) of drug exposure and are less susceptible to transient improvements in adherence prior to visit than plasma measures, making it potentially more valuable than short term measures that are susceptible to white coat adherence ^54,60^ | Challenges experienced in collecting hair samples suggest that this adherence measure may have limited utility in an African population ^54^.   - Short hairstyles and weaves are common and make collection difficult^57^ - Many women were reluctant to donate a braid and many men had shaved heads^54^ - Patients non-confrontationally avoided sampling by cutting their hair before the appointment^57^ - Potential stigma re: witchcraft in hair collection - requires community engagement and active management^57^ - If hair is <1cm a repeat appointment needs to be made in 2-4 weeks to allow growth^57^   Concentrations affected by   - Body weight (decreased weight increases concentration in hair likely due to supratherapeutic plasma concentrations) ^53^ - Metaboliser type, which are fairly evenly split in the population ^54^ | The evidence is medication-specific  **Tenofovir**   - Higher hair concentrations were associated with VL suppression (<1000 copies/mL) on univariate analysis (Regression coefficient 0.5 [0.3-1.0] but not in multivariate analysis, and were associated with a greater decrease in VL from baseline (regression coefficient 0.8 on univariate and multivariate regression) ^61^. Each doubling of tenofovir concentration (log2 transformation) increased the odds of concurrent viral suppression (VL<400 copies/mL) (adjOR 2.35 [1.44-3.84]) and the odds of future suppression (adjOR 1.43 [0.75-2.73], not significant though sample with small number of events of failure)^58^ - The median hair tenofovir level in suppressed patients (0.54ng/mg [IQR 0.033-0.086]) was twice that in unsuppressed patients (0.026ng/mg [IQR 0.0007-0.041]) ^58^ - Not associated with VAS self-reported adherence^61^   **Efavirenz**   - Mixed evidence on relationship with virological failure from no relationship^54^ to aOR 1.86 [1.14-3.1] and 1.81 [1.22-2.7] (per doubling in concentration at delivery and 24 weeks post-partum)^60^, and association with viraemia (>400 copies/mL): ROC AUC 0.85 [0.64-1.00]^59^ - Best threshold: 1.04ng/mg with high sensitivity (98%) and moderate specificity (67%)^59^ - Strong correlation with efavirenz concentration in plasma samples (Spearman’s correlation coefficients, 0.672–0.741, p < 0.0001)^54^. - No relationship with adherence measured using an electronic adherence^54^.   **Nevirapine**   - Associated with viraemia (>400 copies/mL): ROC AUC 0.77 [0.57-0.98] ^59^ with good sensitivity (94%) and moderate specificity (62%) ^59^ - Non-significant, weak relationship with self-report in univariate and multivariate models^57^   **Lopinavir:**   - Each doubling in concentration increased viral suppression: aOR 1.90 [1.33-2.7] and 1.53 [1.05-2.2] (at delivery and 24 weeks post-partum) ^60^ and associated with viraemia (>400 copies/mL): ROC AUC 0.94 [0.86-1.00] ^59^ - Best threshold: 1.8ng/mg with very high sensitivity (100%) and moderate specificity (81%)^59^   **Atazanavir**   - Insufficient atazanavir concentrations (≤2.35ng/mg) associated with viral failure (VL>1000 copies/mL): adjRR 7.2 [1-51] (significant but imprecise), and a threshold of 2.35ng/mg was able to discriminate between viral suppression (associated with >2.35ng/mg) and failure (associated with <2.35ng/mg) with little overlap ^63^ - Hair concentrations were approximately 16% of steady-state plasma trough levels^53^. - Weakly associated with better self-reported (VAS/ACTG) adherence^63^, and a unit increase in self-reported adherence increased atazanavir concentration by 2% ^53^. | ^54^  ^57^  ^60^  ^58^  ^53^  ^61^  ^63^  ^59^ |
| Urine levels | Measures the level of the medicine urine^28^ | Reflects drug exposure over the previous 120 hours^41^ | From the patient through urine sample | **Categorisation:**  Positive: ARV detected  Negative: no ARV detected | Direct ^28^  Sensitive^28^  Point of care options: UTRA, a lateral flow assay, that takes 2-3 minutes to develop^41^  High specificity for poor adherence^41^  The combination of detected tenofovir in urine and viral failure was 100% sensitive in predicting dual class resistance on a low genetic barrier, efavirenz-based regimen. Therefore, the combination of UTRA and viral load testing could be used to target follow-up resistance testing or to prioritize transition to second-line regimens^41^ | Poor feasibility   - Expensive^28^ - Not practical for routine use ^28^ - Only a test for tenofovir reported^41^   Susceptible to misclassification of those with viral failure^41^  Variations in metabolism and medicine taking behaviour around visits can give a false impression of adherence^28^   - Findings can be misleading and are influenced by a variety of individual factors, including diet, absorption, and rate of excretion^28^ - Only reflects recent medication consumption^28^ and the short duration of exposure makes the test susceptible to social desirability bias and ‘white coat adherence’^41^ | The combination of positive urine tenofovir VL>1000 copies/mL was 100% sensitive in predicting dual class resistance on a low genetic barrier, efavirenz-based regimen^41^  Only 2 of 48 (4%) participants had undetectable UTRA tests (despite 38% experiencing virologic failure)^41^  Both cases with negative UTRA tests had either very low or undetectable TNF-DP levels in DBS (showing high specificity for poor adherence) ^41^  Misclassification of adherence with intracellular tenofovir diphosphate as the gold standard; one participant with positive urine for tenofovir had undetectable intracellular tenofovir diphosphate, and another 5 with positive urine had low tenofovir diphosphate levels which were at least one standard deviation below the mean (<173 fmol/punch) ^41^ | ^28^  ^41^ |
| Saliva levels | Measures adherence by evaluating the exposure to ARVs in the patient’s saliva | Unclear what period it reflects | From the patient   1. Stimulated saliva sample   Taken with a dental cotton roll (impregnated with 20mg citric acid to stimulate saliva) placed in mouth for the patient to chew on for 1 minute, then stored at room temperature (stable for 17 hours at ambient temperature of 23C) ^64^  Saliva collected from the cotton ball by centrifugation and stored at -80C^64^   1. Self-reported date and time of most recent ARV ingestion^64^ | **Categorisation:**  Therapeutic concentration: > 1.75mg/L^64^ | Saliva is a rapid and non-invasive sample^64^  Saliva concentration is independent of the time after ingestion^64^  Uses a simple and inexpensive laboratory test (thin layer chromatography, vs high-performance liquid chromatography, which is expensive) ^64^  ARV concentration is a direct measure of adherence^64^ | Thin layer chromatography is a semiquantitative measure and cannot calculate the exact concentration^64^  Zidovudine may confound thin layer chromatography results for nevirapine (the zidovudine has a retardation factor similar to the nevirapine reference standard) ^64^  Only evidence for nevirapine in this review – no longer a first-choice ARV | Nevirapine concentrations in saliva validated by L'homme et al in 2008^65^  Associated with other measures of adherence   - Nevirapine salivary concentration half that in plasma - median ratio of saliva: plasma concentration was 0.51^65^ - Good agreement with self-reported adherence: 91.9% of those with high self-reported adherence had therapeutic saliva NVP concentrations. The association was close to statistical significance (p=0.057) - Patients with high self-reported adherence were 87% less likely to have subtherapeutic salivary nevirapine compared to low self-reported adherence (OR 0.13 [0.02-0.98])^64^ | ^64^ |
| ARV detection of intermittent engagement | Measures the difference between continuous adherence and non-continuous (intermittent or white coat) adherence using short term measures (plasma) and longer term measures (intracellular or hair^54^) of ART concentrations | Reflects the longer adherence components: 2-4 weeks | From the patient  Blood for plasma concentrations  Dried blood spot for intracellular TFV-DP | **Categorisation:**  Continuously adherent: adherent by both  Intermittently adherent: adherent by TFV-DP^44^ or hair concentration^54^ but not by plasma concentration  White coat adherence: adherent by plasma concentration but not by TFV-DP^44^ or hair^54^ concentration  Non adherent: non-adherent by both |  | Can only detect intermittent engagement just prior to the blood level – full adherence does not reflect full adherence over the whole period |  | ^54^  ^46^  ^44^ |
| Laboratory tests | | | | | | | | |
| Haemoglobin | Haemoglobin as a proxy measure of treatment success |  | From the patient  Blood sample for haemoglobin |  |  |  | Point haemoglobin <13g/dL predicted viral failure: OR 2.76 [1.70-4.50]^66^ | ^66^ |
| Mean cellular volume (MCV) difference | MCV as a measure of adherence to zidovudine (Zidovudine causes an increase in the MCV within 4 weeks of initiation) ^67^ | Unclear but seems to reflect period since 4 weeks from initiation | From the patient  Blood sample for MCV | **Scoring:**  MCV at visit: Categorised as <80fL, 80-100fL and >100fL  **Calculation:**  MCV difference = MCV at 6 months - MCV at baseline  **Categorisation:**  MCV difference: dichotomised Adherent: ≥14.5fL^67^  Non-adherent: <14.5fL^67^ | MCV a good predictor in the most common regimen (TDF) ^67^  Has a high NPV - rule out ^67^ | Dependent on regimen ^67^  Higher sensitivity but very low specificity in TDF regimens | NPV was higher for AZT regimens ^67^  MCV at 6 months associated with viral suppression within 279 days from initiation with moderate to high sensitivity but low specificity  Association varied by regimen   - For TDF based patients: Sensitivity 97%. Specificity 3%. PPV 24%. NPV 78% - For AZT based patients: Sensitivity 70%. Specificity 61%. PPV 18%. NPV 94%^67^ | ^67^ |
| Bilirubin | An objective biomarker of adherence using bilirubin levels >1.3mg/dL as a proxy for exposure to atazanavir/ ritonavir ^68^, as atazanavir/ ritonavir causes a dose dependent raised unconjugated and total bilirubin by inhibiting the UGT1A1 enzyme ^68^ | Unclear, but remains stable for at least 2 years after initiating ATV/r ^68^ | From the patient  Blood sample | **Categorisation:**  Adherent: raised bilirubin: >1.3mg/dL  Non-adherent: normal bilirubin: <1.3 mg/dL (lab upper limit of normal) ^68^ | Cheap ^68^  Distinguish whether poor adherence is the cause of a high VL or likely to be resistance, thus could be used to direct management   - Focus genotyping to those with a raised VL AND a raised bilirubin - Focus adherence interventions on those with a normal bilirubin and raised VL^68^     High NPV ^68^ | Only applicable to atazanavir/ritonavir patients | Bilirubin levels <1.3mg/dL predicted virological failure in patients on second-line atazanavir/ritonavir (Odds of failure were 0.31 less for every unit increase in total bilirubin)and predicted viral failure better than pill count^68^  Sensitivity (66%) and specificity (72%) were moderate, with a very high NPV (95%) and a positive LR of 2.3/ negative LR of 0.5. Higher thresholds increased sensitivity but decreased specificity^68^. | ^68^ |
| Cathepsin | Quantification of cysteine cathepsins in peripheral mononuclear cells using multiplex cathepsin zymography as a measure of adherence to ART^69^ as patients on ART have been observed to lose cysteine cathepsin activity in their peripheral mononuclear cells, therefore their cathepsin levels decrease, particularly in patients on efavirenz and tenofovir^69^. | Unclear | From patients  Blood sample (EDTA) | **Categorisation:**  Good adherence: negative cathepsin means the cathepsin is suppressed so the patient is taking their ART  Poor adherence: positive cathepsin indicate poorer adherence | The assay to quantify active cathepsins is relatively inexpensive and makes use of technology platforms already present in laboratories around the world^69^  The assay is quick to run^69^  The assay is inexpensive relative to VL and drug concentrations^69^  The assay allows the cathepsins to be quantified^69^  High specificity^69^ | Some patients were cathepsin negative at baseline (not on ART) ^69^  Low sensitivity - many false negatives (14% of those with a suppressed VL were cathepsin positive) ^69^  Unclear of utility in non TDF/EFV regimens^69^  Unclear how long it takes to suppress cathepsin activity after starting ART^69^ | Multiplex cathepsin zymography is predictive of viraemia [VL>400 copies/mL]: adj RR 2.32 [1.26-4.29] ^69^ with high specificity (86%) but a low sensitivity (38%).  Positive LR: 2.66 (1.29-5.51) ^69^  Sensitivity and specificity increase in a stratified analysis of those that were cathepsin positive at baseline (excluding cathepsin negative, i.e. exposed to drugs or unlikely to change at baseline) ^69^  Similar sensitivity but better specificity than the SMAQ self-report^69^ | ^69^ |
| Resistance detection of intermittent engagement | Intermittent adherence indicated by PI resistance mutations as they persist for weeks to months compared to M184V (lamivudine and emtricitabine) mutations, which disappear quickly ^46^ |  | From the patient  Blood for genotype resistance test | Categorisation:  Intermittent adherence/ short term non-adherence: presence of PI resistance mutations and absence of M184V mutation^46^ |  |  |  | ^46^ |
| Healthcare worker measures | | | | | | | | |
| Clinician-recorded adherence | Routine provider assessment of patient medication taking at each follow up visit ^33^ | Can vary in time reflected from a month^36^ to previous six visits^33^ | From patient folder^33^ or from the healthcare provider through interview^36^   1. The proportion of time the participant had taken his/her drugs in the past month, based on: provider’s knowledge of the patient and their medical record   If >1 provider was interviewed: take the lowest estimate of adherence ^36^  OR   1. Clinician assessment of adherence as 'good', 'fair' or 'poor' based on   - Questions on number of ARV doses missed in the past month ^33^  - Clinician's judgement of adherence based on missed visits^33^ | **Presentation:**  Binary outcome  **Scoring:**  Clinician allocates the patient to a category according to the answers to their questions on missed doses and missed visits. ^33^  **Categorisation:**  Dichotomised  Perfect adherence = 100%  Non perfect adherence: <100% ^36^  OR  Sub-categorised as 'good', 'fair' or 'poor'^33^ |  | Relies on clinician entering data into the files accurately – susceptible to poor data quality^33^ | Significant association with viral failure (VL ≥1000 copies/mL): OR 1.57 [1.02-2.41] ^36^ and non-adherence associated with viraemia ≥40 and ≥400 copies/mL, with a stronger association with VL ≥400 copies/mL (better predicts viral failure than viral blips)^33^  ≥30 days gap independently predicted a detectable VL^33^  Weaker association with VL than MPR or 48 hour missed ART ^36^ | ^33^  ^36^ |
| Directly observed therapy (DOTs) | A health care worker directly observes a patient taking medicine | Reflects the adherence to the current dose |  |  | Most accurate measure compared to other measures of adherence^28^  Also has an effect on adherence itself | Patients can hide pills in mouth and discard them^28^  Not very practical for routine use^28^ |  | ^28^ |
| Pill counts | | | | | | | | |
| Electronic medication event monitoring system (MEMS) | Electronic device that notes when the box is opened^34^ to evaluate expected vs actual tablets taken in a dispensing period ^37^ | Reflects cumulative adherence over the measured interval, generally between visits^34^, generally the preceding 30 days ^70^ | Downloaded from the device, which must be brought in by the patient^34^  Number of 'adherent days': 24 hour periods (6am to 5h59am) where the box was opened ^34^  Date and time opened ^50^ | **Calculation:**  Adherence = #adherent days^34^/ # days in period^34^ (total prescribed doses^70^/ expected #openings^50^)*100^34^ ^71^  'adherent days': 24 hour periods (6am to 5h59am) where the box was opened^34^ (#observed doses^70^/openings^50^)  Decisions to be made:  Can make adjustments to the raw data downloaded from the electronic device:   1. By capping the data at a maximum daily adherence of 100% ^50^   Capped percent adherence = (observed doses ≤prescribed doses per day)/(total prescribed doses)*100^70^  Capping data creates a more conservative estimate with lower risk of misclassifying patients with borderline adherence and is the suggested method^70^   1. By adjusting it based on diary entries on non-prescribed MEMS use (if it was opened without taking a pill or a pill was taken without opening it)  - Add 'Inserted events' (if pill taken but cap not opened - Remove 'Excluded events' (if cap opened but pill not taken) ^70^   **Categorisation:**  Dichotomised  Good/optimal adherence: ≥95%  Poor/ suboptimal adherence:<95%^42,70^  OR  Sub-categorised as  ≥95% / 90-94.9% / <90% adherence ^50^ | Accurately records time and date of opening^52^  Provides continuous, real-time measurement^28^  Can detect VL rebound before established failure^34^ and can provide information on behaviour patterns^28^  Capping data creates a more conservative estimate with lower risk of misclassifying patients with borderline adherence and is the suggested method ^70^  Stringent method likely to produce more accurate estimates  Strong and consistent association with viral outcomes  Non-invasive^28^ | Does not record actual consumption^28^ and assumes a connection between opening and taking the medication - may overestimate true adherence^52^  May be unavailable in resource-limited settings^34^ as it is expensive^28^  Requires additional technology^34^ that is subject to mechanical malfunction^28^  Requires return visits and downloading data from medication vials^28^  Neither diary-cleaning, capping, nor cleaning and capping MEMS data significantly altered the association between adherence and VL - modifications are unnecessary, take a significant amount of time and are not worth the effort ^70^  Evaluation of adherence over longer periods is more accurate^34^ | Capped daily adherence was associated with viral failure (VL > 1000 copies/mL) 48 weeks after ART initiation: AUC 0.73 [95% CI 0.61 to 0.83] and adjOR 0.89 [0.82-0.95]^34^ and OR 19 [6-65] (correctly classified 73%)^70^ and with viraemia (VL >400 copies/mL x2 or x1 then LTFU): ROC AUC 0.91 [0.86 to 0.96]^42^. Considering adherence as a continuous variable, an increase of 1% adherence was associated with a 5% increased odds of an undetectable VL (OR 1.05) ^70^  Association with VL varied with interval and threshold, with increased the sensitivity, decreased the specificity and increased the proportion correctly classified with lower thresholds (e.g. 90% vs 90%)^70^   - Adherence <90% was significant in predicting sustained VL suppression (<400 copies/mL x 3) with adj RR 0.70 [0.50-0.98], 90-94.9% adherence was non-significant in predicting VL outcomes with adjRR 0.77 [0.57-1.02] compared to >95% adherence ^50^ - Adherence <95% associated with viraemia (>400 copies/mL): OR 27 [8-94] with moderate sensitivity (78%) and specificity (89%), and correctly classified 79% with VL outcomes, with a ROCU AUC 0.89 [0.82-0.95] ^70^ - Predicted viral failure and resistance at 48 better than 16 weeks - suggested that cumulative evaluation of adherence over longer periods is more accurate^34^   Associated with resistance at 48 weeks after ART initiation: AUC 0.92 [0.87 to 0.97] and adjOR 0.68 [0.53-0.88]^34^ but not associated with CD4 trend ^50^  Found in one study to be the best predictor of viral failure compared to self-report, facility pill counts, pharmacy refill and ARV drug monitoring methods^34^ and produced the most stringent (lowest) estimate of adherence (59%) compared to self-report (90%), pill count (74%) and blood ARV levels (605) ^50^  Using modelling and testing the model on a prospective cohort, electronic adherence was shown to predict short term gaps in retention (>72 hours without a dose) with ROC AUC: 0.85 [0.8-0.91] / Brier score 0.20, 73% classification accuracy, moderate specificity (87%) but low sensitivity (59%). A cut off of 89% average adherence optimised the classification accuracy to 78% [71-83%]^71^  Adjusted estimates - none of the modifications significantly altered the association between raw MEMS adherence and VL (sensitivity ranged from 71% for capped to 76% for diary cleaned or capped and cleaned) ^70^. Those whose classification changed through cleaning (diary/ capping/ both) were all virally suppressed. Without adjusting for capping or diary cleaning, the median MEMs adherence can reach over 100% (101.6%^70^). Capping the maximum daily adherence at 100% reduces the median adherence to below 100% (98.4%) and produces a more stringent (lower) estimate of adherence (65% vs 71% uncapped)^70^. | ^34^  ^52^  ^42^  ^50^  ^70^  ^71^  ^28^ |
| Adherence trajectory by MEMS | Adherence trajectory using multiple time points of MEMS data | Reflects specified interval | From the patient  Electronic pill count | **Categorisation:**  Adherent: ≥80% adherence by MEMS^72^  Non-adherent: <80% adherence by MEMS^72^  3 trajectories of adherence:  Consistently high  Steadily decreasing  Consistently low ^72^ |  |  | Baseline self-reported adherence (3-item self-report composite of ability and recall) was significantly associated with MEMS adherence trajectories^72^  Each day missing ≥1 dose increased odds of being in the low versus high adherence trajectory (OR: 1.64 [1.11-2.41]), while reporting “doing a very good job” (OR: 0.32 [0.13-0.79]) and “almost always” taking HIV medicines as prescribed (OR: 0.33 [0.13-0.86]) decreased odds^72^ | ^72^ |
| Healthcare-worker pill counts | Measure of adherence by counting unused pills and calculating pills taken from this | Reflects period since last refill: usually one to two months ^34,50^. Can reflect multiple refill intervals | From the patient  Tends to be collected by the pharmacist when the patient returns to the facility^73^ or by unannounced home visit^74^   - Remaining tablets counted ^75^ - Can use a fixed dose tablet or individual drugs in a regimen ^34^   From routine patient records   - Number pills dispensed   ^4,34,50,76^ | **Calculation:**  Adherence = (#pills dispensed - # pills returned)/([date returned- date dispensed][#doses per day])*100 ^4,34,50,73,74,77,78^  Decisions to be made:   1. Cap adherence at 100% for each interval 2. Allow over-adherence^79^   **Categorisation:**  Dichotomised  Adherent: ≥95%^75–78^ or <80%^76^ pills taken  Non-adherent: <95% pills taken (i.e. corresponding to 29 out of 30 days intake for a single pill daily prescription) ^75–78^ or <80%^76^ pills taken  OR  Sub-categorised as  Good adherence: ≥95% pills taken  Fair adherence: 90-94.9%  Poor adherence: <90% ^50,51^  OR  High average trajectory of adherence  High but slowly decreasing trajectory of adherence  Decreasing then rapidly increasing trajectory of adherence | Quantifiable^28^ with tangible evidence of no- adherence in returned pills^52^  Feasible:   - Easy to use^28^ - Inexpensive^28^ - Widely accessible^34^   Pill counts both identify adherence and act to help improve adherence - twofold benefit ^79^ | Require patient cooperation to return pills - risk of forgetting and pill dumping^52^, moving pills to another container or pill hoarding^28^, which changes interpretation ^76^  Does not record actual consumption^28^  May overestimate adherence^52^ and not associated as strongly with viral outcomes compared to more objective measures ^68^  Too time consuming^28^ in a busy clinic ^4^  Needs a long follow up period^73^ and requires a separate recording process in the pharmacy that is often not part of routine dispensing operations ^28^  Low to moderate specificity and moderate sensitivity | Unannounced pill counts conducted in patients’ homes have demonstrated validity for monitoring medication adherence ^80,81^  Associated with viral failure: AUC 0.64 [0.52 to 0.76] and adjOR 0.88 [0.80-0.96] ^34^, and with sustained VL suppression (<400 copies/mL x 3): adj RR: <90% adherence 0.65 [0.48-0.90] and 90-94.9% adherence 0.71 [0.56-0.90] compared to >95% adherence^50^  Varied by threshold and trajectory   - <95% adherence by pill count predicted viral failure with moderate sensitivity 70.4% and specificity: 77.3%^76^ - Prediction of viraemia was best with a threshold of 90%: ROC AUC: 0.53 with and without adjustment for CD4. Sensitivity 82% (Specificity 25%) ^4^ - Threshold of <80% underestimated viral non suppression and threshold <95% overestimated it ^76^ - Probability of VL<1000 increased when shifting status from decreasing/ increasing group to high slowly decreasing: OR 2.88 [1.52-5.45]or to constantly high: OR 2.91 [1.74-4.86] ^73^   Associated with genotypic ARV resistance:  adjOR 0.82 (0.69-0.98) ^34^. Association with CD4 is variable: the higher the average adherence, the higher the rate of change of CD4/ improved immunological recovery with variations by trajectory^73^ but there was no association with CD4 trend in another study^50^  Higher instantaneous risk of death associated with lower average adherence: adj HR: 0.73 [0.66-0.81] ^73^  Only pill count was associated with sustained VL suppression (<400 copies/mL x 3) compared to self-report, electronic monitoring and blood ARV levels^50^ and had a higher sensitivity and specificity than self-report (VAS and Likert scale) ^76^. Produced a lower (more stringent) estimate of adherence than the CASE Index (38% vs 61%) ^78^, than self-report of doses missed since last visit (74% vs 90%), and a lower estimate (25%) than VAS (87%), appointment keeping (79%), SHCS-AQ (72%) or pharmacy refill (54%) ^4^ and lower estimates in home-based evaluations of adherence (59%) than self-reports (86%) ^75^, but higher estimates than electronic monitoring (59%) and blood ARV levels (60%) ^50^ | ^34^  ^52^  ^77^  ^78^  ^51^  ^50^  ^79^  ^4^  ^76^  ^73^  ^74^  ^75^  ^28^ |
| Pill count variance | A high average adherence is usually linked to a low variance of adherence | Reflects period since last refill: usually one to two months ^34,50^. Can reflect multiple refill intervals | From the patient  Pill count adherence | **Calculation:**  A measure of variance that is independent of the average was designed and provided standardised variances of adherence^73^  - The averages of adherence were first sorted by increasing order then grouped into small-amplitude classes of nearly 30 averages.  - The corresponding variances were sorted in increasing order within each class and assigned a rank.  - The empirical repartition function of the variances within each class of averages was obtained by dividing the ranks of the ordered variances by 1 + the class size.  - The quantiles of this empirical repartition function were then assimilated to the quantiles of a normal distribution^73^ | Dissociating average and variance of adherence allows evaluation of effects of variance at difference adherence levels ^73^  A transformation of the variance provides a more efficient measurement of the variability in adherence that is independent of the average adherence and allows focusing on the impacts of the variance that are not repeats of the impacts of the mean^73^ | Needs a long follow up period ^73^  Interpretation of the effect size is difficult because of the transformed measure ^73^ | Found 3 adherence variance trajectories:  High/ Moderate/ Low variance ^73^  Increase in viral suppression with lower variance within each adherence trajectory: suppression increased when shifting status from high variance to moderate variance OR 1.76 [1.35-2.3] or low variance: OR 1.82 [1.25-2.66]^73^  Decrease in CD4 with increased variance, but similar CD4 change between high and moderate variance. Moderate variance actually decreased the monthly CD4 change, compared to low variance which increased the monthly CD4 change ^73^  Higher instantaneous risk of death associated with higher adherence variance: adj HR 1.45 [1.03-2.06]. The higher the average adherence the greater the effect of the variance on death ^73^ | ^73^ |
| Over-adherence by pill count | Identifies pill dumping as a measure of non-adherence | Reflects period since last refill: usually one to two months ^34,50^. Can reflect multiple refill intervals | From the patient  Pill count adherence^79^ | **Calculation:**  Adherence calculated at every medication refill = (pills dispensed-pills returned)/)pills prescribed per day x days since last refill)x100^79^  Calculate the number of intervals for which adherence is >100%^79^  **Categorisation:**  Adherent: <33% of refills have over-adherence  Over-adherence if adherence is >100% for >33% of their refills^79^ | Useful in settings where more objective measurements are not available ^79^ | Validated in adolescents who are treatment experienced and used to receiving feedback on their pill counts ^79^  High misclassification (false negatives and low sensitivity)  Does not identify those who are good at deception and remove the correct number of pills - cannot discriminate from those who have consistently good adherence ^79^  HCW response to using this metric may create the same issue as for pill counts - accustom patients to a response depending on their pill count and 'train' them to pill dump more accurately ^79^ | Sensitivity to detect patients with viraemia (VL > 400 copies/mL) was poor (33% [20-48]) but specificity was high (87% [82-91]), with an adj OR 3.3 [1.5-4.2] for over-adherent vs adherent patients ^79^  A detectable viral load was more common in those with apparent over-adherence (> one third of pill counts with >100% adherence) by pill count (33%) than in those with no over-adherence (13%). p=0.001 ^79^  Median MEMS adherence was lower in the group with over-adherence (87.2% [48.2-98.7]) than those without over-adherence (95.4% [86.6-98.8]) ^79^ | ^79^ |
| Self-report | | | | | | | | |
| Quantification of pill-taking through recall | | | | | | | | |
| Visual analogue scale (VAS) | A self-reported adherence tool that allows a patient to mark a point on a visual continuum that reflects their pill taking in a specific time period | Reflects a specified interval   - Generally used to reflect the previous month ^4,32,36^   But also used for shorter periods, such as previous 3 days ^43^ | From patient through interview  Question:   1. "How much of your medication have you taken in the last <period>?"^45^   Patient marks a point on a visual continuum line from 0 to 2^43^, 0 to 10^32^ or 0 to 100, reflecting the proportion of ART that was taken in the previous month  Additional option of pictogram enhancement: thumbs up, sidewards and down corresponding to 100%, 50% and 0%^45^ | **Presentation:**  A percentage corresponding to the proportion of adherence from the continuum ^4,36,76,82^  **Categorisation:**  Dichotomised  Adherent: missed no doses^43^ or proportion of pills taken 100% ^36^, ≥95%^76^, ≥90%^45,83^ or ≥80%^76^  Non-adherent: missed ≥1 dose^43^ or proportion of pills taken or 100% ^36^, <95%, <90%^45,83^ or <80%^76^  OR  High adherence = ≥90%  Moderate adherence =  80-90%  Low adherence  <80%^32,63^  **Interpretation:**  Higher score means better adherence^36^ | Good feasibility^5^   - Rapid to administer and assess: important in resource constrained settings with busy health providers^5,45^ - Low burden of response for the patient^5^ - Low burden for the provider: does not require calculation^5^ - Inexpensive^45^   Less dependent on HCW communication skills than the standard questionnaire^45^  VAS is most strongly associated with CAS and could form a simple tool at the point of care to replace the composite measure^32^  Easier to apply in illiterate patients than other questionnaires, particularly with pictogram enhancement^45^  More sensitive to detection of adherence problems than the standardised questionnaire^45^ | Subjective and susceptible to bias^5^ and the tendency to overestimate adherence  Accuracy undermined by recall and social desirability bias, even prefaced by normalising language ^4,5,32^ and is influenced by the provider’s attitude^5^  Likert VAS requires some abstract thinking and reasoning, and still requires some literacy, even with pictogram enhancement^45^  Self-reports depend on the communication ability of the health provider - the self-reported detection reduced over time (waning effect of the intervention impact) whereas the drug concentration and VL measures were more steady^45^ | Validated by Giordano et al in 2004^84^ and the pictogram enhancements were validated by Glass et al in 2008 ^85^  Odds of viral failure (>1000 copies/mL) were 124.0 (14.1-1090.4) times greater for low and 12.4 (1.1-143.0) times greater for moderate compared to high adherence^32^ with 60%^76^ to 88%^83^ specificity but low sensitivity (25%^83^ to 52%^76^), but other studies found a non-significant association with virologic failure: OR 0.86 [0.57-1.30]^36^ and non-significant association (80-94% = adj RR0.28 [0.05-1.7] and <80% = adj RR 0.61 [0.05-6.8] compared to ≥95%)^63^  The pictogram-enhanced VAS had low agreement with viral failure: VAS at visit 2 had a kappa of 0.216 for the agreement between VAS≤7 and a kappa of 0,171 for the agreement of VAS≤8 and viral failure and produced a higher estimate of non-adherence (identified more patients with adherence problems) than VL did (defined as viral failure: VL>1000 copies/mL) (17.2 vs 9.1% at visit 1 and 11.7 vs 9.2% at visit 2) ^45^  Best threshold for predicting viraemia (>400 copies/mL) was 95%: ROC AUC: 0.53, 0.56 with CD4 ^4^. Threshold of <80% underestimated viral non suppression and threshold <95% overestimated it ^76^  Not associated with CD4 count outcomes ^5^  Correlated with four-day recall (r = 0.58 / kappa = 0.32 ^5^ and r=0.49^32^), pharmacy refill (r=0.66)^32^ and CAS (r=0.83)^32^  Produces lower estimates of adherence than four-day recall and retention (68% vs 83% and 73% respectively) ^5^ but higher estimates of adherence than AACTG self-report or plasma ARV concentration (89% vs 80% and 74% respectively) ^43^ and higher estimates of adherence (87%) than appointment keeping (79%), SHCS-AQ (72%), pharmacy refill (54%) and pill count (255) ^4^. The pictogram-enhanced VAS had strong agreement (kappa range 0.285-0.742) but produced a higher estimate of non-adherence than the two-item 4 week missed dose self-reported measure (17.2 vs 10.7% at visit 1 and 11.7 vs 5.7% at visit 2) ^45^  Poor agreement with adherence measured by plasma ARV concentration: Kappa 0.101 and ROC AUC: 0.543^43^, weakly associated with atazanavir hair concentrations^63^ and not associated with tenofovir hair concentrations ^61^ | ^5^  ^86^  ^43^  ^32^  ^36^  ^4^  ^61^  ^63^  ^82^  ^83^  ^76^  ^45^ |
| Self-reported ART interruption | Patient report of any episode of ART interruption (≥48 consecutive hours of missed ART)^36,40^ | Reflects the pattern of adherence^36,40^  over a specified time period, such as the previous 3 months^36,40^ | From the patient through interview  1. In the past 3 months, did you have a day when you did not take any ARV drugs,  🡪If yes: what were the most days in a row that you missed swallowing your drugs in the past 3 months?^36,40^ | **Calculation:**  Number of interruptions in time period  **Categorisation:**  Dichotomised  Good adherence: No treatment interruptions (100% adherence) ^36^  Poor adherence: ≥1 treatment interruption of ≥48 hours^40^ (non- perfect adherence^36^) |  | Susceptible to social desirability and recall biases^40^  Low sensitivity for screening | Strong and significant association with VL failure (VL ≥1000 copies/mL): OR 2.86 [1.56-5.26] but able to identify only 11% of virological failures^36^. Predicted viral failure with high specificity 88%[85-91%] but low sensitivity 36%[20-55%]^87^  Had a stronger association with viral failure and produced a lower estimate of adherence than other measures including pharmacy-based medication possession ratio <90%, healthcare provider estimates of adherence and self-report (VAS, AACTG, CASE Index, 30 day recall) ^36^  Not significantly associated with self-management of HIV^40^ | ^36^  ^40^  ^87^ |
| Weekend recall | Self-reported weekend-specific non-adherence^88^ | Reflects the previous weekend^88^ | From the patient through interview  Single question  “Did you forget to take any of your HIV medications over the past weekend?” ^88^ | **Categorisation:**  Adherent: not missing a dose  Non adherent: missing 1/2 doses^88^ | Event-level measurement describes a specific risk behaviour during a recent time window rather than reporting overall rates of behaviour, to minimize overreporting, recall, and social desirability biases^88^ | Self-report is still susceptible to social desirability and recall bias even with the use of a short term measure to mitigate this^88^  Weekend nonadherence may not be representative of the patient's cumulative behaviour^88^ | Item drawn from a 6 item self-report measure from Kagee and Nel validated in 2012: the weekend item correlated with adherence in the past week (r=0.31), in the past 2 weeks (0.37) and the past month (0.31) ^89^  Non-adherence was weakly associated with a detectable viral load (chi squared 2.05, p=0.15)^88^ | ^88^ |
| Two-day recall | Self-report of adherence over the previous 2 days | Reflects the 2 days prior to the consultation | From patient through interview  Number of doses taken in previous two days | **Categorisation:**  Adherent: not missing a dose  Non adherent: missing 1/2 doses |  |  |  | ^82^ |
| Three-day recall | Self-report of adherence over the previous 3 days | Reflects the 3 days prior to the consultation | From patient through interview   1. “Did you swallow your pills  - Yesterday - 2 days ago   3 days ago?” | **Presentation:**  Proportion of pills taken  **Calculation:**  Adherence = # pills taken/3*100 | Widely accessible^34^ | Overestimates adherence^34^ | Measure did not predict viral failure (by OR or AUC) or genotypic ARV resistance^34^.  Produced a higher estimate of adherence than other more objective measures: overestimated adherence^34^ | ^34^ |
| Four-day recall | Self-report of adherence to medication over the previous 4 days | Quantitative measure of adherence ^77^ over the 4 days prior to the consultation^31,77^ | From patient through interview   1. Self-report the number of times pills were taken^5^ or forgotten^31^ in the previous four days | **Presentation:**  Proportion of pills taken  **Calculation:**  Percentage adherence^77^:  Adherence = number of pills taken/ number of pills prescribed*100  **Categorisation:**  Dichotomised  Optimal adherence: 100% ^31^ or ≥95% ^5,77^ of pills taken  Sub-optimal adherence: <100% ^31^ or <95% ^5,77^ of pills taken | Recall for events remembered is more accurate than events that were missed^5^ | Subjective and susceptible to the tendency to overestimate adherence^5,31,86^  Susceptible to recall bias, social desirability bias and influence by the provider’s attitude^5^  Requires a base level of cognition^5^  Easier measure for once daily than twice-daily regimens^5^ | Not associated with CD4 count outcomes ^5^  Correlated with visual analogue scale (r = 0.58 / kappa = 0.32)^5^  In one study 4-day recall had the lowest estimate of adherence (7.2%) compared to pill count and qualitative adherence, or a composite of the two^77^  Self-report collected by a psychologist was associated with schedule adherence: proportion of observed agreement: 76.4%/ Cohen's Kappa = 0.317 (95% CI 0.138-0.495)^31^ | ^5^  ^86^  ^77^  ^31^ |
| One week recall | Self-report of adherence to medication over the previous 7 days | Reflects adherence over the previous 7 days ^90^ | From the patient through interview   1. Number of ART doses taken^20,90^, missed^75^ or been >2 hours late in a dose^7^ in the previous week | **Calculation:**  Adherence = number missed doses/ 7(14 if twice daily dosing)^7^  **Categorisation:**  Dichotomised  Adherent: 100%^90^, ≥90%^20^ or ≥80%^20^ of pills taken  Non-adherent: <100%^90^, <90%^205,77^ or <80%^20^ of pills taken  OR  Adherent: none missed^7^  Moderate adherence: 1 missed (90%)^7^  Non-adherent: ≥2 missed^7^,  **Interpretation:**  Higher score is better adherence ^90^ |  | Ceiling effect seen – many patients with perfect scores ^90^ | Moderate (1 missed): HR 1.89 [1.09-3.29] and non-adherence: (≥2 missed): HR 2.48 [1.20-5.14]) associated with viraemia (>100 copies/mL) compared to 100% adherence ^7^  <90% adherence by one-week recall was associated with viral non suppression (>400 copies/mL): OR 1.14 (1.02-1.28) ^20^  Adherence (85.6%) was 27% higher than pill count adherence (58.6%) ^75^ and more weakly associated with viral outcomes than pharmacy measures^20^ | ^7^  ^20^  ^90^  ^75^ |
| Two week recall | Self-report of adherence to medication over the previous 14 days | Reflects adherence over the previous 2 weeks ^91^ | From the patient through interview   1. Number of doses taken in previous two weeks   Number of doses prescribed for previous two weeks ^91^ | **Presentation:**  Percentage of doses taken relative to the number of doses required over the previous two weeks ^91^  **Calculation:**  Adherence = required number of doses per day X 14 - Number of  doses missed X 100%  /  Required number of doses per day X 14 ^91^  **Categorisation:**  Adherent: 100% or ≥95% adherent  Non-adherent: <100% or < 95% adherent^91^ |  | Evaluation of adherence in those attending their appointment is skewed as those with good retention are more likely to be adherent^91^ |  | ^91^ |
| Combined one to two week self-report | Measures a binary outcome and quantification of recall adherence | Reflects a week to two weeks | From the patient through interview   1. Dichotomous:. ‘Have you missed any doses of your medication in the last two weeks, yes or no?’ 2. Categorical: ‘Think about the past week (7 days); on average, how often did you miss a dose of medication?’  - Response options included, ‘(1) once a day; (2) more than once a week, but not every day; (3) once a week; or (4) I do not miss my medicine.’ ^59^ | **Categorisation:**  Adequate adherence: no missed doses  Inadequate adherence: reporting any missed doses on either question (non-perfect score) ^59^ |  | Miss-classify patients who are adherent by VL^59^ | Not associated with viraemia >400 copies/mL ^59^ | ^59^ |
| Recall since previous visit | Self-report of adherence to medication since the previous visit (usually 1-2 months) | Reflects previous 30 days ^36^ or 4 weeks ^7^ or longer since previous visit ^50^ | From the patient through interview   1. Number of ART tablets missed^36,50^ or been >2 hours late^7^ | **Calculation:**  Adherence = (#missed tablets)/(total # ART pills over period) ^36^  **Categorisation:**  Dichotomised  Adherent: 100%^36^ of pills taken  Non-adherent: <100%^36^ of pills taken  OR  Adherent: none missed^7^ or ≥95%^50,51^ of pills taken  Moderate adherence: 1 missed (90%)^7^, 90-94%^50^ or 85-94% pills taken^51^  Non-adherent: ≥2 missed^7^, <90%^50^ or <85%^51^ of pills taken |  |  | Single item one month recall validated by Feldman et al in 2013 ^92^  Non-significant association with viral failure (VL ≥1000 copies/mL): OR 1.04 [0.63-1.69]^36^ and <90% adherence with sustained viraemia (3 episodes VL >400 copies/mL): adj RR 0.56 [0.30-1.04] ^50^, but associated with viraemia (>100 copies/mL) compared to 100% adherence (moderate (1 missed): HR: 2.55 [1.50-4.35] and non-adherent (≥2 missed): HR 2.64 [1.31-5.32]) ^7^  Not associated with CD4 trend ^50^  More strongly associated with viraemia than 1 week self-report, and repeated missed pills was more strongly associated than only missing one pill ^7^  16% higher estimate of adherence than pill count in another study (90% vs 74%) ^50^ | ^36^  ^51^  ^7^  ^50^ |
| Pill recall and interruption | Measures adherence interruptions and 4 week pill-taking recall | Reflects previous four weeks^45^ | From the patient through interview  2 questions  1) “How often have you missed a dose of your HIV medication in the past 4 weeks: daily, more than once a week, once a week, once every second week, once a month, never?" and  2) “Did you miss ART ≥ 2 days in a row in the last 4 weeks: yes or no?”. ^45^ | **Categorisation:**  Adherence = no missed doses  Non-adherence = ≥1 missed dose^45^ |  | Self-reports depend on the communication ability of the health provider - the self-reported detection reduced over time (waning effect of the intervention impact) whereas the drug concentration and VL measures were more steady^45^ | The questionnaire was not associated with viral failure but was strongly associated (though produced a lower estimate of adherence: 17.2 vs 10.7% at visit 1 and 11.7 vs 5.7% at visit 2) with a pictogram-enhanced VAS (kappa range 0.285-0.742) ^45^ | ^45^ |
| Swiss HIV Cohort Study Adherence Questionnaire (SHCS-AQ) | Measure of missed doses and treatment interruptions | Reflects adherence over the previous month ^4^ | From the patient through interview  Questions:   1. Frequency of missed doses 2. Episodes of missing 2 consecutive doses ^4^ | **Categorisation:**  Dichotomised  Adherent: missed no doses  Non-adherent = missed ≥1 dose/ missed 2 consecutive doses once^4^ |  | Accuracy undermined by social desirability bias ^4^ | Poor association with viraemia (VL>400 copies/mL) without adjustment for CD4: ROC AUC: 0.52 with low sensitivity 38% and specificity 66% ^4^ | ^4^ |
| Adherence trajectory by self-report | Adherence trajectory based on self-reported measures of adherence at two time points^93^ | Reflects the previous year^93^ | From the patient by interview  Self-reported pill recall at 6 and 12 months^93^ | **Categorisation:**  High adherence: ≥90% adherence^93^  Low adherence: <90% adherence^93^  Two adherence trajectories:  High adherence: a trajectory ending in high adherence at 12 months^93^  Low adherence: trajectory ending in low adherence at 12 months^93^ |  |  |  | ^93^ |
| Self-reported timing | | | | | | | | |
| Schedule adherence | Self-reported adherence to the schedule and timing of ART^77^ within 2 hours of recommended dosing^1^ | Reflects the previous 4 days | From the patient through interview  Question:   1. Self-reported timing of ART^77^ or number of days over 4 days patient was not adherent to the ART schedule^31^ | **Categorisation:**  Non-adherence: ≥1 gap (>2 hour) between scheduled and actual time of taking ART^77^ |  | Susceptible to overestimation^31^ | Psychologist-collected self-report associated with self-reported pill recall: proportion of observed agreement: 76.4%/ Cohen's Kappa 0.317 [0.138-0.495] ^31^ | ^31^  ^77^ |
| ART adherence while drinking pattern questionnaire | Patterns of ART use when participating in a specific activity e.g. drinking alcohol ^94^ | Reflects general timing of ART ingestion relative to activity ^94^ | From the patient through interview  3 questions on a Likert scale ranging from “rarely (1)” to “always (4)”.:   1. Early ART taking was based on the item: “How often do you take your ART early if you know that you will be drinking?” 2. Simultaneous ART taking was based on the item: “How often do you take your ART even if you have been drinking or are drunk?” 3. Skipping ART taking was based on the summed score of two items: “How often do you skip doses of your ART if you have been drinking?” and “How often do you not take your ART if you have been drinking?”^94^ | **Categorisation:**  1. Earlier ART dosing (relative to drinking)  2. ART use concurrent (with alcohol)  3. Skipping ART (when drinking) ^94^ | Help understand patient decision making - categorise 'non-adherence' into decisions that were made in order to be adherent or not  e.g. taking ART earlier pattern indicates non adherence but with an attempt to problem solve and still take ART ^94^ | Self-report susceptible to social desirability bias^94^ | Formative qualitative research by the authors (unpublished) identified the three patterns, which were converted into Likert scales to quantify the frequency each pattern took place ^94^ | ^94^ |
| Composite self-reports | | | | | | | | |
| Center for Adherence Support Evaluation (CASE) Index Score | Composite measure of ability to adhere and pill-taking recall | Reflects previous 3 months | From the patient through interview  3 simple questions   1. How often you have difficulty taking medication on time (four point Likert scale from 1[all of the time] to 4[never]) 2. On average how many days per week do you miss at least one dose (six point Likert scale from 1[everyday] to 6[never]) 3. When was the last time you missed one dose? (six point Likert scale from 1[within the past week] to 6[never])^36,95^ | **Calculation:**  Sum the scores for each component  Range 3-16^95^ - converted to a percentage^36^  **Categorisation:**  Dichotomised  Optimal or good adherence: score ≥10^36,78,95,96^  Poor adherence: score <10 ^36,78,95,96^  Or  Perfect adherence = 100%^36^  Non perfect adherence: <100%^36^  **Interpretation:**  Higher scores indicate better adherence  with 0 = complete non adherence and 100 = perfect adherence^36,95^ | Simple, practical ^95^ | Low sensitivity for viral failure ^95^ | Validated by Mannheimer et al in 2006^97^  Mixed evidence of association with viral outcomes, from non-significant association with viral failure (VL ≥1000 copies/mL): OR 1.30 [0.84-2.01]^36^ but a score <10 was associated with a raised VL (viraemia and failure: adjOR 13 [1.1-147.9])^95^. The index performed better in screening for viraemia >50 copies/mL (moderate sensitivity 80% and low specificity 20%) than for viral failure (low sensitivity 40% and specificity 18%)^95^  CASE Index produced a higher estimate of adherence than facility-based pill count (61% vs 38%) ^78^  Associated with HIV_ASES: women with low HIV treatment adherence self-efficacy were 10 times less likely to adhere to ART than women with high HIV treatment adherence self-efficacy: OR 0.1 [0.05 - 0.41]^96^ | ^95^  ^36^  ^78^  ^96^ |
| Composite of recall and ability | Composite measure of ability to adhere and pill-taking recall | Reflects the 4 days prior to the consultation | From patient through interview  Four items with yes/no response:   1. Do you sometimes find it difficult to remember to take your medicine? 2. When you feel better, do you sometimes stop taking your medicine? 3. Thinking back over the past four days, have you missed any of your doses? 4. Sometimes if you feel worse when you take the medicine, do you stop talking it?^32,86^   ± Barriers and enhancers:   - What causes you to miss some doses of your drug?”,   “What helps you remember to take your drugs?”^32^ | **Scoring:**  1 for a no, 0 for a yes  **Calculation:**  Sum total score  **Categorisation:**  Highly adherence = no to all 4 items  Moderately adherent = yes to 1 item  Poorly adherent = yes to ≥2 items^86^ | Questions were formulated so that 'no' was a correct answer to reduce 'white coat effect' ^32^ | Self-report is prone to recall and social desirability bias, even prefaced by normalising language ^32^ | Developed by Steel et al in 2007^98^  Odds of viral failure (>1000 copies/mL) were 21.2 (4.0-111.2) times greater for low and 1.4 (0.3-6.9) times greater for moderate compared to high adherence^32^  Produced higher estimates of retention than the visual analogue scale in some studies (83% vs 68%) ^5^ and lower than the visual analogue scale in other studies (11% vs 87%) ^86^  Correlated with Pharmacy refill (r=0.36), VAS (r=0.49) and CAS (r=0.71) ^32^ | ^86^  ^32^ |
| 3 item self-report composite of ability and recall | Composite measure of ability to adhere and pill-taking recall | Reflects adherence over previous month^99,100^ to 6 months^74^ | From the patient through interview  3 questions  1. Quantification of missed doses: number of missed doses in the preceding 30 days^99,100^ /4-weeks and 6-months ^74^  2. Rating of adherence quality/ability: “how good a job did you do taking your medications in the way you were supposed to?” ^99,100^  Six-point Likert scale from 1 (very poor) - 6 (excellent)^99,100^  Or “Are you able to take all of their medications as prescribed” ^74^   1. Frequency of poor adherence: “how often did you take your medications in the way that you were supposed to?” ^74,99,100^   6-point Likert scale from 1 (never) to 6 (always)^99,100^ | **Calculation:**  Reported as score for each question and the combined score  1. Missed medication = number not missed/ monthx100  = (30-missed doses)/30*100  2. score out of 6/6*100  3. score out of 6/6*100^99^  Scores summed with equal weight to get a percentage from 0 to 100^99,100^  Calculate change in score (individual item and combined score) between subsequent visits ^99^  **Interpretation:**  The higher the score the better the adherence ^99,100^  **Categorisation:**  Thresholds set at smallest increments of a decrease/increase of 1 - 1 missed dose/ 1 level on the Likert scale ^100^  Dichotomise combined score percentage  Good adherence: 100% or ≥80%  Poor adherence: <100% or <80%  Thresholds previously shown to have a good balance between sensitivity and specificity^100^ | The high sensitivity and NPV of the 100%/<100% adherence threshold for both viraemia and viral failure make it a good first stage screening test for poor adherence ^100^  Longitudinal measure if repeated that allows evaluation of relative changes in adherence to help account for individual reporting patterns^99^  Feasible in routine care in low resource settings ^99^  Do not need to convert scores - plot score to each answer at each visit and if there is a drop in one/ combination are flag for further evaluation^99^  Simple and easily understood questions that were developed to minimise social desirability bias and be easily understood ^100^  Did not show a ceiling effect so may be more sensitive to reported non-adherence than other scales that did show ceiling effects (but not directly compared). The scale may be detecting more subtle difficulties that have not yet impacted on VL outcomes^100^ | May overestimate adherence on a population level as it censored those who were lost to follow up - but representative of real life where you assume these are non-adherence ^99^  Longitudinal relationships between changes in self-reported adherence and VL likely to vary with context, including the distribution of reported VL adherence and VL levels in the population ^99^ | Developed and validated by Wilson et al with a Cronbach’s alpha of 0.86 in the USA ^101,102^  Cross-sectional validity of the translated scale items and overall scale score were evaluated in a South African PMTCT setting with a Cronbach alpha of 0.79^100^  The scale had moderate ability to discriminate between patients with elevated and non-elevated VL^100^, with a significant but small association with viraemia (≥50 copies/mL): ROC AUC 0.599 and failure (≥1000 copies/mL): ROC AUC 0.656 that did not vary much across subgroups (SES and psychosocial characteristics) and had high sensitivity and NPV (and similar specificity) detecting viraemia, as well as a very high VL (≥10 000 copies/mL): ROC AUC 0.642^100^  Increasing the threshold for adherence (from 80 to 90 to 100%): increased sensitivity and NPV, but decreased specificity. Threshold of 100% had high sensitivity (94% for VL>50 and 97% for VL>1000 copies/mL) and NPV (91% for VL>50 and 98% for VL>1000 copies/mL) for both viraemia and viral failure^100^  Did not perform as well as blood ARV levels in predicting VL suppression <50 copies/mL^44^  The scale can be used longitudinally if repeat measures are conducted. The best-performing thresholds to predict VL .50 copies per mLa were a single-level decrease on the Likert response item “how good a job did you do at taking your HIV medicines in the way that you were supposed to?” (OR 2.08, 95% CI: 1.48 to 2.91), and a decrease equivalent to ≥5 missed doses or a one-level decrease in score on either of 2 Likert items (OR 1.34, 95% CI: 1.06 to 1.69)^99^ | ^74^  ^100^  ^99^  ^41^ |
| South African National Department of Health Adherence Questionnaire | Composite measure of ability to adhere, pill-taking recall and pill identification | Reflects the previous four days | From the patient through interview   1. Do you sometimes find it difficult to remember to take your medication? 2. When you feel better, do you sometimes stop taking your medication? 3. Sometimes if you feel worse after taking your medication so you stop taking it? 4. Thinking back over the past 4 days, have you missed any of your doses? 5. VAS over previous 4 days: mark adherence from 0 (not good at all) to 10 (very good)   6. Pill identification   - Tick the drugs from the list you are taking - Write down the number of pills of each that you take each day   What time do you take this drug (in the morning and in the evening) ^48^ | **Categorisation:**  Poor adherence = any of:   - Yes to Q1,2,3,4 - <8 in Q5   Identify <2/3 tablets or identify correct number of pills or identify correct time to take pills |  | Low sensitivity and specificity to predict VL and ARV concentration | Low sensitivity (50% [28.9-71.1%]) and specificity (66.3% [60.3-71.9%]) of poor adherence to detect virological failure (VL ≥1000copies/mL) ^48^  Low sensitivity (43.9% [28.5-60.3%]) and specificity (68.2% [61.6-74.2%]) for predicting detectable efavirenz levels ^48^  Lower sensitivity but higher specificity than the SMAQ for virologic failure (80% and 43% respectively) and efavirenz levels (66% and 45% respectively)^48^ | ^48^ |
| Pattern of adherence by self-report | Measure of short-term pill recall adjusted by 7 day recall, schedule adherence and treatment interruptions^103^ | Reflects adherence over the previous 4 days and previous 4 weeks^103^ | From patient through interview  14-item validated scale assessing   1. Number pills of each ARV taken each day over previous 4 days 2. How well they adhered to the dosing schedule over the previous 4 days and 4 weeks 3. Occurrence of treatment interruptions (>2 consecutive days without pills taken) in previous 4 weeks 4. Number of pills taken each day over previous 7 days ^103^ | **Presentation:**  For one or two consecutive visits  **Calculation:**  Calculate percentage adherence and adjust using 7 day adherence percentage to reclassify patients who underreported adherence in the 4 day/ 4 week questionnaire^103^  **Categorisation:**  High adherence: 100% doses taken over 4 days and completely adherence to the prescription schedules over 4 weeks with no interruptions  (±at both visits)  Moderate adherence: 80% adherence (missed <20% doses) over 4 days and 4 weeks, or had mostly adhered to the schedule over 4 weeks with no interruptions  (±at either or both visits)  Low adherence: <80% adherence (missed >20% doses) over 4 days or did not follow the schedule over 4 weeks with no interruptions  (±at either or both visits)  Treatment interrupter: presence of ≥1 interruption of >2 days no treatment in the previous 4 weeks  (±at either or both visits)^103^ | Differentiated short term from long term adherence and mean adherence from treatment interruptions^103^ | Susceptible to reporting bias ^103^ | Emergence of early (at six months) or late (>six months) ARV drug resistance was associated with different patterns of adherence over time^103^   - Low early adherence (month one and three) was associated with early resistance: aOR 8.51 [1.30-55.61] - Early treatment interruptions were associated with early (at six months: aOR 5.25 [1.45-18.95]) and late (>six months: aOR 3.72 [1.27-10.92]) resistance - Moderate adherence (early/late), late (month six and 12) adherence (all categories) and interruptions was not predictive ^103^ | ^103^ |
| VAS-SMAQ-FDR | Composite score made up of pill recall (visual analogue scale and four-day recall) and the SMAQ (pill recall, knowledge of pills and assessment of specific behaviours) | Covers a range from the previous weekend to the previous 3 months | From patient through interview:   1. VAS 2. Four-day recall  - Do you sometimes find it difficult to remember to take your medicine? - When you feel better, do you sometimes stop taking your medicine? - Thinking back over the past four days, have you missed any of your doses? - Sometimes if you feel worse when you take the medicine, do you stop talking it?  1. SMAQ | **Categorisation:**  High adherence: highly adherent by all 3 components (no to all 4 items, >95% on VAS and adherent by SMAQ)  Low adherence: <4/4 for four-day recall OR ≤95% VAS adherence OR non adherent by SMAQ |  | Self-report susceptible to recall bias and the tendency to overestimate adherence | Produced a lower estimate of adherence (27%) than the VAS component (87%) or the SMAQ component (31%) but greater than the four-day recall component (11%) ^86^ | ^86^ |
| VAS-SHCS-AQ | Composite measure of adherence made up of the VAS pill recall and the SHCS-AQ (pill recall and interruptions) | Previous month ^4^ | From patient through interview:   1. VAS 2. SHCS-AQ ^4^ | **Categorisation:**  Adherent: missed no doses  Non adherent: missed ≥1 dose/ missed 2 consecutive doses once ^4^ |  |  | Validated by Deschamps et al in 2008 ^104^ | ^4^ |
| VAS-SMAQ-ID | Composite measure of adherence combining the VAS pill count, SMAQ (pill recall, knowledge of pills and assessment of specific behaviours)  and pill identification | Covers a range from the previous weekend to the previous 3 months | From patient through interview:   1. VAS 2. SMAQ 3. Pill identification ^83^ | **Categorisation:**  Adherent if meets adherence criteria of 3 component measures ^83^ |  |  | Composite adherence predicted viraemia (>400 copies/mL) with moderate specificity( 86.8%) but low sensitivity (18.4%)^83^   - This was lower sensitivity than VAS (25%) or SMAQ (27%) alone bur higher specificity than the SMAQ (79%) alone ^83^ | ^83^ |
| Godin’s self-report | Composite of pill-taking recall, pill identification, as well as barriers | Previous seven days ^38^ | From the patient through interview:  Questions:   1. List name of ARVs and times taken during the day 2. How many pills were missed over previous 2 days 3. During last 7 days did you    1. Do various leisure activities    2. Did one of the situations prevent you from taking ARVs 4. How many times during the last 7 days how many times did you miss your ARVs?^38^ | **Categorisation:**  Adherent: ≥95% average/ 7 day adherence  Non adherent: <95% average/7 day adherence ^38^ |  | Susceptible to be affected by social desirability bias and required extensive training of research assistants to avoid this^38^ | Validated by Godin et al in 2003 ^105^  Produced a higher estimate of adherence (90%) than pharmacy refills (82%) ^38^ | ^38^ |
| Adherence to Refills and Medication Scale (ARMS-7) | Composite self-reported measure that evaluates intentional and unintentional adherence, quantifies pill-taking and evaluated adherence behaviour | Reflects a general state | From the patient through interviews  ARMS7: 7 items scored on a 4 point scale.  COULD NOT FIND THE ARMS 7 ONLY THE 12 ITEM ORIGINAL SCALE. UNCLEAR WHICH OF THESE ITEMS WERE USED   1. How often do you forget to take your medicine? 2. How often do you decide not to take your medicine? 3. How often do you forget to get prescriptions filled? 4. How often do you run out of medicine? 5. How often do you skip a dose of your medicine before you go to the doctor? 6. How often do you miss taking you medicine when you feel better? 7. How often do you miss taking your medicine when you feel sick? 8. How often do you miss taking your medicine when you are careless? 9. How often do you change the dose of your medicines to suit your needs (like when you take more or less pill than you’re supposed to)? 10. How often do you forget to take your medicine when you are supposed to take it more than once a day? 11. How often do you put off refilling your medicines because they cost too much money?   How often do you plan ahead and refill your medicines before they run out? (reverse coded) | **Scoring:**  Minimum score = 7, max score = 28  **Interpretation:**  The higher the score the better the adherence |  | Self-report susceptible to social desirability and recall bias ^106^ | Validated self-report scale with a Cronbach alpha of 0.82 , developed by Kripalani et al for chronic disease in 2009 ^107,108^  Non adherence was associated with fair or poor health status report: adj OR 11.380 [1.527-84.785] ^106^  Found lower rates of adherence using this tool than other studies in the same population^106^ | ^106^ |
| Simplified Medication Questionnaire (SMAQ) | Composite of quantification of pill-taking, knowledge of pills and assessment of specific behaviours  such as intentional and unintentional adherence^109^ | Covers a range from the previous weekend to the previous 3 months | From patient through interview  **Unintentional** component of adherence: (wishes to adhere but is prevented) ^83,109^  Questions with yes/no answers   1. Do you ever forget to take your medicine?”^109^ 2. Are you careless at times about taking your medicine? ^109^   **Intentional** component of adherence: (deliberate non adherence) ^109^  Questions with yes/no answers   1. Sometimes if you feel worse, do you stop taking your medicines? ^48,109^   **Quantification of adherence/ recall**   1. Thinking about the last week, how often have you not taken your medicine?” ^48,83,109^ 2. Did you not take any of your medicine over the past weekend? ^48,109^ 3. Over the past three months, how many days have you not taken any medicine at all? ^48,109^:   more than 2 days, less than 2 days, can’t remember^48^ | **Categorisation:**  Dichotomised  Adherent: if no to questions 1-3 and 5 and > 2 doses missed for 4 and > 2 days missed for 6.  Non-adherent: if yes to questions 1-3 or 5 or > 2 doses missed for 4 or > 2 days missed for 6.  **Interpretation:**  Higher scores reflect worse adherence | Each item on its own may be insufficient to capture the construct but the combined metric is a valid measure of the underlying construct of adherence^109^  Short and easy to administer in a busy facility^86^  Can be repeated to evaluate adherence over time^86^  Consists of multi-time point recall adherence questions spanning the previous weekend to the previous three months, which may address the effect of ‘white-coat adherence’ ^48^ | Self-report susceptible to recall bias and social desirability bias^109^  Self-reports vulnerable to the tendency to overestimate adherence^86^  Doesn’t capture dynamic nature of adherence ^109^ | Scale developed and validated in Spain by Knobel et al in 2002 and based on the Morisky scale, with removal of the question about feeling better as many patients are asymptomatic and addition of questions to quantify pill-taking^110^  In a validation of the SMAQ in women in Ethiopia, the scale was found to be a valid measure of adherence to ART with:  - Adequate internal consistency reliability (the 6 items reflect the same construct of adherence to ART) with Cronbach’s alpha 0.72  - Moderate to excellent concurrent validity (each item is consistent over time) with Pearson’s product-moment correlation between two time points ranging from 0.49-0.80 for the individual items  - Factorial validity present suggesting construct validity (SMAQ measures adherence to ART). Factor loading were all positive (range 0.26-1.18) and statistically significant  - Strong factorial invariance (the SMAQ is interpreted the same across groups and performs equally in different groups) ^109^  Weak prediction of viraemia (VL>400) with low sensitivity (27%^83^ to 41%^69^) and specificity (63%^69^ to 79%^83^), and viral failure (sensitivity 80% and specificity 43%)^48^  SMAQ had better sensitivity for predicting virologic failure (80% vs vs 50%) and detectable efavirenz levels (66% vs 44%) compared to the South African NDoH Adherence questionnaire ^48^ | ^109^  ^86^  ^48^  ^69^  ^83^ |
| Adult AIDS Clinical Trials Group (AACTG) adherence questionnaire | Composite of pill-taking recall and schedule adherence | Previous 3^36^ or 4 days ^57^ | From the patient through interview   1. Number missed doses in past 4 days: none, ≥1, all doses^36,57,63^ (and number of doses prescribed^43^) 2. Followed the dosing schedule closely in past 4 days: Yes/No^63^ 3. Followed the instructions closely in past 4 days: Yes/No^63^ 4. Missed ≥1 dose at the previous weekend: Yes/No^63^ 5. Last time a dose was missed    - Within last 1 week, 1-2 weeks, 2-4 weeks, >1 month^57^    - 0-4 weeks ago, >4 weeks ago^63^ | **Calculation:**  Adherence = (#missed tablets)/(total # ART pills over period) *100 ^36,57^  Convert to reflect 7 days = 7 x 4 day report percentage   - - If no missed doses in 4 days - assume total of 4   - If last missed dose within the past week - assume 2 missed doses   - If last missed dose in last 1-2 weeks - assume 1 missed dose   - If last missed dose was 2-4 weeks ago - assume no missed doses if no missed dose in last month^57^   **Categorisation:**  Adherent: missed no doses^43^ or 100% adherence^36^  Non-adherent: missed ≥1 dose^43^ or <100%^36^ |  |  | Validated by Chesney et al in 2000^111^ and optimised by Reynolds et al in 2007 who suggested the ‘weekend’ item was redundant^112^  Non-significant association with viral failure (≥1000 copies/mL): OR 1.28 [0.68-2.42]^36^  Weakly associated with atazanavir hair concentrations^63^ but otherwise poor agreement with adherence measured by plasma ARV concentration: Kappa 0.081 and ROC AUC: 0.538 ^43^ and produced higher estimates of adherence than plasma ARV concentration (80% vs 74% respectively) ^43^ | ^57^  ^43^  ^36^  ^63^  ^24^ |
| AACTG derivative | Composite of the ability to take medication and pill-taking recall | Reflects multiple time periods: the previous week to 4 weeks | From the patient through interview  5 questions on Likert scales:  1. Rate their ability during the past 4 weeks to take all the ARV drugs prescribed (from ‘1 = very poor’ to ‘6 = excellent’)  2. How often during the past 4 weeks had they taken all ARV medications prescribed (from ‘1 = none of the time’ to ‘5 = all of the time’)  3. On how many days in the past week they had taken all anti-HIV medicines that were prescribed (from ‘1 = not 1 day’ to ‘5 = all 7 days’)  4. On how many days per week, on average, they had missed at least one dose of anti-HIV medication (from ‘1 = every day’ to ‘6 = never’)  5. When was the last time they had missed taking any of their ARV medications (from ‘1 = within the past week’ to ‘6 = never skip medications’) ^33^ | **Scoring:**  Each question score out of 5 or 6  Total score /28 ^33^  **Categorisation:**  Perfect adherence: full score for individual items or overall  Non-perfect adherence: < full score^33^    **Interpretation:**  Higher score reflects better adherence^33^ | High negative predictive value for detectable VL^33^  Easy to administer^33^  Facilitates bi-directional communication to encourage adherence problem solving^33^ | Low sensitivity for identifying a detectable VL^33^  Needs introductory statements to acknowledge difficulties taking medicine and training of interviewers to be non-judgemental to reduce social desirability bias in responses^33^ | Adapted from the AACTG^33^  Non-adherence associated with viral non-suppression ≥40 and ≥400 copies/mL, with a stronger association with VL ≥400 copies/mL (better predicts viral failure than viral blips)^33^  Less than perfect adherence associated with higher odds of a detectable VL ≥40 copies/mL (Specificity and OR increased with lower thresholds of adherence):  1.<Excellent: OR 1.47 [0.081-2.67]. Sensitivity 73%. NPV 93%  Significant difference <Good: OR 2.21 [1.02-4.78]. Sensitivity 15%. NPV 92%  2. < All of the time: OR 2.72 [1.43-5.15]. Sensitivity 25%. NPV 92%  3. < All 7 days: OR 3.99 [1.69-9.42]. Sensitivity 14%. NPV 92%  4. <Never missed ARVs: OR 2.68 [1.41-5.08]. Sensitivity 26%. NPV 92%  5. <Never missed ARVs: OR 3.09 [1.77-5.38]. Sensitivity 42%. NPV 93%^33^  Less than a perfect answer on question 2, 4 or 5 had >80% likelihood of distinguishing patients with detectable VL^33^ | ^33^ |
| Composite of different adherence measures | | | | | | | | |
| Global adherence | Composite score combining self-report and pill count | Covers a defined period | From patient  Pill count ^77^  Pharmacy refill dates and number pills prescribed  Through interview:   1. Quantitative four-day recall self-report^77^ 2. Schedule adherence: self-report of timing of ART^77^   Or   1. CASE Index^78^ | **Calculation**:  Percentage adherence for each item individually  **Categorisation**:  Adherent: adherent by self-report (CASE index ≥10^78^ OR pill recall ≥95%^77^ AND schedule adherence <2 hour gap^77,78^) AND pill count ≥95%^78^  Non-adherent: if non-adherent by self-report (CASE index <10^78^ OR pill recall <95%^77^ AND/OR schedule adherence >2 hour gap^77^) AND/OR pill count^77,78^ |  | Self-reported survey liable to recall and social desirability bias ^113^ | Using four-day recall and schedule adherence for the self-reported component, global adherence had the highest estimate of non-adherence compared to its component measures (38% vs four-day recall:7%, schedule adherence: 32% and pill count: 20%) ^77^  Using the CASE index for the self-reported component estimated a much lower rate of retention than self-report or pill count alone (25% vs 61% and 38% respectively) ^78^ | ^77^  ^78^  ^113^ |
| Composite adherence score (CAS) | Composite score comprising of six month pharmacy visit compliance, one month VAS and four-day pill recall and ability^32^ | Spans four days to six months^32^ | From patient records  Record review to note total number of refills in previous 6 months  From the patient through interview  Visual analogue scale: zero (no adherence) - 10 (optimal adherence) over previous 30 days^32^  Four day recall (Composite of recall and ability)   1. “Do you sometimes find it difficult to remember to take your medicine?”; 2. “When you feel better, do you sometimes stop taking your medicine?”; 3. “Thinking back over the past 4 days, have you missed any of your doses?”; and 4. “Sometimes if you feel worse when you take the medicine, do you stop taking it?” 5. Barriers and enhancers:   “What causes you to miss some doses of your drug?”, and  “What helps you remember to take your drugs?” | **Scoring:**  Adherence scored 1-3 for each measure (3 = high, 2= moderate, 1 = low) and summed to get the CAS, which was then transformed into a score /3^32^  **Categorisation:**  High adherence: score 3  ~ had 6 refills/no to all 4 questions/ ≥90% on VAS  Moderate adherence: score 2  ~missed 1 refill/ yes to 1 question/ 80-90%  Low adherence: score 1  ~ missed ≥2 refills/ yes to ≥2 questions/ <80%  If pharmacy score was low, CAS was scored as low (1) regardless of VAS and interview scores^32^ | Composite measure aims to overcome the issues with single measures^32^  Questions were formulated so that 'no' was a correct answer to reduce 'white coat effect' ^32^  Overweighting was reduced by scoring the CAS as 'low' regardless of VAS and interview scores if pharmacy score was low^32^  Normalising language was used to reduce social desirability bias^32^ | Use of a CAS may be cumbersome in routine clinical settings ^32^  Still vulnerable to limitations of component measures ^32^ | Odds of viral failure (VL >1000 copies/mL) were greater with low adherence compared to moderate adherence: OR 7.6 [1.8-30.8] ^32^ and viral suppression was found in 100% of those with high CAS adherence, 89.5% of those with moderate CAS adherence and 52.9% of those with low CAS adherence^32^  The CAS correlated with its component measures, but most strongly with VAS (r=0.83) and more strongly at 12 than 6 months:  1. Pharmacy refill (r=0.62 at 6 mo and 0.72 at 12 months)  2. Four-day recall (r=0.77 at 6mo and 0.71 at 12 mo)  3. VAS (r=0.83 at 6mo and 0.83 at 12mo) ^32^ | ^32^ |
| MPR-pill recall | Combination of MPR and pill-taking recall^38^ | Reflects a range of 7 days (self-report) to 6 months (MPR) ^38^ | From routine patient records  Days late for pharmacy refills, dates of refills, dates of appointments for previous 6 months  From the patient through interview  Self-report of adherence over previous 7 days | **Calculation:**  Percentage adherence for both components  **Categorisation:**  Dichotomised  Adherent: if pill recall AND MPR ≥95%^38^  Non-adherent: if pill recall OR MPR OR both <95%^38^ | Combines measures to address limitations of each^38^  More reliable than single time point plasma samples in populations at risk of non-adherence^38^  More feasible than plasma concentrations^38^ |  | Developed by Nguyen et al in 2011 with a 13-item self-report ^114^ and simplified here to one item self-report  The composite measure identified more non adherent patients (21%) than either component alone (self-report: 10%, MPR: 17%), and gave the lowest (most stringent) estimate of adherence (79% vs 90% and 82% respectively)^38^ | ^38^ |
| Composite of ARV concentration and self-report | Used the ARV drug concentration of nevirapine as the main measure of adherence and used self-report to adjust for abnormally low or high drug concentrations (possible drug interactions or metabolic factors) ^55^ |  | From the patient through  Blood sample   - Dried blood spot ^55^   Interview   - Self-reported adherence:  1. Reportedly taking medication regularly regardless of whether they are feeling unwell or not, 2. Not having stopped or forgotten to take this medication is seen as good evidence of self-reported adherence. 3. Use of TB medication^55^ | **Calculation:**  Therapeutic range of NVP: 2-10 ug/mL^55^  Supratherapeutic: >20ug/mL^55^  **Categorisation:**  Excellent adherence: therapeutic NVP + one other ARV  OR supratherapeutic NVP + ≥1 other ARV + self-reported good adherence  OR subtherapeutic NVP +TB medication + self-reported good adherence^55^  Good adherence: non therapeutic NVP + one other ARV detected^55^  Poor adherence: ≤1 ARV detected^55^ | Potentially more accurate than either component alone^55^  Addresses the issues of each component by combining them   - ARV concentration brings objectivity - Self-report adjusts for metabolic and drug interaction effects on the concentration ^55^   TB medication self-report less prone to bias than adherence self-report, and this is the main adjusting factor in the combined model ^55^ | Only developed for nevirapine, which is no longer the drug of choice for first line or any regimen ^55^  May misclassify people as adherent in adjusting their score ^55^ | The combined metric did not change the proportion with poor adherence compared to ARV concentration alone (constant 31%), decreased those with good adherence (24% to 16%) but increased those with excellent adherence (46% to 53%) compared to using the 3 categories by ARV concentration alone ^55^  No relationship between self-reported adherence and presence of ARV medication^55^ | ^55^ |
| Behavioural adherence measure | Composite of self-reported adherence, MPR and tenofovir diphosphate at 12 months^115^ |  | From the patient through interview  3 item self-report (used by Phillips et al^99,100^):  - MISSED: an assessment of the number of days with missed ART doses in the preceding 30 days;  - RATING: a scale rating of how good a job you did taking your medicines in the preceding 30 days and  - FREQUENCY: a scale rating of how often you took your medicines the way you were supposed to in the preceding 30 days  Pharmacy refill dates, pills dispensed  Blood sample^115^ | Unclear how they will be combined | Use of TFV-DP will allow detection of small, clinically significant differences in adherence between groups that may be missed by viral load testing alone ^115^ |  |  | ^115^ |
| Composite of pharmacy refill measures | Composite pharmacy refill metric of attendance and coverage | Reflects a specified period | From routine patient records   - Pickup timing: binary: on time/ late - Pill coverage: binary: 100% vs <100% | **Categorisation:**  Categorical outcome of:  - on time and full coverage  - late but complete coverage  - late and insufficient coverage^20^ |  | Weaker association with viral suppression than pickup timing or pill coverage alone ^20^ | Both late but full coverage: OR 1.23 [1.62-2.20] and late with insufficient coverage: OR 1.39 [1.24-1.55] were associated with viral non-suppression (>400 copies/mL) compared to on time and full coverage as a reference^20^  Stronger association with viral outcomes than self-reported adherence by single item (OR 1.14) ^20^ | ^20^ |
| CD4 stratified by MCV | MCV as a measure of adherence to zidovudine or tenofovir and CD4 as an immunological proxy of adherence^67^  Combined by stratifying CD4 change by MCV change | Reflects previous six months ^67^ | From patient through blood sample ^67^  MCV at visit: Categorised as <80fL, 80-100fL and >100fL^67^  CD4 at visit: Categorised as >200 cells/mm3, 101-200cells/mm3, 51-100cells/mm4 and ≤50cells/mm3^67^ | **Calculation:**  MCV difference = MCV at 6 months - MCV at baseline  CD4 difference = CD4 at 6 months - CD4 at baseline  Expected CD4 change = 100cells/mm^3^/year x duration of treatment in years  Stratified dichotomised change in CD4 by dichotomised change in MCV  **Categorisation:**  MCV difference: dichotomised Adherent: ≥14.5fL^67^  Non-adherent: <14.5fL^67^  CD4 difference: dichotomised Adherent: ≥expected^67^  Non-adherent: <expected ^67^ | MCV a good predictor in the most common regimen (TDF) ^67^  MCV and CD4 have high NPV - rule out ^67^  Based on relationship between labs and ARV effects   - CD4 is expected to increase at least 50 cells/mm3 in the first 6 months and 100 cells/mm3 per year on ART) ^67^   Zidovudine causes an increase in the MCV within 4 weeks of initiation^67^ | Dependent on regimen- can use for tenofovir or zidovudine-based regimens ^67^ | CD4 change stratified by MCV change was the strongest predictor (compared to CD4 or MCV alone) of unsuppressed VL: combination of CD4 change (<expected) and MCV (<14.5fL) at 6 months after ART initiation was a marker of poor adherence by VL reference with moderate sensitivity (86.5%) and high NPV (94.3%) but low specificity (37.3%) and PPV (18.8%)  Higher sensitivity for viral suppression in tenofovir (98%) vs zidovudine (77%) regimens, but higher NPV for zidovudine (95%) than tenofovir (81%) regimens^67^ | ^67^ |
| On time – pill count | Measure of adherence by pill count and late for visits | Reflects a specified period | From the patient   - Pill count   From routine data  Date of visit and scheduled/expected visit | **Categorisation:**  Adherent: ≥95% doses by pill count AND ≥3 days late for schedules visit^116^  Nonadherent: <95% doses by pill count OR >3 days late for schedules visit^116^ |  |  |  | ^116^ |

## Table 8: Measures of active self-management with detailed information on their use

| Measure | Description | | Data required | | Evaluation | | | Ref |
| --- | --- | --- | --- | --- | --- | --- | --- | --- |
|  | Definition | Pattern and duration of measurement | Data collected and source | Processing: presentation, scoring, calculation, categorisation and interpretation | Strengths | Limitations | Evidence (for association with clinical outcomes/ other elements of engagement/ validity) |  |
| Active involvement | | | | | | | | |
| Action planning | Measure of a patient’s planning for their ART management | Reflects the following 2 months | From patients through interview  4 items over the next 8 weeks rated from strongly disagree (1) to strongly agree (5)   1. Looking at the next 8 weeks, I know exactly for each day how often I will take my medicine (strongly disagree – strongly agree) 2. Looking at the next 8 weeks, I know exactly for each day at what time I will take my medicine (strongly disagree – strongly agree) 3. Looking at the next 8 weeks, I know exactly for each day where I will take my medicine (strongly disagree – strongly agree) 4. Looking at the next 8 weeks, I know exactly for each day how (i.e. with or without food and fluid) I will take my medicine (strongly disagree – strongly agree) ^117^ | **Calculation:**  Average responses to items to get final score /5 ^117^ |  | Seen as a mediator of intentions on behaviour but the results did not corroborate this^117^ | From Luszczynska and Schwarzer 2003 ^118^  Action planning was not significantly associated with adherence and did not mediate the effect of intention on adherence behaviour ^117^ | ^117^ |
| Health-seeking behaviour | Measures the way a patient intends to engage | Reflects a general state | From the patient through interview  Was there ever a time that you wanted to go to the clinic and did not go? |  |  | Does not measure actual actions^119^ | Associated with generalised self efficacy: for each additional point increase on the GSE, the odds that a woman would report not having visited a clinic when she wanted to do so decreased by one third (OR 0.67, CI 95%: 0.46, 0.98; p = .04) ^119^ | ^119^ |
| Patient activation (PAM-13 scale) | Self-reported activation as a measure of engagement^120^  Evaluates:  --Patient activation (knowledge. skills and confidence in managing own care)  --Self-efficacy (belief in ability to maintain healthy lifestyle)  --Readiness to utilize formal Health Services  --Actual utilization of formal health care (taking action) | Reflects a current state | From the patient through interview  Patient activation measure as a measure of engagement  13 item scale scored on a 4 point Likert scale from 1 (disagree strongly) to 4 (agree strongly) (no neutral, and an N/A option)  1. I am the person who is responsible for taking care of my health.  2. Taking an active role in my own health care is the most important thing that affects my health.  3. I am confident I can help prevent or reduce problems associated with my health.  4. I know what each of my prescribed medications do.  5. I am confident that I can tell whether I need to go to the doctor or whether I can take care of a health problem myself.  6. I am confident that I can tell a doctor or nurse concerns I have even when he or she does not ask.  7. I am confident that I can carry out medical treatments I may need to do at home  8. I understand my health problems and what causes them.  9. I know what treatments are available for my health problems.  10. I have been able to maintain lifestyle changes, like healthy eating or exercising.  11. I know how to prevent problems with my health  12. I am confident I can work out solutions when new problems arise with my health.  13. I am confident that I can maintain lifestyle changes, like healthy eating and exercising, even during times of stress ^120^ | **Calculation:**  Total PAM score = the raw score is divided by the number of items answered (excepting non-applicable items) and multiplied by 13 then calculated as a percentage^120^  **Interpretation:**  Higher PAM scores indicate higher patient activation and greater engagement^120^ |  |  | Developed by Hibbard et al in 2004^121^ibbardH | ^120^ |
| Patient-provider interaction quality | Comprehensive measure of interactions between patients and providers through scoring of coded consultations  1. **Provider factors in interaction**  1.1 Provider verbal dominance  1.2 *Facilitation and patient activation*  1.3 Provider positive affect  1.4 Patient centredness  1.5 *Provider information gathering*  2. **Patient factors in interaction**  2.1 *All patient question asking*  2.2. Patient activation and engagement  2.3 *Patient positive affect*  3. **Global affect**  3.1 *Provider global affect* (positive affect, dominance and assertiveness, interactivity)  3.2 Patient global affect (positive affect, interactivity)^122^ | Cross sectional measure and reflects a particular consultation  Score can be repeated to evaluate a trend ^122^ | Data collected from patient consultations  Consultations are audio-taped and transcribed, then coded.  For **provider and patient factors** in the interaction, each thought expressed by the patient or provider is coded by 37 mutually exclusive and exhaustive codes.  These are divided into elements of:   - Socioemotional communication: positive, negative, emotional, partnership building, social exchanges - Task-focused communication: asking questions, giving instruction and direction, giving information   Further categorised into 4 primary functions of the medical visit   - Data gathering - Patient education and counselling - Responding to patient emotions - Partnership building   For **global affect** the affect of the provider and patient is subjectively rated in each domain by the scorer on a scale of 1 (none/low) to 6 (high)^122^ | **Scoring:**  Roter Interaction Analysis System used to code^122^  **Calculation:**  A score for provider and patient factors in the interaction is calculated by the frequency of utterances falling into each of the 37 codes^122^  A score for global affect is calculated /6^122^  **Interaction:**  Higher scores relate to better quality interactions^122^ | The coding of patient and provider factors in the interaction is fairly objective ^122^ | Affect coding is very subjective ^122^ | The Roter Interaction Analysis System was developed by Roter and Larson in 2002 ^123^  All three domains associated with patient empowerment (as measured by the difference between an empowerment intervention group and the less empowered control group, with doctors as the providers). Each item was associated with a positive trend, with the following having statistically significant associations^122^:  1.2 Facilitation and patient activation - adj score difference 1.19 [0.39-1.99]  1.5 Provider information gathering - adj score difference 2.96 [1.42-4.50]  2.1 All patient question asking - adj score difference 0.48 [0.11-0.85]  2.3 Patient positive affect - adj score difference 2.08 [0.79-3.36]  3.1 Provider global affect  - positive affect - adj score difference 0.60 [0.08-1.11]  ^122^ | ^122^ |
| Self care and self-management | | | | | | | | |
| Measures of Drug Self-Management Scale (MeDS) | Measure of whether the patient manages their medicine or not and measures risky self-management behaviours | Reflects a general but current status | From the patient through interview:  Self-reported medication adherence was measured using MeDS  12-question survey  Binary coding of responses: each answer weighted either 0 (no/disagree)or 1 (yes/agree) ^124^.  1. Did you forget to take your (insert drug name) at any time last week?  2. In the past month have you stopped taking (insert drug name) for any reason without telling your doctor?  3. I often forget to take my medicine.  4. I am organized about when and how I take my medicines.  5. I have a hard time paying for my medicines.  6. The print instructions on my prescription bottles are confusing.  7. Having to take medicines worries me.  8. I often have a hard time remembering if I have already taken my medicine.  9. I do not take my medicines when I am feeling sad or upset.  10. My medicines disrupt my life  11. When my medicine causes minor side effects, I stop taking it.  12. The idea of taking medications for the rest of my life makes me very uncomfortable^124^ | **Calculation:**  Scores were added up for a total of 12.  **Categorisation:**  Adequate medication drug self-management: score ≥10  Inadequate drug self-management: score <10 ^124^ | The MeDS scale considers missed doses within the past week, as well as risky self-management behaviours, making it a better indicator of adherence than self-reported adherence ^124^.  MeDS considers patient behaviours that contribute to maintaining adherence over time: evaluates whether the patient understands directions, the ability to monitor side effects, and how the patient feels about taking medications long-term ^124^.   Can be used as a quick assessment to direct intervention efforts effectively ^124^. | The MeDS questionnaire identifies patients at risk for poor adherence, but needs to be coupled to an objective adherence study instead of self-reports to measure a direct relationship to adherence ^124^ | Developed and validated by Baily et al in 2015 ^125^  In a Kenyan population, the overall standardized Cronbach’s α coefficient was 0.64, and deletion of any item did not change Cronbach’s α significantly.  Factor analysis showed two extracted factors having eigenvalues >1 after varimax rotation.  Questions had factor loadings of at least 0.3 for Factor 1, except Questions 2, 8 and 12, and can explain 18.0% of the variance.  Factor 2 showed oppositely significant factor loadings and can explain 8.7% of the variance ^124^.  Less than half of participants that are considered adherent by self-reported missed doses are actually classified by MeDS as having adequate self-management behaviour ^124^. | ^124^ |
| Self-reported ART self-management | Self-reported factors that contribute to self-management | Reflects a general but current status | From the patient through interview  ART management questions:   1. Self-reported pill burden: <4/ ≥4 pills per day 2. Age when the adolescent learned his/her positive HIV status: 10-14, ≤9 or 15-19 years old 3. Whether the adolescent attended the clinic by him/herself in the past three months: yes/no ^40^ |  | Self-management questions based on previous qualitative work ^127^ | Evaluated in adolescents | Self-management was not associated with ≥48 hour treatment interruption or six-month MPR ^41^  Only the item: 'attends themselves' was significantly associated:  Those who attended themselves had a 2.23 greater odds of poor adherence by MPR [1.32-4.14] than those who attended with a caregiver ^41^ | ^41^ |
| Practice of self-management | Self-management outcome is the practice of self-management by the patient, comprised of:  - Daily physical health practice  - Activating social support  - Living with chronic HIV  ^128^ | Reflects a general state | From the patient through interview  20 items in 3 domains with Likert scale from 0 (not applicable) to 3 (all of the time)  **Domain 1: Daily Health Practice**   1. Staying physically active (exercising) is an important part of my HIV management strategy 2. I have been successful at staying physically active (walking, exercising, stretching, weight lifting, physical work) 3. Spirituality/Religion is my motivator to manage HIV 4. I have been changing some aspect of my health to better manage HIV (ex: taking medication, exercising, reducing stress) 5. I have been successful at achieving my health goals 6. I modified my diet to better manage HIV (vegetables, fruits, natural ingredients) 7. Even with all of my family responsibilities I had have enough time to take care of my health needs 8. I set aside personal time to do things I enjoy 9. My job responsibilities help me to take care of my health 10. Educating others about HIV helps me stay in control of HIV (working as a counsellor, advocating for safe sex) 11. When I was stressed out I did positive things to relieve the stress (exercising OR journaling OR joining a group) 12. I was able to control (or manage) HIV symptoms and medication side effects^128^   **Domain 2: Resource mobilisation for HIV Self-Management**   1. When I feel overwhelmed, I find that talking to my counsellor or attending support groups is very helpful. 2. Attending support groups is an important part of HIV Management strategy. 3. I have been attending support groups because I found that listening to someone’s testimony or personal story motivates me to take better care of myself^128^ <not giving you a cut and dry answer of whether people do or don’t, but what they would do but that isn’t always reflective of behaviour>   **Domain 3: Chronic Nature of HIV and Self-Management**   1. I have accepted that HIV is a chronic (or life-long) condition that can be managed 2. Managing HIV is a number one priority for me 3. HIV has been my motivator to take better care of myself 4. I call to make appointments with my HIV doctor when I needed to (change in symptoms, problems with meds, new health concern)   My HIV doctor and I have a good relationship^128^ | **Scoring:**  Each item is scored 0 to 3  **Calculation:**  Sum the individual scores to get a total self-management score /60 🡪 converted to a score /3 ^128^  **Interpretation:**  Higher score means better self-management ^128^ |  | Social desirability bias^128^ | Developed by Webel et al in 2012 for use in female HIV patients in the USA with a Cronbach alpha of 0.72-0.86 ^129^  Cronbach alpha score ranging from 0.62-0.77 in Ethiopia ^128^  19.7% of the variance in self-management practices in this sample was explained by a multivariate model consisting of:  1. Self-management process indicators  - Knowledge of ART score  - Self-efficacy  - Self-regulation: set goal for self-management  - Social facilitation: disclosed HIV status  2. Patient perceptions of interventions to enhance the practice of self-management by patients (counselling, support of disclosure and support groups)  3. Characteristics: gender, educational level, income, job status, rural location and knowledge of HIV stage. ^128^ | ^128^ |
| Composite self-management outcome | Measurement of self-management outcomes, comprised of:   1. Self-efficacy   Health status^130^ | Health status reflects the previous week, and self-efficacy reflects a general status  ^130^ | From the patient through interview  Self-management comprised of two domains: self-efficacy and health status  **Domain 1: Self-efficacy**  The scale covered domains such as symptom control, health-modifying behaviour and tasks performed to minimise the need for medical attention  Six item scale each item scored on a 10 point visual numeric scale from 1 (not confident at all) to 10 (totally confident)   1. How confident do you feel that you can keep the fatigue caused by your disease from interfering with the things you want to do? 2. How confident do you feel that you can keep the physical discomfort caused by your disease from interfering with the things you want to do? 3. How confident do you feel that you can keep the emotional distress caused by your disease from interfering with the things you want to do? 4. How confident do you feel that you can keep any other symptoms or health problems you have from interfering with the things you want to do? 5. How confident do you feel that you can do the different tasks and activities needed to manage your health condition so as to reduce your need to see a doctor? 6. How confident do you feel that you can do things other than just taking medication to reduce how much illness affects your everyday life?^130^   **Domain 2: Health status in four domains**   - Perceived illness intrusiveness   Perceived illness intrusiveness - nine items on a five point scale from 0 (none of the time) to 5 (all of the time)  During the past week, how much has your illness(es) and or its treatment interfered with:   1. Your feeling of being healthy? 2. The things you eat and drink? 3. Your work i.e. job, housework, chores or errands? 4. Your financial situation (income and expenditure)? 5. Your relation & social activities with family? 6. Your relation and social activities with others (friends, neighbours or groups)? 7. Your hobbies or recreation activities at your free time (e.g. football, visiting friends, singing, dancing) 8. Your involvement in community activities? (e.g. self-help groups, cultural and public events) 9. Your religious or spiritual activities?  - Patient symptom rating ^130^   Patient symptom rating - 5 items rated from 0 (not a problem) to 10 (severe problem)   1. The extent you are affected by FATIGUE in the past week. 2. The extent you are affected by PAIN in the past week. 3. The extent you are affected by SHORTNESS OF BREADTH in past week. 4. The extent you are affected by STRESS in past week. 5. The extent you are affected by SLEEPING PROBLEMS in past week.  - Self-rated general health ^130^   Self-rated general health - 1 item rated from 1 (excellent) to 5 (poor)  In general, would you say your health is: (circle one):  1)Excellent; 2) Very good; 3) Good; 4) Fair; 5) Poor   - Quality of life ^130^   Quality of life - 10 point visual scale  Overall, how would you say was your quality of life in the past week. Looking at the graph provided, with 0 your life is not very good and 10 being your life is very good or excellent. How would you rate your overall QUALITY OF LIFE in past week?^130^ | **Interpretation:**  Self-efficacy: higher scores indicate more confidence in managing life with the condition ^130^  Health status:  Perceived intrusiveness: higher scores indicate greater limitation to activities and roles  Symptoms: higher scores indicate worse symptoms  General health: higher scores indicate worse health (reverse coded)  Quality of life: higher scores indicate better quality life  ^130^ |  |  | Adapted from a chronic disease self-management tool by Lorig and Laurent in 2007 ^131^  Self-efficacy was significantly associated with all aspects of health status  (Higher self-efficacy was associated with lower illness intrusiveness (r= -0.516), lower symptom severity (fatigue: r= -0.396 and emotional distress/ stress: r= -0.363), better health status (r = -0.599 -score is reverse coded) and better quality of life (r = 0.457) )  ^130^ | ^130^ |
| Adolescent HIV Self-management scale (AdHIVSM scale) | Measurement of self-management activities across self-care, self regulation, social facilitation, empowerment and knowledge, specific to adolescents | Reflects a general but current status | From the patient through interview^132^  Final AdHIVSM scale has 35 items covering processes and behaviours (proximal outcomes)  35 item questionnaire, each rated from 1 (poor self-management) to 4 (good self-management) ^133^  that loaded onto 5 components  1 - Believing and Knowing  2 - Goals and facilitation (self-regulation and social facilitation)  3 - Participation/ Activation (communication, active participation)  4 - HIV biomedical management (knowledge of HIV management related information - own VL and ARV names)  5 - Coping and self-regulation (coping with stigma and overcoming barriers) ^132^ | **Calculation:**  Total score: sum responses for each item and divide by the number of items in the subscale ^132^  **Interpretation:**  Higher scores reflect greater levels of HIV self-management ^132^ | Instrument includes all the theoretical components of self-management ^132^  Comprehensive tool to measure self-management - extends beyond medical self-management only (compared to other tools) ^132^  Supports IFSMT theory and can identify which aspects of self-management patients find challenging ^132^ | Younger adolescents may have answered without truly evaluating what the question required, suggested by the cognitive interviews ^132^  Scale does not assess risk behaviours ^132^  Validated for use in adolescents not adults ^132^ | Psychometric evaluation  Acceptable reliability = 0.84  Acceptable stability = 0.76  Good model fit supporting structural validity: RMSEA = 0.052, RMR 0.065, Comparative fit index (CFI) = 0.9  Reliability: overall 35 item scale had a Cronbach's alpha = 0.839^132^  Subscales 1/1-1/3 had good reliability (Cronbach alpha 0.761, 0.708 and 0.715 respectively), subscale 1.4 had minimally acceptable reliability (0.651) and subscale 1.5 had unacceptable reliability (0.547) ^132^  Hypothesis-testing construct validity (convergent validity - with constructs that it should be measuring?)   - Treatment self-efficacy: associated significantly - Resilience: associated significantly - Health related QoL: associated significantly^132^   Context:   - Total strengths: associated significantly - Symptoms: associated significantly - Stigma: associated significantly^132^   Criterion-related validity (expected associations with outcomes)   - Adherent vs non adherent: adherent patients had moderately higher but significant self-management scores   Participation was NOT associated with adherence   - Suppressed VL vs unsuppressed VL: virally suppressed patients had slightly higher but significant self-management scores^132^   Self-management is associated with   - Adherence   High self-management associated with an increased likelihood of being adherent  Negative correlation of higher self-management with poor adherence: r = -0.249 (-0.34 in the least squares structural model)  Subscale with the strongest negative association was participation with non-adherence   - viral outcomes (medical outcomes)   High self-management associated with an increased likelihood of being virally suppressed <50 copies/mL  Seems to be mediated by adherence  Correlation with log VL: r= 0.17  Association between risk behaviour and VL   - health related quality of life (psychosocial outcomes)   High self-management associated with an increased likelihood of good QoL  Correlation coefficient r=0.450  Subscale with greatest association was goals and facilitation  Non adherence also had a moderate negative association with QoL^133^ | ^132^  ^133^ |
| HIV Self-Management Inventory (SMI) | Self-reported self-care practices as a measure of self-management^121^ | Reflects a general but current status | From the patient through interview  22 item scale in three domains - yes/no  - assessed in three domains (physical health maintenance, psychological/emotional self-care and social/ spiritual self-care)  **Domain 1: Physical self-management:**  1. Healthy diet / 2. Regular exercise (at least 3 times a week)/ 3. Relaxation and stress relief / 4. Getting adequate sleep (up to 8 hours at night) / 5. Taking vitamins, supplements and immune-boosters / 6. Taking antibiotics (self-prescribed) / 7. Taking ART (self-prescribed) / 8,9,10,11. Refraining from risky behaviour (e.g. unsafe sex, drugs, alcohol, smoking) / 12,13. Care seeking from health facilities/care providers when unwell  **Domain 2: Psychological/emotional self-care:**  14. Positive attitude (e.g. optimistic outlook) / 15. Positive affective state (trying to be joyful despite diagnosis) / 16. Self-reflection (careful thoughts about own behaviour and actions)  **Domain 3: Social/relationship self-care:**  17,18. Positive social relationships (seeking social support; spending time with friends) / 19. Asking for help when overwhelmed emotionally / 20, 21,22. Spiritual (e.g. meditation, praying and accepting the things that cannot be changed) ^121^ | **Calculation:**  Calculate total score ^121^  **Interpretation:**  Higher scores indicate higher self-management ability ^121^ |  |  | Cronbach’s coefficient of 0.68-0.73 ^121^ | ^121^ |
| Revised self-care symptom management strategies (SSC- HIVrev) | Self-care symptom management strategies as a measure of health behaviour ^134^ | Reflects a general but current status | From the patient through interview  Measures intensity and frequency of common signs and symptoms of HIV with  Answer options: do not have the problem/ mild/ moderate/ severe  **PART 1** - 45 HIV related physical and psychological symptoms in 11 clusters  **PART 2** - 19 HIV related symptoms without a cluster  **PART 3** - 8 gynaecological signs and symptoms  Used to identify change in symptoms and signs over time ^134^ |  |  |  | Self-care symptom scale described by Holzemer et al 2001 ^135^, validated by Dlamini et al in 2009 in Southern Africa with a Cronbach of 0.96 ^136^ | ^134^ |
| Self-care practices | Evaluation of the self-care activities that a patient performs | Reflects a general but current status | From the patient through interview  Structured questionnaire on Self-care practices, which comprised:  - Eating a balanced diet  - Adequate rest and sleep  - Physical exercises  - Effective stress management through counselling  - Medication adherence  - Focused antenatal care (ANC): timing of confirmation of pregnancy, gestational age at booking, ANC visits (</>4 times), PMTCT visits, treatment for anaemia  - Prevention of mother to child prevention of HIV (PMTCT)  - Safe sexual practices as routinely recommended in Zimbabwe ^137^ | **Categorisation:**  Very good  Good  Poor  Very poor^137^ |  |  | Good self-care practices were correlated with good maternal outcomes and poor self-care was associated with poor maternal outcomes (CD4, weight gain, anaemia, opportunistic infections and haemorrhage)  - Pearson's correlation coefficient r= 0.317, p<0.01  - Regression coefficient 0.165  - R2 0.10 ^137^ | ^137^ |
| Self-care management | Self-care management on treatment ^138^ | Reflects a general but current status | From the patient through interview  SECTION C of a self-care questionnaire = self-care management on treatment regimen   1. Clinic attendance + reasons 2. Adherence + reasons 3. Other treatments taken with ART + reasons 4. Alcohol use ^138^ |  |  | Tool not standardised - reduced robustness of information generated ^138^  Potential for recall bias and self-presentation  cross sectional study design precludes conclusions on causation ^138^ | Questionnaire developed from literature search + expert review. Piloted on 42 PLWH from another clinic. Reliability coefficient (Cronbach's alpha)= 0.81 ^138^ | ^138^ |
| Three-item self-regulation | Individuals managing their engagement to achieve a change in health behaviour ^128^ | Reflects a general but current status | From the patient through interview  Three items with yes/no answers   1. Do you have plan for self-management of emotional distress? 2. Are you familiar on how to manage your HIV illness related symptoms? 3. Have you set a goal in the process of your HIV therapy?^128^ | **Calculation:**  Total/3 ^128^ | Simple | Social desirability bias^128^ | Developed by Areri et al in 2020 using existing literature to assess Individual Family Self-Management Theory domains not covered by existing tools. These were developed as 'yes/no' items and piloted on a small sample^128^ | ^128^ |
| Four-item self-regulation | Measure of the regulation of ART management processes  Seen as a mediator of intentions to behaviour on adherence^118^ | Reflects a general state | From patients through interview  Self-regulation - 4 item scale rated never (1) to always (5)   1. I closely monitor whether I take all my ARVs every day (never – always) 2. I make sure that I get new ARVs at the clinic before my ARVs are finished (never – always) 3. If I notice that I have not taken my ARVs or I have taken them too late, I think about what the reason for that was and how I can prevent that from happening again (never – always) 4. I watch carefully that I take all my ARVs every day (never – always) (Banas *et al.*, 2017 | **Calculation:**  Average responses to items to get final score /5 ^118^ |  |  | Three items from Bruin et al 2012 ^139^ and 6 from Lyimo et al 2012 ^140^, but only 4 items (focused on planning and monitoring of medication intake) used as the full 9 item scale was not unidimensional on psychometric analysis ^118^  Self-regulation predicted adherence by electronic monitoring: B = 0.234 but only explained a small proportion of the adherence: R^2^ 14.7% ^118^  The indirect effect of intention on adherence (by electronic monitoring) was mediated by self-regulation processes, indicating that participants who had a stronger intention to adhere were also more likely to monitor their medication intake, plan to pick up a new dose of medication before they ran out, or reflect on the reasons for missed doses in order to maintain good adherence in the future. These self-regulatory processes were the main predictor of adherence in this study^118^ | ^118^ |
| Social facilitation | Social facilitation is negotiating collaboration, support and the facilitation of engagement in recommended behaviours by those with social influence, such as healthcare providers, family and peer networks ^128^ | Reflects a general but current status | From the patient through interview   1. Have you joined networks of people living with HIV? 2. Do you think you have adequately linked to social/ peer network? 3. Did the health care providers provide you information on ART? 4. Have you disclosed your HIV status?   If “No” to question 4, what is the reason/s for not disclosing? (more than one is possible)   1. Fear of stigma and discrimination 2. Considered not important 3. Other (Specify)…   Do you have reminders for your HIV management?^128^ | **Calculation:**  Total score /5 ^128^ |  | Measures components that are not in the direct control of the patient - social support requires the input of multiple groups and depends on the availability of social services ^128^  Social desirability bias ^128^ | Developed by Areri et al in 2020 using existing literature to assess IFSMT domains (Individual Family Self-Management Theory) not covered by existing tools. These were developed as 'yes/no' items and piloted on a small sample ^128^ | ^128^ |
| Coping | Measures the frequency and range of coping strategies used ^23^ | Reflects a general but current status | From the patient through interview  42 item questionnaire: assess:  Frequency of use of coping mechanism: 1 (not at all) - 4 (most of the time)  Which coping mechanisms used:  - Active coping (13 items)  - Avoidant coping (16 items)  - Social/spiritual coping (13 items)^23^ |  |  |  | 43 item survey already validated ^141,142^  four additional items chosen based on cognitive interviewing and formative findings:  - Active coping: Cronbach's alpha 0.83 ^23^  - Avoidant coping: Cronbach's alpha 0/79 ^23^  - Social/spiritual coping: Cronbach's alpha 0.78^23^  Five items removed in total to produce a 42-item survey | ^23^ |

## Table 9: Measures of multi-dimensional engagement with detailed information on their use

| Measure | Description | | Data required | | Evaluation | | | Ref |
| --- | --- | --- | --- | --- | --- | --- | --- | --- |
|  | Definition | Pattern and duration of measurement | Data collected and source | Processing: presentation, scoring, calculation, categorisation and interpretation | Strengths | Limitations | Evidence (for association with clinical outcomes/ other elements of engagement/ validity) |  |
| Composites of different elements of engagement | | | | | | | | |
| Engagement score (retention, adherence and clinical status) | A score measuring engagement consisting of patients' clinical status (age, gender, WHO stage, platelet count, MCV), retention (days late for ART visits) and VAS adherence^84^ | Reflects previous 6 months | From routine data and the patient  - Baseline characteristics  a. Age  b. Gender  c. WHO stage  - Variables at 6 months on ART  d. Platelet count  e. Mean cell volume (MCV) increase  f. Retention: days late for ART visit  g. Adherence: VAS ^84^ | **Scoring:**  Composite score inclusive of:  a. Age > 35: +1  b. Male gender: +1  c. WHO stage III/IV: +1  d. Platelet count < 150 cells/mm3 at 6 months: +3  e. MCV increase <14.5 fL at 6 months: +1  f. ≥7 days late for ≥2 ARV visits at 6 months: +2  g. VAS adherence <90% at 6 months: +2  (thresholds optimised the diagnostic accuracy)^84^  **Calculation:**  Sum the score /11  **Categorisation:**  The optimal diagnostic accuracy was at a threshold of ≥5 ^84^  Engaged: score <5 (unlikely to be viraemic)  Not-engaged: score ≥5 (likely to be viraemic)^84^ | The first 6 months on ARVs is a particular risk and this score identifies patients at risk of early VL failure who could benefit most from interventions to improve adherence ^84^  Clinical markers are already routinely collected: data is accessible and collection does not add costs^84^  Can be calculated in real time and direct management decisions ^84^  Nurses were able to incorporate this risk assessment tool into their clinical assessment of patients at 6 months on ART - collecting the data, calculating the score and interpreting the risk ^84^ | Only applicable at 6 months on ART ^84^  Relies on completeness of routine data at 2 time points^84^  Need to be able to follow up the prediction with an intervention to make it useful - need to have adherence counselling available ^84^  This score would increase the number of patients requiring adherence counselling by 3-4 times – increase burden on staff^84^  Had a high rate of false positives, that would use resources for an unwarranted intervention, and a high rate of false negatives, however these would have been caught at the routine VL^84^ | Those with a total risk score ≥5 were more likely to have poor viral load suppression (47/136; 34.6%) at 6 months (RR 1.42 [0.94–2.15]) than those with a score <5 (25/103; 24.3%)^84^  Predicts viraemia at 6 months on ART with moderate sensitivity (65% [53.8-75.2]) but low specificity (47% [39.3-54.3]) specificity. There was a high rate of false positives and false negatives, meaning many patients were misclassified and would have received the incorrect intervention ^84^  Reducing the threshold for the score increased the sensitivity: score ≥4 still had moderate sensitivity (76.4%)^84^.  Performed better at predicting viraemia (sensitivity 65%) than VAS (25%) , SMAQ (27%) or a composite adherence measure (18%) alone^84^ | ^84^ |
| Composite measure of engagement (adherence and retention) | Composite of self-reported adherence by AACTG, retention by 60 day-visit gap and viral suppression (<1000 copies/mL)^23^ | Reflects a specified time period | From patient through interview   1. Questionnaire: number of days over previous month where medication was missed (AACTG instrument) ^23^   From routine patient records   1. Visit dates^23^   From patient through blood sample^23^   1. VL | **Categorisation:**  Engaged: adherent AACTG score^23^ (missed no doses^44^ or 100% adherence^37^) AND retained by visit gap (≤60 days between 2 visits) AND virologically suppressed (VL <1000 copies/mL)^23^  Not engaged: non-adherent by AACTG (missed ≥1 dose^44^ or <100%^37^) OR not retained by visit gap (>60 days between visits) or viral failure (≥1000 copies/mL)^23^ |  |  |  | ^23^ |
| Composite outcome of retention and viral suppression | Must be both retained in care and virally suppressed to be considered engaged  Need to define each component individually:   - Retention: e.g. 12 months retention (window 9-18 months) post set time point^14^ (e.g. postpartum or post initiation) - Viral suppression: e.g. VL<50 copies/mL^14,143^ or < 200 copies/mL ^57^ | Reflects a specified time point, e.g. 12 months | From routine patient records  Evidence of interaction with the health system  e.g. visit date^116^ + blood sample for viral load^14,143^ | **Categorisation:**  Engaged: both retained in care and virally suppressed^143^ | A novel and robust primary endpoint incorporating both healthcare utilisation (measured by retention) and VL as the key biomarker of interest in HIV treatment^14^ | Will always reflect the VL suppression rate – added complication for no additional information | Composite outcome produced a lower estimate of successful outcome (66%^14^ and 83%^143^) compared to the retention component (76%^14^ and 86%^143^) and was the same as the VL component (66%^14^ and 83%^143^). | ^14^  ^143^  ^57^  ^116^ |
| Engagement trajectory (adherence and retention) | Engagement measured by a combination of adherence (measured by MPR) and retention (measured by point retention with a cut-off of 90 days late for a visit) trajectories^10^ | Reflects a specified time period | From routine patient records  a. Pharmacy refill dates, number pills prescribed  b. Visit dates^10^ | **Calculation:**  Calculate estimated trajectories for MPR adherence and LTFU retention simultaneously and combined into one output^10^  MPR adherence = # days with ART in possession/# days in interval  LTFU did not censor patients - they remained under observation until they met one of the 3 censoring criteria: death, transfer or database closure ^10^  Inverse probability weights were applied to all analyses to account for updated outcomes^10^ ascertained from tracing a random subset of patients lost to follow-up^144^  **Categorisation:**  Adherence: continuous variable ranging from 0-100%  Retention: dichotomised  In care: not LTFU  Not in care: LTFU ^10^  6 categories of engagement:   1. consistently high adherence and retention 2. early nonadherence but consistent retention 3. gradually decreasing adherence and retention 4. early LTFU with later reengagement 5. early LTFU with- out reengagement 6. late LTFU without reengagement^10^ | Provides a more comprehensive and nuanced understanding of patient engagement than more traditional metrics^10^  Trajectories (reflective of patient behavioural phenotypes) more associated with mortality outcomes than patient demographics, which can better identify patients at higher risk o poor outcomes and reveal when in follow-up time those vulnerabilities emerge^10^  The identified longitudinal patterns of engagement behaviour remained consistent across subpopulations stratified by baseline patient characteristics^10^ | Trajectory groups are not necessarily intrinsic properties and only represent attempts to classify patients based on the available data ^10^  Susceptible to misclassification error if the component data (MPR and LTFU) is not accurate^10^ | The model was evaluated for adequacy and fit:  - Good model fit: group average posterior probability > 0.7 for each group (ranged 0.893 - 0.975)  - Odds ratio for correct classification >5 for each group (ranged 22 - 412)  - Entropy >0.8: 0.957 ^10^  The trajectory analysis revealed 6 distinct types of engagement based on adherence and retention), which were associated with differential survival   1. consistently high adherence and retention (28.5%), 2. early nonadherence but consistent retention (22.2%), 3. gradually decreasing adherence and retention (21.6%), 4. early LTFU with later reengagement (8.6%), 5. early LTFU with- out reengagement (8.7%), 6. late LTFU without reengagement (10.4%) ^10^   Increased relative risk of mortality for those with poor retention component compared to group (1): consistently high engagement  (4) early LTFU with later re-engagement: adj RR 3.4 [1.2-9.7]  (5) early LTFU without re-engagement: adj RR 6.4 [2.5-16.3]  (6) late LTFU without LTFU: adj RR 4.7 [2.0-11.3] ^10^ | ^10^ |
| Summary score to predict viral failure as a measure of adherence | Summary score made up of adherence factors predictive of viral failure | Reflects current state | From patient  Through interview  Age, ART history, ART adherence (unclear what method)  Blood sample  CD4 and Haemoglobin | **Scoring:**  Summary score made up of factors predictive of viral failure  a. Age <38 years = 1  b. ART duration >2.5 years = 1  c. ART adherence <95% = 1  d. CD4 <200 cells/uL =2  e. Haemoglobin <13g/dL = 1  **Calculation:**  Sum the score /6 | Simple and practical score^67^  Accurately identifies those at low risk of viral failure --> could be used to optimise and rationalise VL monitoring (reduce unnecessary VL testing by up to 65% using a cut-off score of 3, but at the risk of missing a quarter of those with failure) ^67^  Optimised to identify individuals with low risk of failure and be used as a routine test for clinically stable patients at the time VL testing would ordinarily be done, compared to other prediction tools that are optimised to identify patients with a high risk of failure and require trends of results rather than one-off results ^67^ | Calculation of the score is fairly complicated and prone to human error, particularly in a busy clinic ^67^  Saving unnecessary VL comes at the cost of missing those with viral failure ^67^ – if those who were exempted from a VL were automatically eligible at the next routine monitoring, those that were misclassified as low risk would need have a diagnosis of VL failure delayed by 2 years under WHO guidelines and 4 years under Malawian guidelines, which risks development of resistance, onward transmission, morbidity and death ^67^ | Each component associated with viral failure:  a. Age <38 years: OR 3.44 [2.01-5.89]  b. ART duration >2.5 years: OR 2.98 [1.79-4.96]  c. ART adherence <95%: OR 1.76 [1.06-2.94]  d. CD4 <200 cells/uL: OR 5.94 [3.27-10.78]  e. Haemoglobin <13g/dL: OR 2.76 [1.70-4.50]^67^  A threshold of 2 or 3 has the best balance between sensitivity, avoiding unnecessary VLs and missing those that needed a VL, though neither is ideal. Increasing the threshold decreased sensitivity but decreased the proportion needing a test and increased the proportion of failure missed.  - Threshold ≥1: Sensitivity for failure 100% / Specificity 3.7%. AUC 0.77 (derivation set)/ 0.76 (validation set). Averted 4% VL. Missed no failure.  - *Threshold ≥2: Sensitivity 90% AUC 0.77. Averted 27.8% VL. Missed 10% with failure  - *Threshold ≥3: Sensitivity 75.6%. AUC 0.72. Averted 65% VL. Missed 24% with failure  - Threshold ≥4: Sensitivity 38.9%. AUC 0.67. Averted 91% VL. Missed 61% failure^67^ | ^67^ |
| Viral Load Testing Criteria (VLTC) | VLTC made up of:  a. Current CD4  b. Mid upper arm circumference (MUAC)  c. Self-reported treatment interruption ^88^  to predict viraemia as a measure of adherence | Current state reflected | From the patient  Blood sample for CD4 as part of routine care  MUAC using specific MUAC measuring tapes  Through interview: report of any duration of treatment interruption since the last visit: yes/no ^88^ | **Categorisation:**  Adherent: does not meet any of the VLTC  Non-adherent: requires a VL as meets ≥1 of:  1a. CD4<350  1b. MUAC <23 (women)/24 (men) cm  1c. Yes to 'any duration of treatment interruption since last visit) ^88^ |  | ? doesn’t measure engagement, just uses demographics to predict outcome | VLTC was developed from data in Ethiopian health centres by Reeoalu et al in 2017 ^145^  The VLTC detected viral failure with a moderate sensitivity (85% [68-95]) and specificity (60% [55-64]), high NPV (95% [97-99]) but low PPV (12% [10-14%]), and misclassified 15% of patients with a VL>1000 copies/mL^88^. Using the CD4 and ART interruptions only produced lower sensitivity (82%) but better specificity (73%)^88^  The composite score had a higher sensitivity than the individual components alone ^88^ | ^88^ |
| Characterisation of patient stability | Measures retention and clinical stability - characterises the patient state in relation to this^2^  Stability:   1. Patient initiated on ART >6 months prior; 2. Patient not on second-line ART; 3. no ART regimen switch in previous 3 months; 4. Most recent CD4 count >200 cell/mm3, and not less than 50% maximum CD4 to date, and not less than minimum CD4 to date; 5. No current tuberculosis, WHO stage III or IV diagnosis, or drug toxicity documented in the medical record at visit; and 6. Retention: on time to most recent visit (≤28 days late to last pharmacy appointment date).   Patients were defined as unstable at a visit if any of these criteria were not met^2^ | Patient perspective: by time since ART initiation  Programme perspective: by calendar date^2^  Reflects current state | From routine patient records  Visit dates, appointment dates, type of visit (clinician, pharmacy, adherence) ^2^  ART duration  ART regimen  CD4  TB status  WHO stage  Drug toxicity | **Presentation:**   - Identification of patient state - Incidence and prevalence of states - Transition rates between states^2^   **Calculation:**  - Proportion in each state over time (calendar and time since initiation)  - Transition rates (events/100 person years) between each state^2^  **Categorisation:**  State 1: Never stable on ART (including preART and on ART <6 months)  State 2: Lapse in care before stable on ART  State 3: Stable on ART  State 4: Previously stable on ART, currently unstable  State 5: Lapse in care after stable on ART (Lapse in care was defined as >28 days late to last pharmacy appointment date)  State 6: Death ^2^ | Capture the real-world dynamics of patient stability, yielding potentially more accurate estimates of efficiency gains and programmatic needs with DSD application. ^2^ |  |  | ^2^ |

## Table 10: Measures of treatment outcome with detailed information on their use

| Measure | Description | | Data required | | Evaluation | | | Ref |
| --- | --- | --- | --- | --- | --- | --- | --- | --- |
|  | Definition | Pattern and duration of measurement | Data collected and source | Processing: presentation, scoring, calculation, categorisation and interpretation | Strengths | Limitations | Evidence (for association with clinical outcomes/ other elements of engagement/ validity) |  |
| Laboratory results | | | | | | | | |
| Viral suppression | Measures the outcome of engagement | Reflects the previous few months | From routine patient records^5^ or from the patient  Blood sample^70,71,80^ | **Scoring:**  Suppressed: <20^22^, <50^101,133^, <400^5,27,36,60,61,70,80,132,146^ or <1000^77^ copies/mL  Viraemic: 20-999^22^, 50-999^101^, 100-999^8^ or 400-999^84^ copies/mL  Failure: ≥ 400^5,36,60,61,70,80,146^ or ≥1000^21–23,37,50,52,62,64,67,74,88,101,147^ copies/mL | Composite measure of the outcome of engagement | Does not differentiate between elements, and most papers use it as a gold standard for adherence^30,37,49,50,74^ rather than other elements of engagement  Influenced by resistance, which may be found independent of poor engagement  Not clear what period of time VL reflects  Different definitions (same authors will use different thresholds in different papers. ref ^132^ vs ^133^) | As the gold standard, VL has been associated mostly with adherence, but also with measures of retention and self-management | ^17^  ^74^  ^50^  ^37^  ^71^  ^148^  ^36^  ^49^  ^30^  ^61^  ^52^  ^59^  ^80^  ^69^  ^70^  ^5^  ^62^  ^101^  ^64^  ^60^  ^77^  ^55^  ^38^  ^8^  ^14^  ^84^  ^67^  ^23^  ^88^  ^21^  ^27^  ^132^  ^133^  ^22^  ^42^ |
| Sustained low level viraemia | Persistent low level viraemia (defined as two consecutive raised viral loads between 200 and 1000 copies/mL, each six months apart) is used to identify those with poor adherence at risk of virologic failure^148^  Also called ‘VL blips’ | Reflects the period between two visits | From the patient  Blood sample for viral load | **Categorisation:**  Adherent: <2 raised VL  Non-adherent: a raised viral load between 200 and 1000 copies/mL for ≥2 consecutive study visits (1 blip did not count)^148^ | There is a dose-response relationship with viral failure | Strength of association varied by context ^148^ | Persistent low level viraemia increased the risk of subsequent virologic failure with a dose-response relationship (increasing risk with higher VL) ^148^  Strength of association with viral failure varied with threshold and regimen   - VL 200-499: adjusted HR 1.81 [1.08-3.02] compared to VL<200 - VL 500-999: adjusted HR 2.36 [1.52-3.67] compared to VL<200 ^148^ - Stronger association between pLLV 200-499 and viral failure for first-line than second-line patients^148^   Stronger association between pLLV 500-999 and viral failure for second-line than first-line patients^148^ | ^148^ |
| CD4 change | CD4 as an immunological proxy of adherence (CD4 is expected to increase at least 50 cells/mm3 in the first 6 months and 100 cells/mm3 per year on ART) ^68^ | Reflects the period between two CD4 measurements | From the patient  Blood sample for CD4 | **Scoring:**  CD4 at visit: categorised as >200 cells/mm3, 101-200cells/mm3, 51-100cells/mm4 and ≤50cells/mm3  **Calculation:**  CD4 difference = CD4 at visit (e.g. 6 months) - CD4 at baseline  expected CD4 change = 100 x duration of treatment in years  **Categorisation:**  Dichotomised  ≥expected: CD4 difference ≥ expected CD4 change  <expected: CD4 difference < expected CD4 change ^68^ | Has a high NPV - rule out adherence^68^ | Dependent on regimen^68^  CD4 no longer performed routinely for long term monitoring | CD4 associated moderately with viraemia (VL >400 copies/mL): ROC AUC 0.66 [0.58 to 0.73]^43^ and viral suppression within 270 days of initiation with high specificity (82%) but low sensitivity (33%)^68^. Stratifying by MCV increased sensitivity but decreased specificity^68^  Association varied with regimen  - d4T or AZT: sensitivity 76.8%, specificity 49,2%, NPV 94.8%  - TDF: sensitivity 98.2%, specificity 2.4%, NPV 80.8%^68^  Point CD4<350 predicted viral failure with moderate sensitivity (73%[55-87%]) and specificity 81% (77-84%)^88^  Point CD4 <200 cells/uL predicted viral failure: OR 5.94 [3.27-10.78] ^67^ | ^43^  ^68^  ^67^  ^88^ |
| CD4 trajectory | A measure of engagement as those engaged in care and adherent to ART (when indicated) would be expected to display a steady CD4 count increase early in treatment, followed by maintained high levels. Patients displaying different patterns ofCD4 counts (failure to increase, increase followed by decrease) are likely to be experiencing lapses in care or ART adherence^149^ | Reflects a specified period | From routine patient records ^149^ | **Calculation:**  Averaged CD4 in each calendar year to create one CD4 result per year ^149^ |  | CD4 counts are no longer routinely conducted ^149^ | Four-trajectory model had the best fit by Bayesian information criteria, with odds of correct classification compared to group 1 ranged from 4.6 to 19.7^149^  Four trajectories identified:  - LOW STABLE: Consistently low CD4 (21%)  - LOW INCREASING: Low CD4 that increased over time (22%)  - MODERATE STABLE: Moderate CD4 remaining stable over time (42%)  - HIGH INCREASING: High CD4 that increased over time (16%) ^149^ | ^149^ |
| ARV resistance | Measures the development of HIV resistance to ARVs through genotyping | Reflects the current resistance profile and can miss resistance archived by HIV to ARVs that are no longer exerting selection pressure | From the patient through blood or from routine patient records | **Categorisation:**  Presence or absence of resistance mutations  Early resistance: at 6 months after ART initiation or switch^104^  Late resistance: >6 months after ART initiation or switch^104^ | Intermittent adherence indicated by PI resistance mutations (which persist for weeks to months) and absence of M184V (lamivudine and emtricitabine) mutations, which disappear quickly^47^ | Expensive^47^  May be resistant despite good engagement |  | ^42^  ^47^  ^104^  ^35^ |
| Quality of life and health status | | | | | | | | |
| HIV mortality | Measures HIV-associated death as the ultimate outcome of treatment success or failure^10^  Also termed an ‘unfavourable treatment outcome’^19^ | Reflects the current state  Mortality is a competing risk for loss to follow-up and non-retention, and can result in misclassification of retention outcomes^9,15,16,20^ | From routine data, or from tracing studies^10^ | **Categorisation:**  Alive  Dead |  | Does not solely reflect treatment outcome and can be influenced by other factors | Higher instantaneous risk of death associated with lower average adherence:  adj HR: 0.73 [0.66-0.81] and with higher adherence variance:  adj HR 1.45 [1.03-2.06]^150^  The higher the average adherence/ higher trajectory: the greater the effect of the variance on death^150^  Trajectories with intermittent engagement or early or late loss to follow up had a higher RR of death (adj RR 3.4 [1.2-9.7], adj RR 6.4 [2.5-16.3], adj RR 4.7 [2.0-11.3] respectively)^10^ | ^150^  ^10^  ^19^  ^9^  ^15^  ^16^  ^20^ |
| HIV specific quality of life | Measures quality of life specific to the experience of HIV | Reflects the current state | From the patient through interview  34 HIV specific QoL items covering nine dimensions: overall function, life satisfaction (LS), health worries (HW), financial worries (FW), medication worries (MW), HIV mastery, disclosure worries (DW), provider trust and sexual function (SF).  The subscales are scored on a Likert scale rating of 1 (none of the time) to 5 (all the time) during the past four weeks^134^ | **Scoring:**  The final score is transformed into a linear 0–100 scale where 100 is the best score possible^134^ |  |  | HIV/AIDS targeted QoL instrument developed by Holmes and Shea in 1998 ^151^  The Cronbach alpha reliability coefficient was 0.86 at baseline, and 0.96 and 0.95 for two subsequent assessments indicating excellent consistency reliability of the items ^136^ | ^134^ |
| Self-reported health status | Measures the patient’s perception of their health status | Reflects a current state | From the patient through interview  Self- reported health status rated on a five- point scale ranging from very poor to very good^120^ |  |  |  |  | ^120^ |

### References for Tables 6-10

1. Rivet Amico K. A situated-information motivation behavioral skills model of care initiation and maintenance (sIMB-CIM): An IMB model based approach to understanding and intervening in engagement in care for chronic medical conditions. *Journal of Health Psychology*. 2011;16(7):1071-1081. doi:10.1177/1359105311398727

2. Roy M, Holmes C, Sikazwe I, et al. Application of a Multistate Model to Evaluate Visit Burden and Patient Stability to Improve Sustainability of Human Immunodeficiency Virus Treatment in Zambia. *Clinical infectious diseases : an official publication of the Infectious Diseases Society of America*. 2018;67(8):1269-1277. doi:10.1093/cid/ciy285

3. Font H, Rollins N, Essajee S, et al. Retention-in-care in the PMTCT cascade: definitions matter! Analyses from the INSPIRE projects in Malawi, Nigeria and Zimbabwe. *Journal of the International AIDS Society*. 2020;23(10):e25609. doi:10.1002/jia2.25609

4. Orne-Gliemann J, Font H, Maphosa T, et al. Patterns of Attendance at Mother Support Groups in Zimbabwe. The EPAZ Trial (2014-2016). *Journal of acquired immune deficiency syndromes (1999)*. 2017;75 Suppl 2:S216-S223. doi:10.1097/QAI.0000000000001348

5. Sangeda RZ, Mosha F, Prosperi M, et al. Pharmacy refill adherence outperforms self-reported methods in predicting HIV therapy outcome in resource-limited settings. *BMC public health*. 2014;14:1035. doi:10.1186/1471-2458-14-1035

6. Luma HN, Mbatchou Ngahane BH, Mapoure YN, et al. Cross-sectional assessment of three commonly used measures of adherence to combination antiviral therapy in a resource limited setting. *International journal of STD & AIDS*. 2017;28(1):69-76. doi:http://dx.doi.org/10.1177/0956462415627394

7. Mûnene E, Ekman B. Association between patient engagement in HIV care and antiretroviral therapy medication adherence: cross-sectional evidence from a regional HIV care center in Kenya. *AIDS Care*. 2015;27(3):378-386. doi:http://dx.doi.org/10.1080/09540121.2014.963020

8. Peterson K, Menten J, Peterson I, et al. Use of Self-Reported Adherence and Keeping Clinic Appointments as Predictors of Viremia in Routine HIV Care in the Gambia. *Journal of the International Association of Providers of AIDS Care*. 2015;14(4):343-347. doi:10.1177/2325957413500344

9. Rachlis B, Cole DC, van Lettow M, Escobar M. Survival functions for defining a clinical management Lost To Follow-Up (LTFU) cut-off in Antiretroviral Therapy (ART) program in Zomba, Malawi. *BMC medical informatics and decision making*. 2016;16:52. doi:10.1186/s12911-016-0290-7

10. Mody A, Eshun-Wilson I, Sikombe K, et al. Longitudinal engagement trajectories and risk of death among new ART starters in Zambia: A group-based multi-trajectory analysis. *PLoS Medicine*. 2019;16(10):1-25. doi:10.1371/journal.pmed.1002959

11. Ahoua L, Arikawa S, Tiendrebeogo T, et al. Measuring retention in care for HIV-positive pregnant women in Prevention of Mother-to-Child Transmission of HIV (PMTCT) option B+ programs: the Mozambique experience. *BMC public health*. 2020;20(1):322. doi:10.1186/s12889-020-8406-5

12. Agaba PA, Genberg BL, Sagay AS, et al. Retention in Differentiated Care: Multiple Measures Analysis for a Decentralized HIV Care and Treatment Program in North Central Nigeria. *Journal of AIDS & clinical research*. 2018;9(2). doi:10.4172/2155-6113.1000756

13. Luma HN, Eloumou SAFB, Fualefeh-Morfaw EA, et al. Anorectal pathology amongst HIV infected patients attending the Douala General Hospital: a cross-sectional study. *International Journal of STD & AIDS*. 2017;28(4):389-396. doi:10.1177/0956462416650817

14. Myer L, Phillips TK, Zerbe A, et al. Integration of postpartum healthcare services for HIV-infected women and their infants in South Africa: A randomised controlled trial. *PLoS medicine*. 2018;15(3):e1002547. doi:10.1371/journal.pmed.1002547

15. Fox MP, Bor J, Brennan AT, et al. Estimating retention in HIV care accounting for patient transfers: A national laboratory cohort study in South Africa. *PLoS Medicine*. 2018;15(6):e1002589. doi:http://dx.doi.org/10.1371/journal.pmed.1002589

16. Johnson LF, Estill J, Keiser O, et al. Do Increasing Rates of Loss to Follow-up in Antiretroviral Treatment Programs Imply Deteriorating Patient Retention? *American Journal of Epidemiology*. 2014;180(12):1208-1212. doi:10.1093/aje/kwu295

17. World Health Organization. *HIV Test–Treat–Retain Cascade Analaysis: Guide and Tools*.; 2017.

18. Poles G, Li M, Siril H, et al. Factors associated with different patterns of nonadherence to HIV care in Dar es Salaam, Tanzania. *Journal of the International Association of Providers of AIDS Care*. 2014;13(1):78-84. doi:10.1177/1545109712467068

19. Teklu AM, Yirdaw KD. Patients who restart antiretroviral medication after interruption remain at high risk of unfavorable outcomes in Ethiopia. *BMC health services research*. 2017;17(1):247. doi:10.1186/s12913-017-2172-9

20. Beres LK, Schwartz S, Simbeza S, et al. Patterns and Predictors of Incident Return to HIV Care Among Traced, Disengaged Patients in Zambia: Analysis of a Prospective Cohort. *Journal of acquired immune deficiency syndromes (1999)*. 2021;86(3):313-322. doi:10.1097/QAI.0000000000002554

21. Phillips TK, Orrell C, Brittain K, Zerbe A, Abrams EJ, Myer L. Measuring retention in HIV care: the impact of data sources and definitions using routine data. *AIDS (London, England)*. 2020;34(5):749-759. doi:10.1097/QAD.0000000000002478

22. Nawar E. Longitudinal analysis of interruptions in HIV care and treatment among HIV-positive pregnant women engaged in clinical care in Kigali, Rwanda. *Dissertation Abstracts International Section A: Humanities and Social Sciences*. 2020;81(7-A):No-Specified.

23. Sikkema KJ, Mulawa MI, Robertson C, et al. Improving AIDS Care After Trauma (ImpACT): Pilot Outcomes of a Coping intervention Among HIV-Infected Women with Sexual Trauma in South Africa. *AIDS and behavior*. 2018;22(3):1039-1052. doi:10.1007/s10461-017-2013-1

24. Health Resources and Services Administration HIV/AIDS Bureau. *Performance Measure : Medical Case Management : Medical Visits*. Vol 2006.; 2009.

25. Interagency Task Team on the Prevention and Treatment of HIV Infection in Pregnant Women Mothers and Children (IATT). *B + Monitoring & Evaluation Framework Dissemination and Country Consultation: Executive Summary of Technical Findings*.; 2015.

26. Gosset A, Protopopescu C, Larmarange J. Retention in Care Trajectories of HIV-Positive Individuals Participating in a Universal Test-and-Treat Program in Rural South Africa (ANRS 12249 TasP Trial ). *Journal of Acquired Immune Deficiency Syndromes*. 2019;80(4):375-385.

27. Henegar CE, Westreich D, Maskew M, et al. Comparison of pharmacy-based measures of adherence to antiretroviral therapy as predictors of virological failure. *AIDS and behavior*. 2015;19(4):612-618. doi:10.1007/s10461-014-0953-2

28. Anoje C, Agu KA, Oladele EA, et al. Adherence to On-Time ART Drug Pick-Up and Its Association with CD4 Changes and Clinical Outcomes Amongst HIV Infected Adults on First-Line Antiretroviral Therapy in Nigerian Hospitals. *AIDS and behavior*. 2017;21(2):386-392. doi:10.1007/s10461-016-1473-z

29. Joshi MP, Clark A, Ludman M. *Systems-Based Approaches to Improving Medication Adherence*.; 2016.

30. Kapiamba G, Masango T, Mphuthi D. Antiretroviral adherence and virological outcomes in HIV-positive patients in Ugu district, KwaZulu-Natal province. *African journal of AIDS research : AJAR*. 2016;15(3):195-201. doi:10.2989/16085906.2016.1170710

31. Court R, Leisegang R, Stewart A, et al. Short term adherence tool predicts failure on second line protease inhibitor-based antiretroviral therapy: an observational cohort study. *BMC infectious diseases*. 2014;14(1):664. doi:10.1186/s12879-014-0664-3

32. Mongo-Delis A, Mombo LE, Mickala P, et al. Factors associated with adherence to ARV treatment in people living with HIV/AIDS in a rural area (Koula-Moutou) in East Gabon. *African journal of AIDS research : AJAR*. 2019;18(1):51-57. doi:10.2989/16085906.2018.1552878

33. Atanga PN, Ndetan HT, Fon PN, et al. Using a composite adherence tool to assess ART response and risk factors of poor adherence in pregnant and breastfeeding HIV-positive Cameroonian women at 6 and 12 months after initiating option B+. *BMC pregnancy and childbirth*. 2018;18(1):418. doi:10.1186/s12884-018-2058-9

34. Mekuria LA, Prins JM, Yalew AW, Sprangers MAG, Nieuwkerk PT. Which adherence measure - self-report, clinician recorded or pharmacy refill - is best able to predict detectable viral load in a public ART programme without routine plasma viral load monitoring? *Tropical medicine & international health : TM & IH*. 2016;21(7):856-869. doi:10.1111/tmi.12709

35. Orrell C, Cohen K, Leisegang R, Bangsberg DR, Wood R, Maartens G. Comparison of six methods to estimate adherence in an ART-naïve cohort in a resource-poor setting: which best predicts virological and resistance outcomes? *AIDS Research & Therapy*. 2017;14(1):20. doi:10.1186/s12981-017-0138-y

36. Gachara G, Mavhandu LG, Rogawski ET, Manhaeve C, Bessong PO. Evaluating Adherence to Antiretroviral Therapy Using Pharmacy Refill Records in a Rural Treatment Site in South Africa. *AIDS research and treatment*. 2017;2017:5456219. doi:10.1155/2017/5456219

37. Denison JA, Koole O, Tsui S, et al. Incomplete adherence among treatment-experienced adults on antiretroviral therapy in Tanzania, Uganda and Zambia. *AIDS (London, England)*. 2015;29(3):361-371. doi:10.1097/QAD.0000000000000543

38. Hine P, Smith R, Eshun‐Wilson I, et al. Measures of antiretroviral adherence for detecting viral non‐suppression in people living with HIV. *Cochrane Database of Systematic Reviews*. 2018;(7). doi:10.1002/14651858.CD013080

39. Musumari PM, Wouters E, Kayembe PK, et al. Food insecurity is associated with increased risk of non-adherence to antiretroviral therapy among HIV-infected adults in the Democratic Republic of Congo: a cross-sectional study. *PloS one*. 2014;9(1):e85327. doi:10.1371/journal.pone.0085327

40. Abah IO, Ojeh VB, Musa J, et al. Clinical Utility of Pharmacy-Based Adherence Measurement in Predicting Virologic Outcomes in an Adult HIV-Infected Cohort in Jos, North Central Nigeria. *Journal of the International Association of Providers of AIDS Care*. 2016;15(1):77-83. doi:10.1177/2325957414539197

41. Denison JA, Packer C, Stalter RM, et al. Factors Related to Incomplete Adherence to Antiretroviral Therapy among Adolescents Attending Three HIV Clinics in the Copperbelt, Zambia. *AIDS and behavior*. 2018;22(3):996-1005. doi:10.1007/s10461-017-1944-x

42. Jennings L, Kellerman T, Spinelli M, et al. Drug resistance, rather than low tenofovir levels in blood or urine, is associated with tenofovir, emtricitabine and efavirenz (TEE) failure in resource-limited settings. *PREPRINT*. Published online 2021.

43. Genn L, Chapman J, Okatch H, et al. Pharmacy Refill Data are Poor Predictors of Virologic Treatment Outcomes in Adolescents with HIV in Botswana. *AIDS and Behavior*. 2019;23(8):2130-2137. doi:http://dx.doi.org/10.1007/s10461-018-2325-9

44. Alcaide ML, Ramlagan S, Rodriguez VJ, et al. Self-Report and Dry Blood Spot Measurement of Antiretroviral Medications as Markers of Adherence in Pregnant Women in Rural South Africa. *AIDS and behavior*. 2017;21(7):2135-2140. doi:10.1007/s10461-017-1760-3

45. Phillips TK, Sinxadi P, Abrams EJ, et al. A comparison of plasma efavirenz and tenofovir, dried blood spot tenofovir-diphosphate, and self-reported adherence to predict virologic suppression among South African women. *J Acquir Immune Defic Syndr*. 2019;81(3):311-318. doi:10.1097/QAI.0000000000002032

46. Erb S, Letang E, Glass TR, et al. A simple visual analog scale is a valuable tool to assess self-reported adherence in HIV-infected patients on antiretroviral treatment in a resource-limited setting. *Journal of AIDS and Clinical Research*. 2017;8(9):731. doi:http://dx.doi.org/10.4172/2155-6113.1000731

47. Hermans LE, Steegen K, Ter Heine R, et al. Drug level testing as a strategy to determine eligibility for drug resistance testing after failure of ART: a retrospective analysis of South African adult patients on second-line ART. *Journal of the International AIDS Society*. 2020;23(6):e25501. doi:10.1002/jia2.25501

48. Grabowski MK, Reynolds SJ, Kagaayi J, et al. The validity of self-reported antiretroviral use in persons living with HIV: A population-based study. *AIDS*. 2018;32(3):363-369. doi:10.1097/QAD.0000000000001706

49. Hirasen K, Evans D, Jinga N, et al. Using a Self-Administered Electronic Adherence Questionnaire to Identify Poor Adherence Amongst Adolescents and Young Adults on First-Line Antiretroviral Therapy in Johannesburg, South Africa. *Patient preference and adherence*. 2020;14:133-151. doi:10.2147/PPA.S210404

50. Buzibye A, Musaazi J, von Braun A, et al. Antiretroviral concentration measurements as an additional tool to manage virologic failure in resource limited settings: a case control study. *AIDS research and therapy*. 2019;16(1):39. doi:10.1186/s12981-019-0255-x

51. Mudhune V, Gvetadze R, Girde S, et al. Correlation of Adherence by Pill Count, Self-report, MEMS and Plasma Drug Levels to Treatment Response Among Women Receiving ARV Therapy for PMTCT in Kenya. *AIDS and behavior*. 2018;22(3):918-928. doi:10.1007/s10461-017-1724-7

52. Kimulwo MJ, Okendo J, Aman RA, et al. Plasma nevirapine concentrations predict virological and adherence failure in Kenyan HIV-1 infected patients with extensive antiretroviral treatment exposure. *PloS one*. 2017;12(2):e0172960. doi:10.1371/journal.pone.0172960

53. Abdulrahman SA, Ganasegeran K, Rampal L, Martins OF. HIV Treatment Adherence - A Shared Burden for Patients, Health-Care Providers, and Other Stakeholders. *AIDS reviews*. 2019;21(1):28-39. doi:10.24875/AIDSRev.19000037

54. Ngara B, Zvada S, Chawana TD, Stray-Pedersen B, Nhachi CFB, Rusakaniko S. A population pharmacokinetic model is beneficial in quantifying hair concentrations of ritonavir-boosted atazanavir: a study of HIV-infected Zimbabwean adolescents. *BMC Pharmacology and Toxicology*. 2020;21(58). doi:http://dx.doi.org/10.1186/s40360-020-00437-y

55. Johnston J, Wiesner L, Smith P, Maartens G, Catherine Orrell. Correlation of hair and plasma efavirenz concentrations in HIV-positive South Africans. *Southern African journal of HIV medicine*. 2019;20(1):1-6. doi:10.4102/sajhivmed.v20i1.881

56. Rhead R, Masimirembwa C, Cooke G, et al. Might ART Adherence Estimates Be Improved by Combining Biomarker and Self-Report Data? *PloS one*. 2016;11(12):e0167852. doi:10.1371/journal.pone.0167852

57. Drain P, National Institute of Allergy and Infectious Diseases (NIAID), Centre for the AIDS Programme of Research in South Africa. Simplifying Treatment and Monitoring for HIV. ClinicalTrials.gov.

58. Hickey MD, Salmen CR, Tessler RA, et al. Antiretroviral concentrations in small hair samples as a feasible marker of adherence in rural Kenya. *Journal of acquired immune deficiency syndromes (1999)*. 2014;66(3):311-315. doi:10.1097/QAI.0000000000000154

59. Murnane PM, Bacchetti P, Currier JS, et al. Tenofovir concentrations in hair strongly predict virologic suppression in breastfeeding women. *AIDS (London, England)*. 2019;33(10):1657-1662. doi:10.1097/QAD.0000000000002237

60. Tabb ZJ, Mmbaga BT, Gandhi M, et al. Antiretroviral drug concentrations in hair are associated with virologic outcomes among young people living with HIV in Tanzania. *AIDS (London, England)*. 2018;32(9):1115-1123. doi:10.1097/QAD.0000000000001788

61. Koss CA, Natureeba P, Mwesigwa J, et al. Hair concentrations of antiretrovirals predict viral suppression in HIV-infected pregnant and breastfeeding Ugandan women. *AIDS*. 2015;29(7):825-830. doi:10.1097/QAD.0000000000000619

62. Chawana TD, Nhachi CFB, Nathoo K, et al. Higher tenofovir concentrations in hair are associated with decreases in viral load and not self-reported adherence in HIV-infected adolescents with second line virological treatment failure. *AIDS research and human retroviruses*. Published online January 2021. doi:http://dx.doi.org/10.1089/AID.2020.0258

63. Gandhi M, Yang Q, Bacchetti P, Huang Y. Short Communication : A Low-Cost Method for Analyzing Nevirapine Levels in Hair as a Marker of Adherence in Resource-Limited Settings. 2014;30(1):10-13. doi:10.1089/aid.2013.0239

64. Chawana TD, Gandhi M, Nathoo K, et al. Defining a Cutoff for Atazanavir in Hair Samples Associated With Virological Failure Among Adolescents Failing Second-Line Antiretroviral Treatment. *Journal of acquired immune deficiency syndromes (1999)*. 2017;76(1):55-59. doi:10.1097/QAI.0000000000001452

65. George L, Muro EP, Ndaro A, Dolmans W, Burger DM, Kisanga ER. Nevirapine concentrations in saliva measured by thin layer chromatography and self-reported adherence in patients on antiretroviral therapy at Kilimanjaro Christian Medical Centre, Tanzania. *Therapeutic drug monitoring*. 2014;36(3):366-370. doi:10.1097/FTD.0000000000000005

66. L’Homme RFA, Muro EP, Droste JAH, et al. Therapeutic drug monitoring of nevirapine in resource-limited settings. *Clinical Infectious Diseases*. 2008;47(10):1339-1344. doi:10.1086/592694

67. Mungwira RG, Divala TH, Nyirenda OM, et al. A targeted approach for routine viral load monitoring in Malawian adults on antiretroviral therapy. *Tropical medicine & international health : TM & IH*. 2018;23(5):526-532. doi:10.1111/tmi.13047

68. Nnambalirwa M, Govathson C, Evans D, McNamara L, Maskew M, Nyasulu P. Markers of poor adherence among adults with HIV attending Themba Lethu HIV Clinic, Helen Joseph Hospital, Johannesburg, South Africa. *Transactions of the Royal Society of Tropical Medicine and Hygiene*. 2016;110(12):696-704. doi:10.1093/trstmh/trx003

69. Ongubo DM, Lim R, Tweya H, et al. A cross-sectional study to evaluate second line virological failure and elevated bilirubin as a surrogate for adherence to atazanavir/ritonavir in two urban HIV clinics in Lilongwe, Malawi. *BMC Infectious Diseases*. 2017;17(1):461. doi:http://dx.doi.org/10.1186/s12879-017-2528-0

70. Platt MO, Evans D, Keegan PM, et al. Low-Cost Method to Monitor Patient Adherence to HIV Antiretroviral Therapy Using Multiplex Cathepsin Zymography. *Molecular biotechnology*. 2016;58(1):56-64. doi:10.1007/s12033-015-9903-0

71. Eby J, Chapman J, Marukutira T, et al. The adherence-outcome relationship is not altered by diary-driven adjustments of microelectronic monitor data. *Pharmacoepidemiology and drug safety*. 2015;24(12):1313-1320. doi:10.1002/pds.3887

72. Harris RA, Haberer JE, Musinguzi N, et al. Predicting short-term interruptions of antiretroviral therapy from summary adherence data: Development and test of a probability model. *PloS one*. 2018;13(3):e0194713. doi:10.1371/journal.pone.0194713

73. Nguyen N, Robbins R, Courtney I, et al. Can Self-Reported Adherence Predict ART Adherence Assessed by an Electronic Monitoring Device (Wisepill) in Resource-Constrained Settings in Cape Town, South Africa? In: *Adherence 2019 Conference*. Vol 19. Adherence Conference 2019. United States. SAGE Publications Inc.; 2019:3-4. doi:http://dx.doi.org/10.1177/2325958219892705

74. Boussari O, Subtil F, Genolini C, et al. Impact of variability in adherence to HIV antiretroviral therapy on the immunovirological response and mortality Data analysis, statistics and modelling. *BMC Medical Research Methodology*. 2015;15(1). doi:10.1186/1471-2288-15-10

75. Ssewamala FM, Sensoy Bahar O, Nabunya P, et al. Suubi+Adherence-Round 2: A study protocol to examine the longitudinal HIV treatment adherence among youth living with HIV transitioning into young adulthood in Southern Uganda. *BMC public health*. 2021;21(1):179. doi:10.1186/s12889-021-10202-3

76. Kioko MT, Pertet AM. Factors contributing to antiretroviral drug adherence among adults living with HIV or AIDS in a Kenyan rural community. *African journal of primary health care & family medicine*. 2017;9(1):e1-e7. doi:10.4102/phcfm.v9i1.1343

77. Umar E, Levy JA, Bailey RC, Donenberg G, Hershow RC, Mackesy-Amiti ME. Virological Non-suppression and Its Correlates Among Adolescents and Young People Living with HIV in Southern Malawi. *AIDS and behavior*. 2019;23(2):513-522. doi:10.1007/s10461-018-2255-6

78. Guira O, Kaboré DSRR, Dao G, et al. The Modalities of Nonadherence to Highly Active Antiretroviral Therapy and the Associated Factors Related to Patients’ Sociodemographic Characteristics and Their Caregiving Perceptions in Ouagadougou (Burkina Faso). *Journal of the International Association of Providers of AIDS Care*. 2016;15(3):256-260. doi:10.1177/2325957415616492

79. Ketchaji A, Assah F, Fokam J, Tanue EA, Monebenimp F, Ngowe MN. Predictors of non-adherence to antiretroviral therapy among adolescents living with HIV in the Centre Region of Cameroon. *American Journal of Public Health Research*. 2019;7(4):126-136.

80. Okatch H, Beiter K, Eby J, et al. Brief Report: Apparent Antiretroviral Overadherence by Pill Count is Associated With HIV Treatment Failure in Adolescents. *Journal of acquired immune deficiency syndromes (1999)*. 2016;72(5):542-545. doi:10.1097/QAI.0000000000000994

81. Ssewamala FM, Byansi W, Bahar OS, et al. Suubi+Adherence study protocol: A family economic empowerment intervention addressing HIV treatment adherence for perinatally infected adolescents. *Contemporary clinical trials communications*. 2019;16:100463. doi:10.1016/j.conctc.2019.100463

82. Kalichman SC, Amaral CM, Cherry C, et al. Monitoring medication adherence by unannounced pill counts conducted by telephone: Reliability and criterion-related validity. *HIV Clinical Trials*. 2008;9(5):298-308. doi:10.1310/hct0905-298

83. McKinney O, Modeste NN, Lee JW, Gleason PC. Predicting Malawian Women’s Intention to Adhere to Antiretroviral Therapy. *Journal of public health research*. 2015;4(2):533. doi:10.4081/jphr.2015.533

84. Mbengue MAS, Chasela C, Onoya D, Mboup S, Fox MP, Evans D. Clinical predictor score to identify patients at risk of poor viral load suppression at six months on antiretroviral therapy: results from a prospective cohort study in Johannesburg, South Africa. *Clinical epidemiology*. 2019;11:359-373. doi:10.2147/CLEP.S197741

85. Giordano TP, Guzman D, Clark R, Charlebois ED, Bangsberg DR. Measuring adherence to antiretroviral therapy in a diverse population using a visual analogue scale. *HIV Clinical Trials*. 2004;5(2):74-79. doi:10.1310/JFXH-G3X2-EYM6-D6UG

86. Glass TR, De Geest S, Hirschel B, et al. Self-reported non-adherence to antiretroviral therapy repeatedly assessed by two questions predicts treatment failure in virologically suppressed patients. *Antiviral therapy*. 2008;13(1):77-85.

87. Mbengue MAS, Sarr SO, Diop A, Ndour CT, Ndiaye B, Mboup S. Prevalence and determinants of adherence to antiretroviral treatment among HIV patients on first-line regimen: A cross-sectional study in Dakar, Senegal. *Pan African Medical Journal*. 2019;33:95. doi:10.11604/pamj.2019.33.95.17248

88. Thorman J, Björkman P, Tesfaye F, Jeylan A, Balcha TT, Reepalu A. Validation of the Viral Load Testing Criteria - an algorithm for targeted viral load testing in HIV-positive adults receiving antiretroviral therapy. *Tropical medicine & international health : TM & IH*. 2019;24(3):356-362. doi:10.1111/tmi.13201

89. Magidson JF, Saal W, Nel A, Remmert JE, Kagee A. Relationship between depressive symptoms, alcohol use, and antiretroviral therapy adherence among HIV-infected, clinic-attending patients in South Africa. *Journal of health psychology*. 2017;22(11):1426-1433. doi:10.1177/1359105316628743

90. Kagee A, Nel A. Assessing the association between self-report items for HIV pill adherence and biological measures. *AIDS Care*. 2012;24(11):1448-1452. doi:10.1080/09540121.2012.687816

91. Wesevich A, Hosseinipour MC, Golin CE, et al. Female adherence self-efficacy before and after couple HIV testing and counseling within Malawi’s Option B+ program. *AIDS care*. 2020;32(2):170-174. doi:10.1080/09540121.2019.1634789

92. Nduaguba SO, Soremekun RO, Olugbake OA, Barner JC. The relationship between patient-related factors and medication adherence among Nigerian patients taking highly active anti-retroviral therapy. *African health sciences*. 2017;17(3):738-745. doi:10.4314/ahs.v17i3.16

93. Feldman B, Fredericksen R, Crane P, et al. Evaluation of the Single-Item Self-Rating Adherence Scale for Use in Routine Clinical Care of People Living with HIV. *AIDS & Behavior*. 2013;17(1):307-318. doi:10.1007/s10461-012-0326-7

94. Davis A, Norcini-Pala A, Nguyen N, et al. Longitudinal ART Adherence Trajectories and Sociodemographic and Psychosocial Predictors among ART Initiators in Cape Town, South Africa. In: *Adherence 2019 Conference*. International Association of Providers of AIDS Care; 2019.

95. Kekwaletswe CT, Morojele NK. Patterns and predictors of antiretroviral therapy use among alcohol drinkers at HIV clinics in Tshwane, South Africa. *AIDS care*. 2014;26 Suppl 1:S78-82. doi:10.1080/09540121.2014.906558

96. Byabene AK, Fortes-Déguénonvo L, Niang K, et al. Optimal antiretroviral therapy adherence as evaluated by CASE index score tool is associated with virological suppression in HIV-infected adults in Dakar, Senegal. *Tropical medicine & international health : TM & IH*. 2017;22(6):776-782. doi:10.1111/tmi.12882

97. Aregbesola OH, Adeoye IA. Self-efficacy and antiretroviral therapy adherence among HIV positive pregnant women in South-West Nigeria: a mixed methods study. *Tanzania Journal of Health Research*. 2018;20(4):unpaginated.

98. Mannheimer SB, Mukherjee R, Hirschhorn LR, et al. The CASE adherence index: A novel method for measuring adherence to antiretroviral therapy. *AIDS Care - Psychological and Socio-Medical Aspects of AIDS/HIV*. 2006;18(7):853-861. doi:10.1080/09540120500465160

99. Steel G, Nwokike J, Joshi MP. *Development of a Multi-Method Tool to Measure ART Adherence in Resource-Constrained Settings : The South Africa Experience*.; 2007.

100. Phillips TK, Wilson IB, Brittain K, et al. Decreases in Self-Reported ART Adherence Predict HIV Viremia Among Pregnant and Postpartum South African Women. *Journal of acquired immune deficiency syndromes (1999)*. 2019;80(3):247-254. doi:10.1097/QAI.0000000000001909

101. Phillips T, Brittain K, Mellins CA, et al. A Self-Reported Adherence Measure to Screen for Elevated HIV Viral Load in Pregnant and Postpartum Women on Antiretroviral Therapy. *AIDS and behavior*. 2017;21(2):450-461. doi:10.1007/s10461-016-1448-0

102. Wilson IB, Lee Y, Michaud J, FowlerJr FJ, Rogers WH. Validation of a New Three-Item Self-Report Measure for Medication Adherence. *AIDS Behaviour*. 2016;176(5):139-148. doi:10.1007/s10461-016-1406-x.Validation

103. Wilson I, Fowler F, Cosenza C, et al. Cognitive and Field Testing of a New Set of Medication Adherence Self-Report Items for HIV Care. *AIDS & Behavior*. 2014;18(12):2349-2358. doi:10.1007/s10461-013-0610-1

104. Meresse M, March L, Kouanfack C, et al. Patterns of adherence to antiretroviral therapy and HIV drug resistance over time in the Stratall ANRS 12110/ESTHER trial in Cameroon. *HIV medicine*. 2014;15(8):478-487. doi:10.1111/hiv.12140

105. Deschamps AE, Geest S De, Ph D, et al. Diagnostic Value of Different Adherence Measures Using Electronic Monitoring and Virologic Failure as Reference Standards. *AIDS Patient Care*. 2008;22(9). doi:10.1089/apc.2007.0229

106. Godin G, Gagné C, Naccache H. Validation of a self-reported questionnaire assessing adherence to antiretroviral medication. *AIDS Patient Care and STDs*. 2003;17(7):325-332. doi:10.1089/108729103322231268

107. Jackson IL, Umoh SS, Erah PO. Medication Adherence and Health Status in HIV Positive Patients in Akwa Ibom State, Nigeria. *Tropical Journal of Pharmaceutical Research*. 2020;19(10):2197-2204. doi:10.4314/tjpr.v19i10.25

108. Kripalani S, Risser J, Gatti ME, Jacobson TA. Development and evaluation of the Adherence to Refills and Medications scale (ARMS) among low-literacy patients with chronic disease. *Value in Health*. 2009;12(1):118-123. doi:10.1111/j.1524-4733.2008.00400.x

109. Kripalani S, Goggins K, Nwosu S, et al. Medication Non-Adherence before Hospitalization for Acute Cardiac Events. *Journal of Health Communication*. 2015;20(0):34-42. doi:10.1080/10810730.2015.1080331

110. Agala CB, Fried BJ, Thomas JC, et al. Reliability, validity and measurement invariance of the Simplified Medication Adherence Questionnaire (SMAQ) among HIV-positive women in Ethiopia: a quasi-experimental study. *BMC public health*. 2020;20(1):567. doi:10.1186/s12889-020-08585-w

111. Knobel H, Alonso J, Casado JL, et al. Validation of a simplified medication adherence questionnaire in a large cohort of HIV-infected patients: The GEEMA study. *AIDS*. 2002;16(4):605-613. doi:10.1097/00002030-200203080-00012

112. Chesney MA, Ickovics JR, Chambers DB, et al. Self-reported adherence to antiretroviral medications among participants in HIV clinical trials: The AACTG Adherence Instruments. *AIDS Care - Psychological and Socio-Medical Aspects of AIDS/HIV*. 2000;12(3):255-266. doi:10.1080/09540120050042891

113. Reynolds NR, Sun J, Nagaraja HN, Gifford AL, Wu AW, Chesney MA. Optimizing measurement of self-reported adherence with the ACTG adherence questionnaire: A cross-protocol analysis. *Journal of Acquired Immune Deficiency Syndromes*. 2007;46(4):402-409. doi:10.1097/QAI.0b013e318158a44f

114. Fedlu A, Alie B, Siraj Mohammed A, Adem F, Hassen A. Adherence to Antiretroviral Treatment for Prevention of Mother-to-Child Transmission of HIV in Eastern Ethiopia: A Cross-Sectional Study. *HIV/AIDS (Auckland, NZ)*. 2020;12:725-733. doi:10.2147/HIV.S274012

115. Nguyen H, VanZyl G, Geboers D, et al. Pharmacy refill data combined with self-report adherence questions improves prediction of boosted protease inhibitor regimen failure. In: *6th International Conference on HIV Treatment and Prevention Adherence*. ; 2011.

116. Kim MH, Tembo TA, Mazenga A, et al. The Video intervention to Inspire Treatment Adherence for Life (VITAL Start): protocol for a multisite randomized controlled trial of a brief video-based intervention to improve antiretroviral adherence and retention among HIV-infected pregnant women in . *Trials*. 2020;21(1):207. doi:10.1186/s13063-020-4131-8

117. Dewing S, Mathews C, Lurie M, Kagee A, Padayachee T, Lombard C. Predictors of poor adherence among people on antiretroviral treatment in Cape Town, South Africa: a case-control study. *AIDS Care*. 2015;27(3):342-349. doi:10.1080/09540121.2014.994471

118. Banas K, Lyimo RA, Hospers HJ, van der Ven A, de Bruin M. Predicting adherence to combination antiretroviral therapy for HIV in Tanzania: A test of an extended theory of planned behaviour model. *Psychology & health*. 2017;32(10):1249-1265. doi:10.1080/08870446.2017.1283037

119. Luszczynska A, Schwarzer R. Planning and Self-Efficacy in the Adoption and Maintenance of Breast Self-Examination : A Longitudinal Study on Self-Regulatory Cognitions SELF-EXAMINATION : A LONGITUDINAL STUDY. *Psychology and Health*. 2010;18(1):93-108. doi:10.1080/0887044021000019358

120. Barchi F, Winter SC, Ramaphane P, Dougherty D. The role of self-efficacy in women’s health-seeking behaviors in northwestern Botswana. *Journal of Health Care for the Poor and Underserved*. 2019;30(2):653-667. doi:10.1353/hpu.2019.0048

121. John ME, Samson-Akpan PE, Etowa JB, Akpabio II, John EE. Enhancing self-care, adjustment and engagement through mobile phones in youth with HIV. *International nursing review*. 2016;63(4):555-561. doi:10.1111/inr.12313

122. Hibbard JH, Stockard J, Mahoney ER, Tusler M. Development of the patient activation measure (PAM): Conceptualizing and measuring activation in patients and consumers. *Health Services Research*. 2004;39(4 I):1005-1026. doi:10.1111/j.1475-6773.2004.00269.x

123. Maclachlan EW, Shepard-Perry MG, Ingo P, et al. Evaluating the effectiveness of patient education and empowerment to improve patient-provider interactions in antiretroviral therapy clinics in Namibia. *AIDS care*. 2016;28(5):620-627. doi:10.1080/09540121.2015.1124975

124. Roter DL, Larson S. The Roter interaction analysis system (RIAS): Utility and flexibility for analysis of medical interactions. *Patient Education and Counseling*. 2002;(43):243-251. doi:10.1016/S0738-3991(02)00012-5

125. Kim E, Ndege PK, Jackson E, Clauw DJ, Ellingrod VL. Patient perspectives on medication self-management in rural Kenya: a cross-sectional survey. *International journal for quality in health care : journal of the International Society for Quality in Health Care*. 2019;31(5):353-358. doi:10.1093/intqhc/mzy187

126. Bailey SC, Annis IE, Reuland DS, Wolf MS. Development and evaluation of the Measure of Drug Self-Management. *Patient Preference*. 2015;9:1101-1108.

127. Denison JA, Banda H, Dennis AC, et al. ‘“ The sky is the limit ”’: adhering to antiretroviral therapy and HIV self-management from the perspectives of adolescents living with HIV and their adult caregivers. *Journal of the International AIDS Society*. 2015;18(19358):1-6. doi:10.7448/IAS.18.1.19358

128. Areri H, Marshall A, Harvey G. Factors influencing self-management of adults living with HIV on antiretroviral therapy in Northwest Ethiopia: a cross-sectional study. *BMC infectious diseases*. 2020;20(1):879. doi:10.1186/s12879-020-05618-y

129. Webel AR, Asher A, Cuca Y, et al. Measuring HIV self-management in women living with HIV/AIDS: A psychometric evaluation study of the HIV self-management scale. *Journal of Acquired Immune Deficiency Syndromes*. 2012;60(3):1-19. doi:10.1097/QAI.0b013e318256623d

130. Angwenyi V, Bunders-Aelen J, Criel B, Lazarus J V, Aantjes C. An evaluation of self-management outcomes among chronic care patients in community home-based care programmes in rural Malawi: A 12-month follow-up study. *Health & social care in the community*. Published online July 2020. doi:10.1111/hsc.13094

131. Lorig K, Laurent D. *Primer for Evaluating Outcomes*.; 2007.

132. Crowley T, Van der Merwe A, Kidd M, Skinner D. Measuring Adolescent HIV Self-management: An Instrument Development Study. *AIDS and behavior*. 2020;24(2):592-606. doi:10.1007/s10461-019-02490-z

133. Crowley T, van der Merwe A, Kidd M, Skinner D. Adolescent human immunodeficiency virus self-management: Associations with treatment adherence, viral suppression, sexual risk behaviours and health-related quality of life. *Southern African Journal of HIV Medicine*. 2020;21(1):1-11. doi:10.4102/SAJHIVMED.V21I1.1054

134. Chidrawi HC, Greeff M, Temane QM. Health behaviour change of people living with HIV after a comprehensive community-based HIV stigma reduction intervention in North-West Province in South Africa. *SAHARA J : journal of Social Aspects of HIV/AIDS Research Alliance*. 2014;11(1):222-232. doi:10.1080/17290376.2014.985700

135. Holzemer WL, Hudson A, Kirksey KM, Jane Hamilton M, Bakken S. The Revised Sign and Symptom Check-List for HIV (SSC-HIVrev). *Journal of the Association of Nurses in AIDS Care*. 2001;12(5):60-70. doi:https://doi.org/10.1016/S1055-3290(06)60263-X

136. Dlamini PS, Wantland D, Makoae LN, et al. HIV stigma and missed medications in HIV-positive people in five african countries. *AIDS Patient Care and STDs*. 2009;23(5):377-387. doi:10.1089/apc.2008.0164

137. Dodzo LG, Mahaka HT, Mukona D, Zvinavashe M, Haruzivishe C. HIV self-care practices during pregnancy and maternal health outcomes among HIV-positive postnatal mothers aged 18-35 years at Mbuya Nehanda maternity hospital. *AIDS care*. 2017;29(6):741-745. doi:10.1080/09540121.2016.1242710

138. Okoronkwo I, Ishaku S, Chinweuba A, Akpan-Idiok P, Ihudiebube C, Odira C. Assessing self care practices of people living with AIDS attending antiretroviral clinic Kafanchan, Kaduna State, Nigeria. *Journal of AIDS and Clinical Research*. 2015;6(12):528.

139. DeBruin M, Prins JM, Breukelen GJP Van. Self-Regulatory Processes Mediate the Intention-Behavior Relation for Adherence and Exercise Behaviors. *Health Psychology*. 2012;31(6):695-703. doi:10.1037/a0027425

140. Lyimo RA, Bruin M De, Boogaard J Van Den, Hospers HJ. Determinants of antiretroviral therapy adherence in northern Tanzania : a comprehensive picture from the patient perspective. *BMC Public Health*. 2012;12(716). doi:10.1186/1471-2458-12-716

141. Carver CS. You Want to Measure Coping But Your Protocol ’ s Too Long : Consider the Brief COPE. *International Journal of Behavioural Medicine*. 1997;4:92-100.

142. Folkman, Lazarus R. *Manual for the Ways of Coping Questionnaire*. Consulting Psychological Press; 1988.

143. Zerbe A, Brittain K, Phillips TK, et al. Community-based adherence clubs for postpartum women on antiretroviral therapy (ART) in Cape Town, South Africa: a pilot study. *BMC health services research*. 2020;20(1):621. doi:10.1186/s12913-020-05470-5

144. Sikazwe I, Eshun-Wilsonid I, Sikombe K, et al. Retention and viral suppression in a cohort of HIV patients on antiretroviral therapy in Zambia: Regionally representative estimates using a multistage-sampling-based approach. *PLoS Medicine*. 2019;16(5):1-17. doi:10.1371/journal.pmed.1002811

145. Reepalu A, Balcha TT. Development of an algorithm for determination of the likelihood of virological failure in HIV-positive adults receiving antiretroviral therapy in decentralized care. *Global Health Action*. 2017;10(01). doi:10.1080/16549716.2017.1371961

146. Murnane PM, Brown ER, Donnell D, et al. Estimating efficacy in a randomized trial with product nonadherence: application of multiple methods to a trial of preexposure prophylaxis for HIV prevention. *American journal of epidemiology*. 2015;182(10):848-856. doi:10.1093/aje/kwv202

147. Umar E. ART adherence among adolescents & young people living with HIV in Southern Malawi: A conditional process analysis. *Dissertation Abstracts International: Section B: The Sciences and Engineering*. 2018;79(10-B(E)):No-Specified.

148. Esber A, Polyak C, Kiweewa F, et al. Persistent Low-level Viremia Predicts Subsequent Virologic Failure: Is It Time to Change the Third 90? *Clinical infectious diseases : an official publication of the Infectious Diseases Society of America*. 2019;69(5):805-812. doi:10.1093/cid/ciy989

149. Platt L, Xu A, Giddy J, et al. Identifying and predicting longitudinal trajectories of care for people newly diagnosed with HIV in South Africa. *PLoS ONE*. 2020;15(9):e0238975. doi:10.1371/journal.pone.0238975

150. Boussari O, Subtil F, Genolini C, et al. Impact of variability in adherence to HIV antiretroviral therapy on the immunovirological response and mortality Data analysis, statistics and modelling. *BMC Medical Research Methodology*. 2015;15(1). doi:10.1186/1471-2288-15-10

151. Holmes WC, Shea JA. A New HIV/AIDS-Targeted Quality of Life (HAT-QoL) Instrument. *Medical Care*. 1998;36(2):138-154.
