# Supplementary material for: Measuring patient engagement with HIV care in sub‐Saharan Africa: a scoping study
Source: J Int AIDS Soc. 2022 Oct 26;25(10):e26025. doi: 10.1002/jia2.26025 (PMC9597383; doi:10.1002/jia2.26025)
Supplement: Supplementary file 2 — File S1: Members of the InCARE Stakeholder Group. [file JIA2-25-e26025-s001.docx]

**File S1**: Members of the InCARE Stakeholder Group.

1. Anna Grimsrud: Lead Technical Advisor, International AIDS Society

2. Beth Harley: HIV, AIDS, sexually transmitted infections and tuberculosis

(HAST) medical officer for City of Cape Town, South Africa

3. Erin von der Heyden: HIV, AIDS, sexually transmitted infections and

tuberculosis (HAST) medical officer for the Khayelitsha and Eastern Substructure

of theWestern Cape Department of Health, South Africa

4. Ingrid Eshun-Wilson: Family physician and epidemiologist, School of

Medicine, Washington University, United States of America and University

of Stellenbosch, South Africa

5. Ingrid Katz: Physician and Associate Faculty Director, Harvard Global

Health Institute, Harvard University, United States of America

6. Jonathan Euvrard: Epidemiologist at the Centre for Infectious Disease Epidemiology

and Research (CIDER) and PhD student at the University of Cape

Town, South Africa

7. Kirsten Arendse:HIV Medical Research Manager for the “Welcome Service”

(A differentiated service delivery model for patients who interrupt ART) for

Médecins Sans Frontières’ Khayelitsha project, South Africa

8. K Rivet Amico: Associate Professor,Health Behaviour andHealth Education,

School of Public Health, University of Michigan, United States of America

9. Laura Beres: Assistant Scientist at Johns Hopkins Bloomberg School of

Public Health, Department of International Health, Johns Hopkins, United

States of America

10. Michael Mugavero: Director for the Center for Outcomes Effectiveness

Research and Education (COERE), Co-Director of the Center for AIDS

Research, and PI of an AHRQ T32 training grant in health services and

outcomes research, University of Alabama at Birmingham, United States of

America

11. Tali Cassidy: HIV Epidemiologist for Médecins Sans Frontières’ Khayelitsha

project and Division of Public Health Medicine, School of Public Health and

Family Medicine, University of Cape Town, South Africa

12. Tamsin Phillips: Senior Lecturer in Division of Epidemiology & Biostatistics,

School of Public Health & Family Medicine, University of Cape Town, South

Africa
